# Cognitive mechanisms underlying the recall of positive relationships in social networks

**DOI:** 10.1038/s41598-026-57381-w

**Published:** 2026-06-17

**Authors:** Zoran Kovacevic, Christoph Stadtfeld

**Affiliations:** 1 Social Networks Lab, ETH Zurich, Stampfenbachstr. 69, 8092 Zurich, Switzerland; 2 Corvinus Institute of Advanced Studies, Corvinus University, Budapest, Hungary

**Keywords:** Social networks, Social relations, Social memory, Recall sequences, Mental representation, Mathematics and computing, Neuroscience, Psychology, Psychology

## Abstract

Humans are embedded in complex social networks. To utilize them, they regularly need to recall with whom they are connected. This recall is cognitively demanding and utilizes a variety of heuristic cognitive mechanisms. We empirically investigate traces of these mechanisms in the context of free and repeated recall of positive social relationships (friendships, positive interactions) within an emerging undergraduate student community (*N = 820* participants). Applying multinomial modeling, we study the specific sequences in which individuals recalled their peers when prompted in a repeated online survey. We demonstrate that recall sequences are governed by multidimensional aspects: demographic similarity, shared social contexts, relationship quality, and social visibility. These aspects are relevant for the relation between the nominator and nominee but also for the associations between consecutively recalled individuals. Post-hoc analyses suggests that these patterns are stable across individuals and network types, but vary within sequences and as the community matures.

## Introduction

Humans are embedded in complex social networks. These networks can be beneficial in many situations of their lives^1–7^. However, in order to activate and utilize their social relationships in a specific situation, they must be able to correctly and efficiently recall with whom and how they are connected. This is the case, when people think who they can ask for help or advice, who to invite to a work project or dinner party, who to share information with, or whom they know in a given social context. In many situations, correct and fast recall of relationships is a crucial ability in humans’ social lives. But recalling social relationships is not a trivial cognitive task as it involves searching through the memories of one’s own complex social network.

In this article, we ask what cognitive strategies humans use to search their memory and recall social ties (social recall) in real-life situations. Similarly to other complex cognitive tasks, it has been proposed that humans use various cognitive mechanisms—termed schemas^8^, heuristics^9^, or association patterns^10^—to navigate the memory space and deal with the cognitive demand to recall relationships^11–13^. These mechanisms use cues or stimuli to which the recalling individual (hereafter, *ego*) is currently exposed and that are related to the object of recall. These can include their current mood, visual perceptions, and spatial contexts^13–16^.

In case of social recall, relevant cues will further include ego’s perceptions of social contexts and structures they are embedded in, and their own social identity and individual attributes^17–19^. When recalling multiple individuals (hereafter, *alters*), the previous recall enters the set of available cues for the next recall^20–22^. Newly available cues supporting the recall of another alter can include the demographic and structural characteristics of the alter recalled previously, as well as the social contexts in which they are jointly embedded^17,23^. The cues used in social recall (social cues) can vary in complexity. In the most basic case, ego may just consider individuals’ demographic attributes. In more complex cases, they may use as cues the experienced occasions of interactions in different social contexts such as spaces, activities, or groups. They may further consider overarching qualities and types of relations between ego and alter and between two alters. Finally, they could rely on more abstract concepts derived from their perception of the overall social environments like individuals’ social visibility, status, or network position.

Social cues can have two sources of origin: Ego itself or a previously recalled alter. We can therefore observe two main types of patterns in the nomination sequence. If the source of the cue is based on ego, certain alters will generally be recalled first or earlier in the process (e.g., those with the same demographic attribute as ego). We refer to these patterns as *“serial order recall”*, as they explain which alters appear earlier in a given recall sequence^23^. If the source of the cue is the previously recalled alter, this will be reflected by certain pairs of individuals being recalled one after the other (e.g., two individuals who are demographically similar but potentially different from the ego). We refer to these patterns as *“association recall”*, as they explain which alters are associated by the ego and thus recalled one after the other.

We therefore consider four classes of mechanisms that make use of increasingly complex social cues. These can be tracked by two observable patterns—serial order recall and association recall:

1. Demographic similarities
2. Social and spatial contexts
3. Relationship qualities
4. Characteristics relating to the social visibility, status, or position

The proposed classification uses structural features of the type of cues and is based on classifications of structural network mechanisms in social networks^24^. At the same time, it covers a variety of cognitive mechanisms discussed in the context of related classifications in social psychology and cognitive science^9,17,23^.

**Fig. 1:**
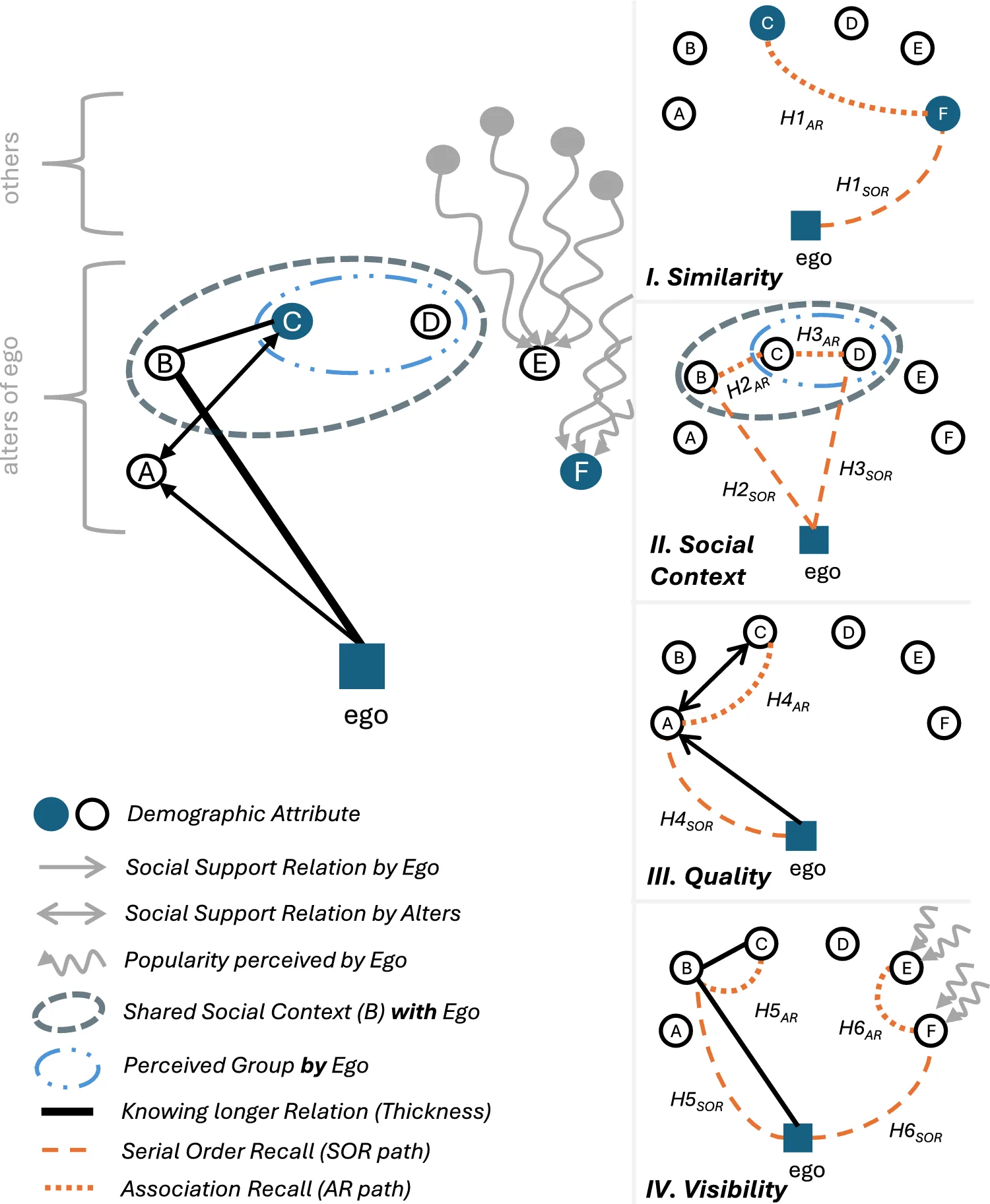
Conceptual framework of social structure and recall mechanisms. The left panel exemplifies the embedding of a focal individual (ego) in perceived social structures. Individuals who are recalled as members of their social network (alters)—in our case as friends or positive interaction partners—are shown as circles A–F. The right panel illustrates examples of the four mechanisms from ego’s perspective. Serial order recall patterns (dashed lines; SOR) relate to ego naming alters earlier, association patterns (dotted lines; AR) to naming two alters after one another. Labels H1 to H6 indicate hypotheses shown in Table 1. Panels (**i**)–(**iv**) illustrate how (**i**) demographic similarity, (**ii**) shared social contexts and groups with ego and between alters, (**iii**) “stronger” ties, indicated by individuals perceiving each other as sources of various dimensions of social support, and (**iv**) social visibility, defined via age of the relationship and the perceived popularity of the alters, may affect SOR and AR recall patterns, respectively.

Figure 1 provides an overview of exemplary cognitive mechanisms in each class and the connected social cue. The first class of mechanisms concerns only the demographic similarities between ego and recalled alters, as well as between alters. Previous work has found that shared attributes, such as gender or language, are important cues in specific settings for mentally organizing social structures^25,26^ and for the social recall^27,28^. The second class focuses on observable occasions of social interaction across various social or spatial contexts, such as activities or groups that multiple individuals may share. Previous work theorized that triadic embeddedness^9^, shared social groups and cliques^29–31^, as well as shared spatial locations^32^, may serve as cues in social recall. Furthermore, social roles occupied by the recalled person^33,34^ and an overall social proximity between individuals, including, among others, the amount of shared social contexts, seem to serve as cues^17,23^. It should be noted that complex social groups are not just social contexts, but may themselves be the result of similarity mechanisms, for example, when groups are initially formed based on demographic similarity. The third class concerns the overarching quality and types of pairwise relationships between two individuals, either ego and alter or two alters. Previous work discussed that social recall may focus on a specific type of relations^35^ and consider their strength and importance^9,35–37^. The fourth class is based on abstract structural social characteristics of alters, such as their general social visibility, their social status, or their type of position in ego’s social network. Previous work has argued that distance, (psychological) visibility^10,35,38^ and hierarchical structures in the network^39–41^, are relevant for accurate observation of social structures and also for recall. Social visibility is an abstract concept that can partly overlap with the similarity mechanisms above. This is the case when, for example, individuals with similar social status are recalled. The fact that that some complex mechanisms do not strictly fall into one of the classes is an important argument for the multivariate investigation presented below.

Evidence for the existence of cognitive mechanisms can be indirectly derived from the sequences in which an individual recalls their alters. Which alters are recalled first or early in the process, and which pairs of alters are recalled after one another? We use data collected in a detailed longitudinal social network study on an emerging undergraduate student community at a Swiss university ($N_{\textit{ego}}$ = 820 individuals, $N_\textit{community}$ = 1,147 individuals)^42^. Specifically, we study patterns in the recall of two positive relationships within the community: friendship and positive interactions. By situating our study in a real-life social setting, we can test a variety of patterns that were identified in previous, partly experimental or vignette-based research with increased ecological validity and in the closed context of an emerging community. Due to the complete network study design, we are able to rely on information beyond ego itself but reported by others in the study. This includes relationships reported between alters that may be visible to ego, as well as alters’ self-reported demographics, their activities, and their social contexts. Finally, we can compare mechanisms across two types of social networks that appear both meaningful in the empirical context, one of which (friendship) is widely used in empirical social network studies^43^. Friendship and positive interactions were selected as network measures as they capture nested, relational concepts. Friendship is the more specific and stronger type of relationship. It is expected to include positive interactions—defined as regular or occasional instances of positive interactions—but additionally entails emotional connection, mutual affection and behavior, and normative expectations^43^. Comparison of the two nested relationships allows us to determine whether our findings are inherent to the specific definition of the recalled relationship or applicable to positive ties more broadly.

**Table 1:** Overview of the hypotheses. A visual representation of some is shown in Fig. 1.

Category	$\hbox {Hypothesis}^{1}$	Description
	*Serial order pattern hypothesis (SOR):*	
	*People tend to nominate earlier people ...*	
Similarity	H1$_{\text {SOR}}$	... with the same major demographic dimensions (gender, nationality, mother tongue) as themselves$^{2}$
Social context	H2$_{\text {SOR}}$	... to whom they have relations in more different contexts (study, travel, free time)$^{2}$
	$\textrm{H3}_{{\text {SOR}}^{3}}$	... who are perceived as a member of a group by them
Relationship quality	H4$_{\text {SOR}}$	... to whom they have a more supportive and close connection (social support)
Social Visibility	H5$_{\text {SOR}}$	... who they have known longer
	H6$_{\text {SOR}}$	... who they perceive as popular
	*Association pattern hypothesis (AR):*	
	*Participants tend to recall pairs of individuals ...*	
Similarity	H1$_{\text {AR}}$	... based on the same major demographic dimensions (sex, nationality, mother tongue)$^{2}$
Social context	H2$_{\text {AR}}$	...based on specific contexts (studying, traveling, free time)$^{2}$
	H3$_{\text {AR}}$	... based on perceived groups
Relationship quality	H4$_{\text {AR}}$	... who have a more supportive and close connection to each other (social support)
Social visibility	H5$_{{\text {AR}}^{3}}$	... who know each other longer
	H6$_{{\text {AR}}^{3}}$	... who are both perceived as popular
Name overlap$^{4}$	H7$_{\text {AR}}$	... who have a similar name
	*Hypothesis about consistency*	
	H8	Comparing the results of the name generator for friendship with the results of the name generator for positive interaction, the same patterns emerge

$^{1}$ For deviations from the preregistration please check Supplementary Table 1.

$^{2}$ Each individual aspect of this dimension was included in the models as its own predictor to disentangle the effects from each other.

$^{3}$These hypotheses were not preregistered and were incorporated for the purpose of serving as counterparts (Serial Order vs. Association Pattern) to avoid potential misinterpretation of the preregistered hypotheses.

$^{4}$This mechanism was included as methodological control for potential methodological artifacts in our data collection and does not represent a hypothesized cognitive mechanism. For more details, see Section "Name overlap"

Table 1 provides an overview of our pre-registered hypotheses^44^. We use multilevel conditional multinomial logit models to statistically test whether there is evidence for the hypothesized serial order and association recall patterns. For each individual, the recall process is analyzed as a sequence of multinomial probabilities. At each step, the ego ’chooses’ whom to nominate next from a pool of available alters—defined as the alters who appear in the total sequence but have not yet been mentioned. Then we test in a multivariate model, which patterns related to a given mechanism are associated with a higher probability of being nominated from this available pool in this specific selection event. We do not interpret choices as a utility maximization process in a strict econometric sense, but rather as behavioral outcomes of an unobserved cognitive process that can reveal patterns related to cognitive heuristics.

## Results

**Fig. 2:**
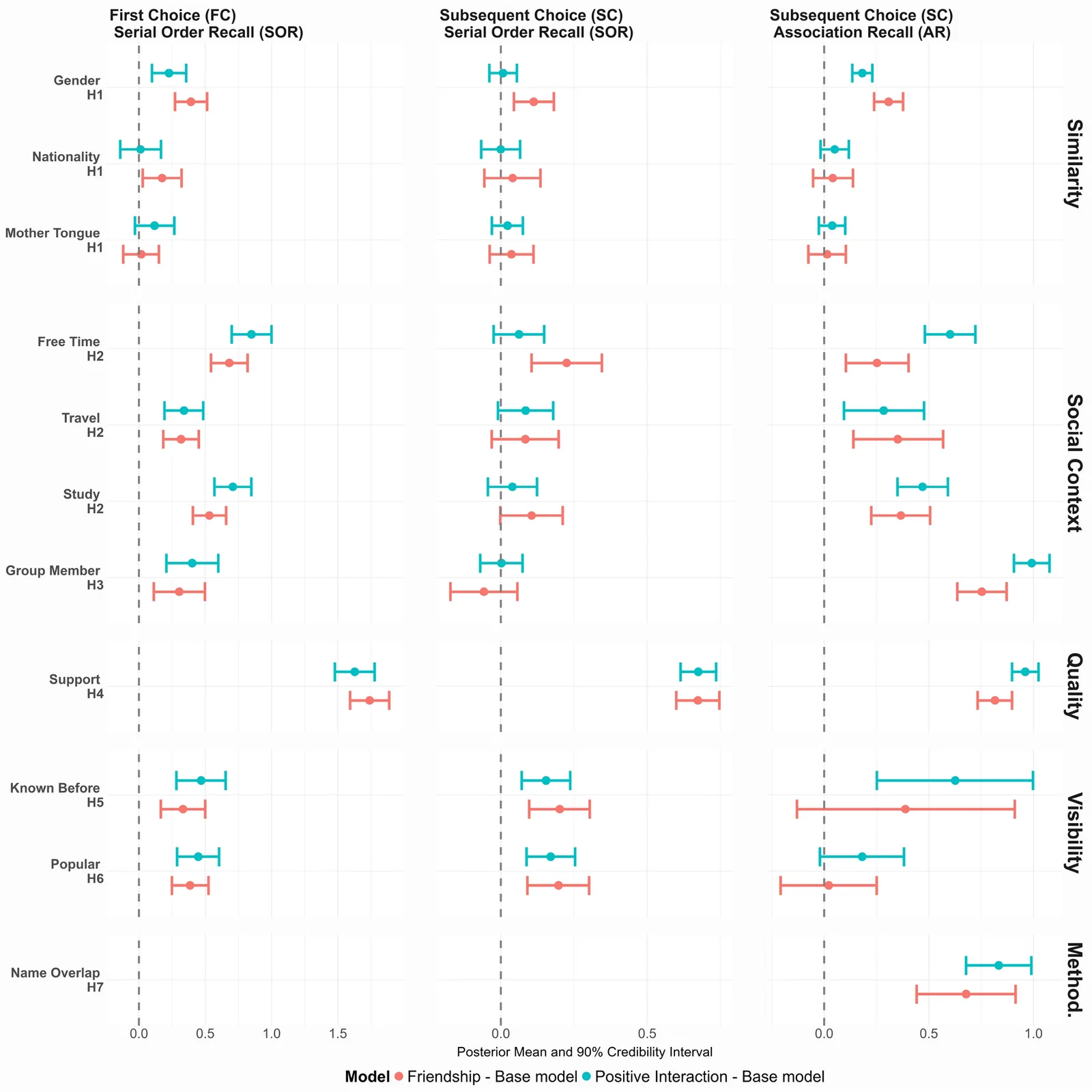
Credibility intervals and posterior means for the parameters of the main models. The dot represents the posterior mean. The horizontal lines show the 90% credibility intervals. The models are based on around 2000 unique choices for each relation type for initial recall (FC) and around 10,000 choices for subsequent recall (SC). The red distributions (bottom ones) are related to the model based on the friendship relations, the blue ones (top ones) are related to the model based on positive interaction relations. The x-axis limits are not identical between the plots to provide a clearer overview.

We analyze the recall sequences of students’ answers to two survey questions about their friendships and positive interactions within an emerging community of undergraduate students, considering surveys at multiple time points over the course of three undergraduate years. The reported results are based on a stepwise multinomial choice model, which conceptualizes the process of answering a network question as a series of discrete choices in which egos select the next person from the set of not yet recalled and known alters. We report the first choice in a separate model as it only includes serial order recall mechanisms. All subsequent choices are considered in a model that additionally includes association recall mechanisms that use social cues from the previously recalled alter. The results of the main models for the hypotheses described in Table 1 are reported in Fig. 2. To ensure the stability of these findings, we conducted several robustness checks accounting for the multilevel structure of the data (observations nested within time points and individuals), alongside sensitivity analyses, alternative imputation strategies, and additional exploratory analyses. While uncorrected formal model fit comparisons initially favored more complex specifications—such as those incorporating individual-level random effects—corrected estimates suggest that these gains are not statistically meaningful (see Supplementary Sections 9–11). Together with the qualitative result that the substantive findings remain highly consistent across all specifications, we focus in the following our results on the more parsimonious main models. We first present the main results and then discuss specific insights generated by the large pool of robustness and sensitivity analyses. All additional full model details and results are provided in Supplementary Sections 13–16. Additional to the main models results, an initial descriptive investigation using transition matrices (see Supplementary Section 4) and the adjusted ratio of clustering (see Supplementary Section 5), suggested weak clustering tendencies in the nominations based on gender, relationship quality, and social context. However, these results are inconclusive and limited by the relatively short nomination sequences used in this study and their inability to incorporate multiple explanatory variables simultaneously.

### Patterns in the recall sequences

Results of the main multinomial choice models are presented in Fig. 2. The numerical values of all their coefficients and their credible intervals – based on 90%, 95%, and posterior probability—are reported in Supplementary Section 3 and are partly reported below. The first column shows a model testing serial order mechanisms for the alters who are recalled first (first choice, labeled FC). The second and third columns represent a model for all subsequently recalled alters (subsequent choice, labeled SC). The mechanisms reflect serial order recall mechanisms (column 1 & 2, labeled SOR) or association recall mechanisms (column 3, labeled AR). The order of coefficients follows the proposed classification of mechanisms, using similarity, social context, relationship quality, social visibility, and, as a methodological control, name overlap between alters. For each mechanism and network type, the posterior means and credibility intervals (CI) of the estimated coefficients are presented. In the following text, the results are reported as $\beta = \text {mean} \;[\text {lower bound}, \text {upper bound}]$, where the mean represents the posterior average and the brackets denote the lower and upper bounds of the 90% CI. We specify the network related to the coefficients with ’F’ for friendship and ’PI’ for positive interaction. Within a given model, results are interpreted based on coefficient magnitudes and their corresponding CIs. When comparing effects across models, we restrict our interpretation exclusively to the sign of the parameters and whether their CIs contain zero.

The *similarity* mechanisms (H1) consider three types of cues: individuals’ gender, nationality, and mother tongue. We find a positive tendency to nominate alters with the same gender, which influenced serial order recall in both first ($\beta _{\text {F}} = 0.39 \; [0.27, 0.51]$; $\beta _{\text {PI}} = 0.23 \; [0.10, 0.36]$) and subsequent choices (ego naming others of the same gender first and earlier; $\beta _{\text {F}} = 0.11 \; [0.04, 0.18]$) and association recall (ego subsequently naming alters with the same gender; $\beta _{\text {F}} = 0.31 \; [0.24, 0.38]$; $\beta _{\text {PI}} = 0.18 \; [0.13, 0.23]$). The association is found for both types of relationships and both types of mechanisms, except for positive interaction in the case of serial order recall mechanisms in subsequent choices (SC-SOR; $\beta _{\text {PI}} = 0.01 \; [-0.04, 0.05]$). For similarity in nationality, a positive trend to nominate alters was detected for the serial order recall in the first choice of friendship relations (FC-SOR; $\beta _{\text {F}} = 0.17 \; [0.03, 0.32]$). This finding, however, was not robust and disappeared in key robustness checks. Similarity based on mother tongue did not show evidence for an effect in the models.

The *social context* mechanisms (H2 & H3) use as cues the overlap in three social contexts (shared free-time activities, traveling together, studying together) and being perceived as members of the same group. Across both relation types, there was a clear tendency towards serial order recall in first choices (FC-SOR; e.g., $\beta _{\text {F: Free Time}} = 0.68 \; [0.54, 0.82]$; $\beta _{\text {F: Study}} = 0.53 \; [0.41, 0.66]$; $\beta _{\text {F: Travel}} = 0.32 \; [0.18, 0.45]$, $\beta _{\text {F: Group Member}} = 0.30 \; [0.11, 0.50]$), and association recall in the subsequent choices (SC-AR; e.g., $\beta _{\text {F: Free Time}} = 0.25 \; [0.10, 0.40]$; $\beta _{\text {F: Study}} = 0.37 \; [0.23, 0.51]$; $\beta _{\text {F: Travel}} = 0.35 \; [0.14, 0.57]$, $\beta _{\text {F: Group Member}} = 0.75 \; [0.64, 0.87]$). This indicates that egos recalled first those with whom they share social contexts and subsequently recalled pairs of alters who share social contexts with each other. For subsequent nominations, the serial order recall effects were less consistent (SC-SOR). Here, we only found an effect for shared free-time activities on the serial order recall of friendships ($\beta = 0.22 \; [0.10, 0.35]$). Considering the size of the parameters and their consistency across a variety of robustness analyses, they were found to be highly influential, especially in association recall. For association recall, shared group membership emerged as one of the strongest predictors, based on the size of the parameters ($\beta _{\text {F}} = 0.75 \; [0.64, 0.87]$, $\beta _{\text {PI}} = 0.99 \; [0.91, 1.08]$).

The *relationship quality* mechanisms (H4) use as cues whether individuals, in addition to being friends or positive interaction partners, perceive each other as a source of social support, which was collected as another network item in the same survey. In all models, the relationship quality showed robust, strong positive tendencies in predicting the nominated alters. This indicates that social support ties between ego and alters were associated with first and early recall ($\beta _{\text {F - FC}} = 1.74 \; [1.59, 1.89]$, $\beta _{\text {PI - FC}} = 1.63 \; [1.48, 1.78]$, $\beta _{\text {F - SC}} = 0.67 \; [0.60, 0.75]$, $\beta _{\text {PI - SC}} = 0.67 \; [0.61, 0.74]$), and social support ties between alters with association recall patterns ($\beta _{\text {F}} = 0.82 \; [0.73, 0.90]$, $\beta _{\text {PI}} = 0.96 \; [0.91, 1.08]$). For the serial order recall in both networks and both for first and subsequent choices, social support was the strongest overall predictor based on parameter size.

The *social visibility* mechanisms (H5 and H6) uses as cues whether individuals had known each other already before they joined university, and whether individuals perceive others to be popular members of the student community. There are positive tendencies for nominating alters in the serial order recall for initial (FC-SOR; e.g., $\beta _{\text {F: Known Before}} = 0.33 \; [0.16, 0.50]$, $\beta _{\text {F: Popular}} = 0.39 \; [0.25, 0.52]$) and subsequent choices in both relation types (SC-SOR; e.g., $\beta _{\text {F: Known Before}} = 0.20 \; [0.10, 0.30]$, $\beta _{\text {F: Popular}} = 0.20 \; [0.09, 0.30]$). For association recall in subsequent nominations (SC-AR), social visibility aspects were in general not detected, with the exception of a positive tendency to nominate alters which ego knows longer in the positive interaction network ($\beta = 0.63 \; [0.25, 1.00]$); however, this effect was not stable across the pool of robustness models.

For the methodological control aspect of name overlap (H7), we find a robust and strong positive influence on the tendency to be nominated ($\beta _{\text {F}} = 0.68 \; [0.44, 0.91]$, $\beta _{\text {PI}} = 0.83 \; [0.68, 0.99]$). There were only few empirical cases of name overlaps within alter sets (263 cases), indicating that the effect appears strong when it applies, although it is not often observed in general.

In general, the patterns in the recall of friendship ties were similar to those when recalling social interaction partners with smaller differences in terms of effect size and significance, as discussed above. The results are in line with our hypothesis on the *consistency of patterns* (H8) across different network recall tasks.

### Robustness of patterns

The result presented above required a number of decisions and simplifications in the statistical modeling of the data. Here, we investigate the stability of these patterns under alternative modeling decisions in an extensive series of sensitivity and robustness analyses. These are presented in detail in the Supplementary (from Supplementary Figs. 13 to 16). Methodological aspects of model choice and comparisons are discussed in Section "Robustness".

First, we conducted a sensitivity analysis of the chosen Bayesian priors. The core findings demonstrated general robustness across various prior specifications, including the use of weaker priors and regularized horseshoe priors (see e.g., Supplementary Fig. 13.1 and 13.2).

Second, we investigated alternative treatments of missing observations (see Section "Robustness"), particularly MICE imputation or list-wise deletion of incomplete choices (reduced dataset). Models employing the MICE dataset and the reduced choice dataset to account for missing data primarily preserved the initial nomination results (see e.g., Supplementary Fig. 13.7 and 13.8). For subsequent nominations, both methods introduced a weak positive tendency in the serial order recall to nominate alters from some social contexts (mostly free time and studying). Notably, serial order recall based on group membership became negative in these models, which might indicate a potential tendency to avoid nomination overlaps of group memberships if they are not rooted in social contexts. Furthermore, both methods showed weak positive tendencies for association recall based on knowing before (social visibility) and serial order recall for nationality similarity.

Third, we estimated models that consider multilevel structures in the data (within egos, within time points, and within sequences) and other sources of heterogeneity. Models incorporating individual-level random slopes separately for each predictor did not alter the core findings for initial nominations (see e.g., Supplementary Fig. 13.6), but revealed additional positive tendencies in serial order recall related to specific social contexts: studying for friendship and travel for positive interaction.

Models incorporating time-related interactions (see e.g., the interactions for association recall of support or group membership in Supplementary Fig. 15.8 or Fig. 16.4) showed first evidence for an increasing relevance of the relationship quality-related association recall mechanism robustly across later measurement waves (specifically waves 4–6), with similar tendencies, though less conclusive, for the group-related social context dimension. Overall, various mechanisms showed some time dynamics, but often without sufficient confidence and clear trends. Between sequences, serial order recall and association recall based on relationship quality and group membership were stronger for longer sequences (see e.g., Supplementary Fig. 13.5). Within sequences, these effects were moderated by position: the serial order recall based on relationship quality decreased at later positions within the sequence for both relation types. For friendship, serial order recall based on shared gender also decreased after the third position. Multiple other tendencies emerged, but with insufficient confidence (see e.g., Supplementary Fig. 15.6). Models combining time and choice dynamics, support the separate model results (see e.g., Supplementary Fig. 15.8).

Finally, we estimated models with additional exploratory predictors or different operationalisations of included predictors (see e.g., Supplementary Fig. 13.9). These include similarity based on home canton, network reciprocity, frequency of interaction, and alternative operationalisations of social context and name overlap. The inclusion did not substantially alter the core results. However, some predictors (e.g., nationality similarity and knowing before) are found to be influenced by the additional predictor included, highlighting that some of the used predictors may be approximating a broader, unmeasured dimension of the relationship. The predictors included to evaluate different operationalisations of the model predictors did not yield different conclusions.

## Discussion

This study investigates the cognitive mechanisms underlying social recall by analyzing sequential nominations within an ecological social network. Situated in an emerging community of undergraduate students, we examined recall patterns in distinct positive relationship types (friendship and positive interaction). The empirical tests utilize the fact that detailed social, contextual, and demographic information is known both for the recalling individuals (egos) and those individuals they recalled as parts of their social networks (alters).

We demonstrate that their recall follows systematic, theoretically derived patterns, which were formulated in a variety of pre-registered hypotheses (see Fig. 1). Specifically, egos navigate social recall via an interplay of cognitive mechanisms—primarily using relationship quality and shared social contexts, supported by demographic similarity and social visibility. We identify two distinct social cognitive mechanisms: serial order recall, governing initial access and thus explaining why certain individuals are recalled first or early in the process, and association recall, governing the sequential transition and thus explaining why certain individuals are recalled one after another.

Our analysis reveals that distinct social cognitive mechanisms govern these processes. Regarding similarity (H1), gender similarity shows to be important in both serial order and association recall, consistent with established literature on homophily^25^. Shared social contexts (H2 and H3)—including free time, study, traveling, and group membership—Mexhibit a multi-dimensional but unstable role in serial order recall, yet a pronounced influence on association recall. This supports the hypothesis that shared social foci guide the recall and that it might be conceptually connected to a higher social proximity^13,17,32,45^. Relationship quality (H4) emerges as the most robust predictor; the serial order recall tendencies align with strong-tie biases in name generators^35,46^, while positive association recall tendencies suggest that emotional salience and support, not only from ego to alter but also between alters, are key organizational principles governing alter accessibility. Social visibility, conceptualized as older acquaintanceship and perceived popularity (H5 & H6), consistently predicts serial order^47^, but its instability in association recall implies it functions primarily as a cue for first and early recall. Similarity in names was another mechanism (cognitive or methodologically induced) for which we find clear evidence (H7). The recall patterns also appear largely similar but not identical across the two network recall tasks (H8).

Our multivariate statistical approach allows us to simultaneously test all hypothesized mechanisms within the same empirical context, thus facilitating a marginal interpretation of recall patterns. The model distinction between first choices and subsequent choices further shows that the importance of these mechanisms is not constant at different stages of the recall process, but that individuals generally use different cues and mechanisms. Our results thereby connect to previous empirical insights and theoretical arguments. They support a hierarchical recall processing driven by aspects of a latent social proximity dimension^10,17^, represented primarily by relationship quality and shared contexts. Due to the primary focus on relationship quality and shared social contexts in our study, the interplay of mechanisms seems to facilitate the recall of structures that are associated with cliques and strongly connected triads, which is consistent with Brashears and Quintane^48^. The complex interplay between mechanisms can be indirectly inferred from the importance of the predictors in the models, their general tendencies in our robustness checks, and from previous findings in the literature^10,17^: First, the initial nomination is governed by strong ego-alter ties: the ego recalls individuals fitting the “best friend” concept—characterized by high relationship quality, multiple shared contexts, knowing the person longer, higher popularity, and gender homophily (H1A–H6A). Second, we find a stable dual process in which the main driving recall mechanism shows a shift from a pure serial order recall, as the first nomination has no association recall, to more association recall in the subsequent choices where the size of coefficients suggests a large relative importance in choices of alters. As serial order tendencies are weaker in subsequent choices in general, and specifically for serial order recall based on relationship quality^35,45^ and different social contexts, the ego shows the usage of the current alter as a cue for the next, prioritizing strong relationship quality and shared social contexts between the recalled alters, even if those specific attributes diverge from the ego’s own (H1B–H4B). While secondary mechanisms such as gender similarity and social visibility persist, they appear to function as supplementary filters within this dominant trajectory.

Post-hoc analyses suggest additional patterns of relative importance of the mechanisms, depending on the length of the sequence, the maturity of the network itself and the type of social relationship. Mechanisms relying on complex structural information—such as alter-alter relationship quality or social groups—seem to become increasingly important as the network matures across time points. This suggests a cognitive adaptation period in which individuals require time to encode, interpret, and organize latent structural cues, such as alter-alter relations^49,50^. Consequently, earlier time points may rely more on direct ego-alter cues, while complex alter-alter relations peak later. These findings are based on additional temporal analyses (e.g., the interaction of social support (AR) with later waves in Supplementary Fig. 15.8 or the interaction of group membership (AR) with the waves following time point 1 in Fig. 16.4) and align with the latent concept of social proximity^17^.

Comparing our two different relation types, we can also observe that the cognitive framework underlying social recall remains largely consistent across both relationship types (friendship vs. positive interaction), aligning with theories of consistent but content-sensitive memory recall^20,51^. The primary distinction between the two (see Supplementary for a general overview) lay in a weak differential weighting: friendship relations placed greater emphasis on serial order recall, potentially reflecting the stronger, more personal nature of these ties. In contrast, positive interaction exhibited a diminished reliance on serial order recall in favor of association recall, suggesting a process wherein alters are recalled via shared associations to specific contexts. This shift aligns with the theoretical distinctions between these relation types. While friendship is characterized by mutual affect and normative expectations between ego and alter^43^ —suggesting a focus on serial order recall, as it is primarily defined by the specific dyad—positive interactions are often more varied, less clearly defined, and based on specific social contexts. Consequently, these relations appear to be embedded often within situational contexts or collective activities that facilitate the sequential recall of multiple individuals that shared the context or activity, thereby making associative recall more relevant than serial order recall.

The present study combines theoretical concepts from cognitive science and social network science. By leveraging a complete network design that captures both the ego and alter perspectives, we aim to operationalise the often-abstract concept of social proximity and to decompose the important social structural components that are used for the social recall of alters. We demonstrate that specific network motifs — particularly multiplex relationships, alter-alter relations, and social contexts such as cliques—serve as essential cues for cognitive mechanisms. This suggests that structural features defined by relationship quality and shared contexts are not merely network properties but beyond that also active drivers of the recall process. Furthermore, the intersection of social network theory and recall processes is most evident in the observed temporal dynamics. Our explorative findings indicate that as a social network matures, individuals adapt the cognitive mechanisms they use, increasingly incorporating complex structural cues to facilitate recall. This points to a co-evolution of social experience and cognitive processes for the social recall: as the social structure emerges and stabilizes, the memory system effectively perceives and uses these complexities.

Our sequence-focused approach also extends social network science beyond traditional static analyses by revealing the micro-temporal processes underlying network recall. Unlike approaches limited to the set of nominations—specifically, who is nominated versus who is omitted^36^—our findings demonstrate that the recall order itself is systematic. Structural social network features such as homophily, multiplexity, and clustering function not merely as organizational principles for tie formation but as active cognitive mechanisms guiding recall. Importantly, the evidence for similarity, tie overlap and clustering mechanisms in the recall process is detected by conditioning on the observed set of alters. Therefore, they reflect an organizational logic that extends beyond basic network structure. Instead of focusing on who is nominated—which is largely dictated by the network’s structure—this approach examines the timing of nominations. Specifically, it looks at the order in which a person is mentioned once a specific peer has already been named. This suggests that structural network patterns can to some degree be interpreted as the result of how individuals cognitively prioritize certain relationships when navigating their social environment.

To some degree these patterns may thus reflect a level of “hidden” homophily, multiplexity, and clustering in social networks that remains elusive when only network structures are studied. For example, while there may be evidence for such mechanisms in the overall set of recalled alters, it may exist to a stronger degree in ties that are recalled earlier or after each other. Structural network features and cognitive recall could potentially interact and strengthen each other in a complex manner, where “social preferences” for specific types of relationships (e.g., homophilous ties) may make those more cognitively accessible and vice versa. This interaction implies a recursive relationship: cognitive constraints shape the utility of the social environment, while evolving social structures force cognitive adaptation^11^. Consequently, social network structures should be interpreted as a synthesis of social-structural realities and the cognitive mechanisms used to navigate and recall them. While the former is widely studied, the latter part of the interaction has received significantly less attention^39,52^.

The cognitive insights presented in this work also offer pathways for improving social network data collection methodology. By integrating sequential recall dynamics into survey design, researchers can engineer more effective name generators. For instance, employing targeted anchoring cues or procedural prompts can trigger association recall, thereby systematically targeting under-reported subgroups or structurally weak ties^53,54^. Such methodological refinements would not only enhance data completeness but also enable the proactive mitigation of specific recall biases in pilot studies, thereby enabling a more accurate assessment of omission risks.

The ecological empirical setting presented posed some specific analytical challenges, one of which is that the lengths of the recall sequences are relatively short and largely differ between individuals. Our data are thus different from previous studies on exhaustive memory recall and social recall across all of an individual’s social contexts^17,23,51,55^. The resulting structural heterogeneity precludes the direct comparison of absolute parameter magnitudes across models and complicates the application of standard validation techniques, such as posterior predictive checks, thereby limiting the assessability of model performance beyond those presented. Additionally, the used model approach simplifies the sequence generation process by implicitly assuming that only the information from the immediately previous nomination is relevant. Despite the fact that the approach exhibits a slight overweighting of longer sequences due to their increased number of observational steps, this is mitigated by the overall dataset’s emphasis on shorter sequences and our subsequent robustness analyses. However, the potential of higher-order dependencies besides only the immediately previous nomination stays a limitation and should be tackled in future research, focusing especially on the context of social relation recall on more complex network motifs such as triplets or larger social groups.

Social recall is a complex and challenging cognitive task. Our findings demonstrate that social recall incorporates a variety of social cues that are derived from demographic similarities, shared social contexts, relationship qualities, and social visibilities that characterize the (relative) social and structural position of the ego and the recalled alters. These mechanisms operate simultaneously, and individuals switch between mechanisms within the same recall process, using both serial recall cues (based on the ego itself) and association cues (based on previously recalled alters). While general memory recall and complex social network structures have been widely studied by cognitive scientists and network scientists, respectively, their intersection—understanding the cognitive mechanisms in the recall of social ties, especially in an ecological context — is still relatively underexplored^17,48,56^. This is somewhat surprising: Only by efficiently recalling with whom individuals are socially connected can they utilize the widely discussed benefits and power of their social networks^1–6^. This study combined state-of-the-art social network data, statistical methods, and theoretical insights from cognitive science and social network science in an attempt to generate robust and ecologically valid insights into social recall processes. We hope that this study inspires future work at the intersection of social networks and memory.

## Methods

Data collection for this study was approved by the Ethics Board of ETH Zurich (Approval Number 2016-N-27) and was carried out in accordance with all relevant guidelines and regulations. Informed consent was obtained from all subjects prior to data collection.

### Pre-registration of hypotheses

This study was pre-registered on OSF^44^; deviations are detailed in Supplementary Table 1. The most substantial changes involved excluding frequency of interaction and homophily based on the regional canton as predictors due to a large amount of systematic missing values that could not be adequately handled. Furthermore, the analysis focused exclusively on choice models because the pre-registered alternative using the memory-specific associative memory framework^17,57^ failed to converge. This non-convergence is likely attributable to the sequences being considerably shorter than those typically employed in that specific modeling framework^17,58^.

### Participants and procedure

Data was taken from the Swiss Student Life project^42^, which tracked three cohorts of bachelor students (enrolled in 2016 and 2017) at a Swiss technical university. Participants were surveyed online every two to four months over 12 to 14 measurement points (Cohort 1: 14 time points, Cohort 2: 12 time points, Cohort 3: 12 time points), collecting data via online surveys on personal characteristics and social relationships within their cohort (e.g., demographics, perceived social support, studying together, friendship). Data collection adhered to the ethical guidelines of the Swiss Federal Institute of Technology Zurich. For more detailed information about the dataset, see Vörös et al.^42^.

The analysis utilized data from all three cohorts and from all main survey waves ($N_{\textit{ego}}$ = 820 individuals, $N_\textit{community}$ = 1,147 individuals, 12–14 waves of longitudinal network data). To ensure variable completeness across time, specific late measurement points were excluded as they did not collect all relevant variables: time point 14 from Cohort 1, and time points 11 and 12 from Cohorts 2 and 3. Furthermore, any participants who did not act as an ego (i.e., did not provide nominations) at a given time point, or whose response sequences failed the exclusion criteria, described in Section "Response sequences", were removed. For the choice models, we excluded choice instances in which all alternative alters had identical values across all predictors. This exclusion accounted for less than 10% of all choices and was necessary to prevent issues during model comparison calculations. The final participant count per cohort and time point used in the analysis is detailed in Supplementary Section 2.

### Measures

All measures were aggregated for both types of relations in the same way. The following section describes the measures used in this study.

#### Response sequences

The primary dependent variable is the response sequence, defined as the order in which participants nominated alters using a free recall name generator; up to 20 nominations per relation type were possible. At all measurement points, participants first reported their positive interactions and then their friendship relations within the cohort. To aid retrieval, the survey provided a candidate list based on typed letters after a short time period of non-typing—a methodological influence controlled for by the inclusion of the *Name Overlap* predictor (see the Section "Name overlap").

Sequences were first prepared by removing skipped lines, self-nominations, and duplicate nominations of the same alter. Sequences containing fewer than two final nominations were excluded from the analysis as no valid serial order choice could be formed. The final count of usable sequences per cohort and time point is detailed in Supplementary Section 2. A broader overview of the usable choices and persons per cohort and model step are presented in Table 2.

**Fig. 3:**
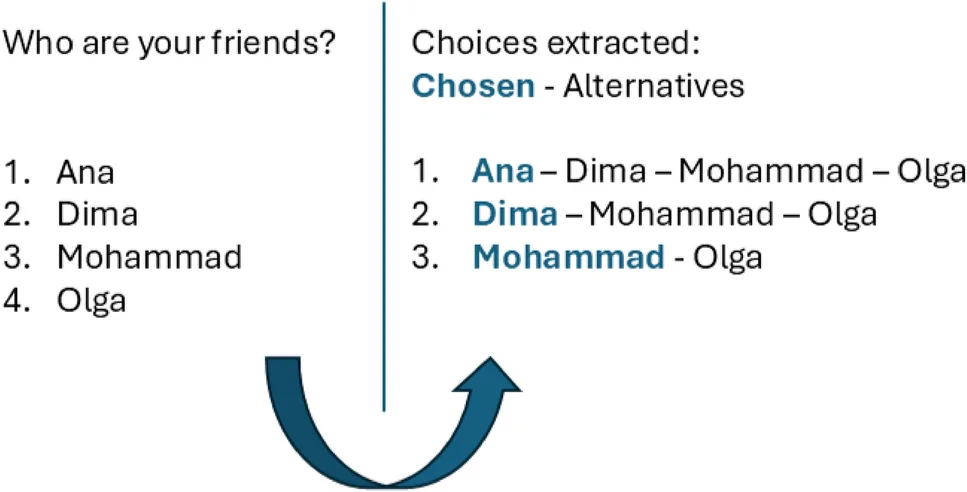
Visualization of the methodological transformation of sequences (left) into separate choices (right).

For the stepwise choice models, the sequences were transformed into a long format, representing the recall process as a series of serial order choices. At each step, the participant (ego) chooses one alter from the set of available candidates (alters not yet nominated in that sequence). For example, if the sequence is Ana, Dima, Mohammad, Olga: In the initial choice ego chooses Ana given the possible alternatives (Dima, Mohammad, Olga). In the subsequent nomination, Ana is no longer eligible, so the ego chooses Dima given the alternatives (Mohammad, Olga). The final choice in the sequence (the last step) is always dropped, as the choice is deterministic (i.e., only Olga is available). This structure allows us to model the probability of selecting the nominated alter against all available, unnominated alters at that specific sequence position (see Fig. 3).

#### Demographics

Participant demographics, including gender, nationality (birth country), and mother tongue, were primarily assessed via self-report. Due to their time-consistent nature, these variables were aggregated across measurement time points by taking the most frequently reported value for each participant (relevant for 7 participants in gender, 84 in nationality; mother tongue was only collected once, so no further information can be used). Where self-reports were missing, data from university administration records is used to fill gaps. In total, for gender no information was available for 45 participants (Cohort 1: 27, Cohort 2: 9, Cohort 3: 9), for nationality no information was available for 57 participants (Cohort 1: 39, Cohort 2: 9, Cohort 3: 9), for mother tongue, no information was available for 426 participants (Cohort 1: 77, Cohort 2: 105, Cohort 3: 244). To create meaningful categories for similarity mechanisms, both nationality and mother tongue were merged based on shared cultural backgrounds (e.g., Cantonese and Mandarin, or subregions within the same country). This standardization ensures that similarities captured genuine cultural or linguistic alignment rather than detailed geographical or dialectical differences. The final demographic distribution is presented in Table 2.

**Table 2:** Distribution of overall participants and their demographics by Cohort.

Demographic	Cohort 1	Cohort 2	Cohort 3
Amount of Persons$_{\textit{Friendship - Initial Choice}}$	175	171	474
Amount of Choices$_{\textit{Friendship - Initial Choice}}$	999	865	2,212
Amount of Persons$_{\textit{Positive Interaction - Initial Choice}}$	194	190	504
Amount of Choices$_{\textit{Positive Interaction - Initial Choice}}$	1,206	1,026	2,431
Amount of Persons$_{\textit{Friendship - Subsequent Choice}}$	163	153	442
Amount of Choices$_{\textit{Friendship - Subsequent Choice}}$	2,933	2,487	6,394
Amount of Persons$_{\textit{Positive Interaction - Subsequent Choice}}$	188	182	487
Amount of Choices$_{\textit{Positive Interaction - Subsequent Choice}}$	7,892	6,903	12,079
Female (%)	35.2	33.8	12.4
Swiss Nationality (%)	59.0	70.5	75.2
Number of distinct Nationalities	27	18	42
Number of distinct Mother Tongues	23	21	36

The presented demographics of the participants were calculated on the processed data, before the exclusion of the egos due to their response sequence, thereby enabling the representation of the starting demographics of the egos and alters in our sample. For a general overview of the sample size throughout the analysis, see Supplementary Section 2

#### Relationship characteristics

We derive the key characteristics of the ego-alter relationship primarily from other free-recall name generators collected within the same survey. To infer the amount of social support, we aggregated the responses of three different additional name generators evaluating three different types of social support: emotional, instrumental, and informational. Variables representing perceived popularity, shared free time, studying, traveling, and pre-existence of the relationship (knowing before the study started) were all operationalised as binary indicators based on nominations in their respective name generators. Perceived group membership was derived from a specific question asking participants to list up to five self-reported social groups—without further specifications and both types of groups: they are part of and groups they are not part of in the cohort—and then nominate cohort members within those groups. These nominations were used to create a variable indicating whether the nominated person is perceived as a member of a self-reported group in the cohort. As a deviation from the preregistration, we excluded the frequency of interactions inferred from online and offline group interactions from the main analysis due to high or systematic missingness, reporting it only in exploratory robustness checks.

#### Name overlap

To control for the methodological influence of the survey’s candidate suggestion list, we introduced the name overlap variable. This binary predictor was coded as one if the nominated alter’s full name (including first, middle, or last names) was identical to any part of another alter’s name within the cohort, and zero otherwise. We utilized this strict criterion as it represents the most likely mechanism through which the suggestion list would influence participant selection. As a sensitivity analysis, a continuous Name Overlap score was also generated. This alternative measure quantified the degree of potential confusion by assessing the fraction of name parts (e.g., first, last name) that overlapped with at least two characters-including one vowel (a, e, i, o, u, ä, ö, ü) of any other name in the cohort. This continuous variable was calculated on the basis of the number of name parts that overlapped with other names, ranging from zero (no overlap) to one (maximal overlap). This alternative definition was used to test the robustness of the primary binary operationalization.

### Response sequence: descriptive statistics

In total, the response sequences comprise longitudinal nominations collected over a maximum of 13 time points. The friendship analysis includes 4076 individual sequences (totaling 15,890 nominations) from 820 individuals, who contributed analyzable sequences across a mean of 4.99 time points (*SD* = 3.08; Median = 5; Range = 1–13). These sequences averaged 4.90 nominations in length (*SD* = 2.56; Median = 4; Range = 2–20). The positive interaction data include 4,663 sequences (31,537 nominations) from 888 individuals, contributing sequences across a mean of 5.25 time points (*SD* = 3.22; Median = 5; Range = 1–13). These sequences were considerably longer, averaging 7.76 nominations (*SD* = 4.28; Median = 7; Range = 2–20).

We assessed the stability of nominations by examining retention rates and changes between consecutive time points. A nominated friend of ego reappeared in 61.76% of ego’s sequences (*SD* = 22.60%; Median = 55.88%; Range = 20.63%–100%), indicating a moderate level of stability. The mean median number of changes (additions and deletions) between time points was 2.84 (*SD* = 2.07; Median = 2.50; Range = 0–15). In contrast, positive interaction nominations were less stable. Positive interaction partners of ego from one survey reappeared in 55.90% of the other surveys (*SD* = 22.54%; Median = 50.00%; Range = 22.12%–100%). There was also a higher number of changes between consecutive survey answers (*Mean* = 5.02, *SD* = 3.37; Median = 5.00; Range = 0–20). This contrast shows more temporal changes for positive interaction relations than for friendship relations.

### Analytical approach

The analysis proceeded through multiple sequential steps. Following data preprocessing, cleaning, and the construction of hypothesized dyadic variables, the sequences were analyzed using two complementary representations. First, the sequence representation was used for descriptive analysis and for calculating the adjusted ratio of clustering (ARC) reported in the Supplementary. Second, the sequences were transformed into a repeated discrete choice scenario (long format). This choice representation formed the basis for the primary analytical method, the conditional logit models.

#### Ego-alter related characteristics

The ego-alter characteristics represent the relationship between the nominating participant (ego) and each of the nominated persons (alter) at every step of the sequence. We consistently used the ego’s perception of the specific alter as the primary data source, supplementing it in the case of relationship quality, where possible and needed, with the reversed information from the alter to the ego as a secondary indicator, assuming that this is at least a useful indicator of the specific characteristic searched. The relationship quality variable, representing perceived social support, was operationalised as the count of social support dimensions (emotional, instrumental, and informational) in which the ego nominated the alter, yielding a range from zero to three.

Missing entries were coded if all support scale nominations were missing. The values were then transformed to represent the maximum observed relationship quality for each specific choice, maintaining a similar value range across all predictors. For social context (shared free time, traveling, and studying together), perceived popularity, and pre-existence of the relationship (knowing before), we used the ego-to-alter nominations of the specific name generator as binary indicators (one = present, zero = absent). We emphasize that perceived popularity reflects the ego’s individual perception of the alter’s standing and is not a cohort-wide consensus measure. Perceived Group Membership was derived as a binary indicator reflecting whether the alter was nominated by the ego as a member of any self-reported group within the cohort, without differentiating the specific groups. For similarity across the major demographic dimensions (gender, nationality, mother tongue), binary homophily indicators were coded as one if the ego and the alter shared at least one value within that category, and zero otherwise.

#### Alter-alter related characteristics

The alter-alter characteristics model the relationship between the previously nominated alter and the subsequently nominated alter at each step of the sequence. This set of predictors captures the association mechanism. We utilized the perception of the previously nominated alter toward the subsequently nominated alter as the primary source of information, supplementing it with the reversed perception where available and needed; assuming that this is at least a useful indicator of the specific characteristic searched. Notably, alter-alter characteristics are not defined for the first-nominated person in any sequence, as no previous nomination exists. For relationship quality, the same zero-to-three count principles were applied as in the ego-alter set, but using nominations between the two alters. The demographic similarity dimensions (gender, nationality, mother tongue) were coded as binary homophily indicators based on whether the two alters shared a characteristic. The key differences appear in the variables: social contexts (free time, studying, traveling, perceived group membership), perceived popularity, and name overlap. The social context variables in the alter-alter version represent whether the alter and the other alters share a social context in the ego perspective (i.e., both travel with the ego or both are members of the same perceived group), representing a structural link perceived by the ego. The perceived popularity variable represents whether the alters are both nominated by the individual as popular. The name overlap variable, which controls for methodological artifacts, is only included in the alter-alter set.

#### Analysis

Data analysis characterized the nomination sequences using a combination of direct and transformed approaches. After the first descriptive analysis of the response sequences—including sequence length and stability—the response sequences were initially analysed using transition matrices and the adjusted clustering ratio^59^ (ARC). As these initial findings are not presented in the main text, their detailed methodology and results are reserved for the Supplementary (see Supplementary Section 4 and 5).

For the main analysis, we transformed the nomination sequences into a repeated discrete choice scenario (see Fig. 3). This structure was analyzed using conditional choice models^60^, specifically employing a Bayesian implementation of a hierarchical conditional discrete choice model^61^ with varying choice sets, as implemented by Uzaheta et al.^62^ in Stan^63^. While acknowledging that the underlying assumption that the potential choice options A,B,C,D are available to the respondent—even if unobserved—may be an oversimplification, this framework allows us to identify the potential driving forces in sequence creation and assess the overrepresentation of choices based on social cues relative to a random selection process. For a detailed description of the choice modeling, see Supplementary Section 6. The analysis started with a fixed-effects base model, which was systematically extended into various configurations described in Section "Robustness".

Model estimation utilized the HMC algorithm with 4000 post-warm-up draws from four chains, following 2000 warm-up iterations. Convergence was assessed using standard diagnostics, using diagnostic plots (e.g., trace plots) and statistics (e.g., rank-normalized split R statistic, effective sample size) as described in Vehtari et al.^64^. Model fit was evaluated via leave-one-out (LOO) cross-validation^65^. We opted not to use standard prior or posterior predictive checks, as established methods are not directly applicable due to the complex model structure and varying choice sets. Prior robustness was shown by running the analysis with different prior specifications. For posterior checks, the varying length and composition of the choice sets prevent a broader interpretation of accuracy and stability metrics. Moreover, the distribution of the chosen values in the simulated data did not provide a clear interpretation, as the observed chosen value is always the next person in the sequence, meaning the first option in the choice set.

A critical consideration is the interpretation of parameter magnitude. Due to the varying choice sets, the coefficient size is dependent on the choice set length and reflects only the relative importance of an attribute within that specific choice set, not its absolute value. Direct comparison of absolute parameter magnitudes across different choice sets is therefore misleading. Consequently, interpretations focus only on the general tendency (sign) and the relative importance determined by comparing the ratio of magnitudes among covariates with comparable ranges, which is the case for all covariates in this study. Parameters are presented on a log-probability scale; a positive (negative) value suggests that the nominated alter is preferred (avoided) over alternatives if the corresponding statistic increases by one unit.

All reported results are based on 90% equal-tailed credibility intervals. All analyses were performed in R^66^ using the following core packages: tidyverse^67^, MemoryOrg^68^, posterior^69^, loo^70^, ggplot^71^, and cmdstanr^72^.

#### Robustness

Due to the complex data structure and variable missingness, we employed a wide range of model configurations and operational decisions to rigorously test the stability of our findings. The most relevant aspects addressed were time, sequence position, sequence lengths, individual differences as a multi-level structure through the repeated measurement scenario, and missing data management. To formally evaluate the model differences, we calculated and compared the model fit for all configurations using leave-one-out cross-validation (LOO)^65^. Because our model building relied on a forward selection approach and a comparison across multiple different model configurations, we included a correction for potential selection-induced bias in the LOO comparison^73^.

We addressed sequence-related heterogeneity by dividing the choice set into two distinct models: one representing initial nominations and one representing subsequent nominations. This division accounts for two factors: first, the lack of a preceding nomination (making AR hypotheses undefinable), and second, the categorical difference in search, in which the initial nomination is potentially more susceptible to unobserved external factors or recency effects. Therefore, the initial nomination in the recall task can be seen as a starting point for the search path, where the starting point itself is of less interest than the path taken from it.

To capture within-sequence dynamics, we estimated models including interaction terms between all predictors and the position of the choice in the sequence (option A: indicator if second or third choice or a later one; option B: indicator if second or third, fourth, fifth or sixth, or a later one) and the centered logarithmic sequence length, to address heterogeneity based on different sequence lengths. To handle temporal heterogeneity, the base model included interaction terms for each predictor and the categorical time point of choice. Time points were clustered according to the students’ semester structure (e.g., first semester: T1–T3; second semester: T4-T6; post–year: T7+). An alternative categorization specifically isolated the first time point to test for unique effects early in the cohort’s formation, before relationships were well established. Individual heterogeneity and heterogeneity related to the repeated measurement scenario were examined through a hierarchical random-effects extension of the base model, incorporating random slopes for all predictors separately.

The approach to missing data involved both inference and structural utilization of the conditional choice model’s properties. We first reduced missingness by inferring reasonable values from available ego and alter data. For choices in which one or more predictors were completely missing across all alternatives, we substituted the missing values with a dummy value. This utilized the property of the conditional choice model to infer parameters based on differences, meaning that when all choice alternatives share the same covariate value, the parameter estimate is unaffected, allowing the choice to remain in the dataset without influencing the coefficients. We substituted, therefore, for all choices in which one or more predictors were completely missing for all choice alternatives within this choice, the missing values with a dummy value to include the choice itself in the choice set without having the dummy value influence our estimation, as all choices had the same value for this predictor. This was only done if the whole choice predictor was missing for the specific choice. In addition, we tested three common missing data strategies: choice-wise deletion, deleting every choice in which at least one predictor was missing from the choice set; list-wise deletion, removing only the missing choice alternatives (labeled in this study as the reduced choice set approach); and the multiple imputation via chained equations (MICE) approach^74^. Due to the complex structure of the dataset and its inherent multi-level dependency, which cannot be fully adequately modeled and imputed, we decided to impute only the demographics for the sample data. In this way, we use the simplest level of imputation, which is, in this case, the ego itself, to impute only a small percentage of data to generate a substantial amount of processed dyadic variables and we use the imputation on one of the variables that had the most missing values in our data. This imputation method was selected because of its parsimony and balance between handling our complex data structure and enabling the imputation of at least some missing data. Following the recommendations of Van Buuren^75^ for the MICE imputation, as well as the recommendations of Zhou and Reiter^76^, and the vignette to handle missing data in the R package brms by Bürkner^77^, we created 100 different imputed datasets and combined their results after inspecting the convergence for each model separately. For more details on the imputation and combination process of the results, see Supplementary Section 7.

Finally, we estimated alternative models to test sensitivity to predictor definitions. These models included the dropped frequency of interaction, reciprocal nominations as an alternative relation quality indicator, similarity based on canton as an additional regional dimension for homophily, an aggregated social context measure, and an alternative operationalization of the name overlap predictor (see Section "Name overlap"). For an overview of the differences in the sample size for the different choice models, see Supplementary Section 2.

## Supplementary Information

## Data Availability

The de-identified data for the main choice models employed in this study are accessible to the public at this link: https://osf.io/2v4qa/overview. The complete raw data of the Swiss Student Life Study, which are necessary for full replication of all robustness analyses, are not publicly available but can be obtained for replication purposes. This is due to data privacy concerns, as required by the Institutional Review Board and Swiss Data Protection Policies. Access to the complete data set, including sensitive variables, is granted under specific conditions. Comprehensive information on raw data access is available on the project website, see: here. The code used to reproduce the analysis is publicly available at this link: https://osf.io/2v4qa/overview

## Code availability

The code used to reproduce the analysis is publicly available at Kovacevic and Stadtfeld^78^.

## Declarations

### AI usage

AI usage for creating this manuscript is declared in Supplementary Table 24.
