# Supplementary material for: Cognitive mechanisms underlying the recall of positive relationships in social networks
Source: Sci Rep. 2026 Jun 17;16:27632. doi: 10.1038/s41598-026-57381-w (PMC13538378; doi:10.1038/s41598-026-57381-w)
Supplement: Supplementary file 1 [file 41598_2026_57381_MOESM1_ESM.pdf]

Supplementary – Cognitive mechanisms underlying the recall of positive relationships in social networks

Zoran Kovacevic[1*] **and Christoph Stadtfeld**[1,2]

[1]Social Networks Lab, ETH Zurich, Stampfenbachstr. 69. 109, Zurich, 8092, Switzerland
[2]Corvinus Institute of Advanced Studies, Corvinus University, Budapest, Hungary
[*]zkovacevic@ethz.ch

ABSTRACT

This document provides detailed in-depth information and analyses that complement the methods and results described in the main manuscript. The supplement is intended to offer greater transparency and methodological depth, ensuring reproducibility and traceability of the reported results. Specifically, this supplement contains further information on the deviations from the preregistration, the sample size overview, additional descriptive analysis, the used Bayesian workflow, the MICE imputation, and all detailed model results and model comparisons. All analysis scripts necessary to replicate the results are available in a dedicated repository[1]. In addition to all scripts, processed anonymised data is provided in the repository to reproduce the main findings. Raw data are not published for data protection reasons, but can be requested here.

Contents

1 Deviation from Preregistration 4

2 Sample Overview 5

3 Detailed Results of Main Models - Table 7

4 Transition Matrices 10
- 4.1 Method . . . 10
- 4.2 Results . . . 10
- 4.3 Discussion . . . 10

5 Adjusted Ratio of Clustering - ARC 12
- 5.1 Method . . . 12
- 5.2 Results . . . 12
- 5.3 Discussion . . . 12

6 Bayesian Analysis: Discrete Conditional Choice Modeling 15
- 6.1 Prior Specifications . . . 15
- 6.2 Sampling Configurations . . . 16
- 6.3 Reporting Style . . . 16

7 MICE Imputation 17

8 Glossary - Model Identification 28

9 LOO Results 29

10 LOO Comparison Results without Correction 34

11 LOO Comparison Results with Correction 38

12 Usage of AI 43

13 Initial Choice - Friendship 44
- 13.1 Initial Choice - Friendship - Weaker Prior . . . 44
- 13.2 Initial Choice - Friendship - Regularized Horseshoe Prior (RHS) . . . 45
- 13.3 Initial Choice - Friendship - Time Interaction . . . 46
- 13.4 Initial Choice - Friendship - Time Interaction with Focus on First Wave . . . 47
- 13.5 Initial Choice - Friendship - Sequence Length Interaction . . . 48
- 13.6 Initial Choice - Friendship - Random Slope for Individuals . . . 49
- 13.7 Initial Choice - Friendship - MICE imputation . . . 50
- 13.8 Initial Choice - Friendship - Reduced Choice set . . . 51
- 13.9 Initial Choice - Friendship - Explorative Robustness Operationalisations . . . 52

14 Initial Choice - Positive Interaction 53
- 14.1 Initial Choice - Positive Interaction - Weak Prior . . . 53
- 14.2 Initial Choice - Positive Interaction - Regularized Horseshoe Prior (RHS) . . . 54
- 14.3 Initial Choice - Positive Interaction - Time Interaction . . . 55
- 14.4 Initial Choice - Positive Interaction - Time Interaction with Focus on First Wave . . . 56
- 14.5 Initial Choice - Positive Interaction - Sequence Length Interaction . . . 57
- 14.6 Initial Choice - Positive Interaction - Random Slope for Individuals . . . 58
- 14.7 Initial Choice - Positive Interaction - MICE imputation . . . 59
- 14.8 Initial Choice - Positive Interaction - Reduced Choice set . . . 60
- 14.9 Initial Choice - Positive Interaction - Explorative Robustness Operationalisations . . . 61

15 Subsequent Choices - Friendship 62

15.1 Subsequent Choices - Friendship - Weak Prior . . . 62
15.2 Subsequent Choices - Friendship - Regularized Horseshoe Prior (RHS) . . . 63
15.3 Subsequent Choices - Friendship - Time Interaction . . . 64
15.4 Subsequent Choices - Friendship - Time Interaction with Focus on First Wave . . . 65
15.5 Subsequent Choices - Friendship - Sequence Length Interaction . . . 66
15.6 Subsequent Choices - Friendship - Sequence Position Interactions . . . 67
15.7 Subsequent Choices - Friendship - Sequence Position Interactions with Focus on First 3 Nominations . . . 68
15.8 Subsequent Choices - Friendship - Sequence Position and Time Interactions . . . 69
15.9 Subsequent Choices - Friendship - Sequence Position Interactions with Focus on First 3 Nominations and Time Interactions with Focus on First Wave . . . 70
15.10 Subsequent Choices - Friendship - Random Slope for Individuals . . . 71
15.11 Subsequent Choices - Friendship - MICE imputation . . . 72
15.12 Subsequent Choices - Friendship - Reduced Choice set . . . 73
15.13 Subsequent Choices - Friendship - Explorative Robustness Operationalisations . . . 74

16 Subsequent Choices - Positive Interaction 75
16.1 Subsequent Choices - Positive Interaction - Weak Prior . . . 75
16.2 Subsequent Choices - Positive Interaction - Regularized Horseshoe Prior (RHS) . . . 76
16.3 Subsequent Choices - Positive Interaction - Time Interaction . . . 77
16.4 Subsequent Choices - Positive Interaction - Time Interaction with Focus on First Wave . . . 78
16.5 Subsequent Choices - Positive Interaction - Sequence Length Interaction . . . 79
16.6 Subsequent Choices - Positive Interaction - Sequence Position Interactions . . . 80
16.7 Subsequent Choices - Positive Interaction - Sequence Position Interactions with Focus on First 3 Nominations 81
16.8 Subsequent Choices - Positive Interaction - Sequence Position and Time Interactions . . . 82
16.9 Subsequent Choices - Positive Interaction - Sequence Position Interactions with Focus on First 3 Nominations and Time Interactions with Focus on First Wave . . . 83
16.10 Subsequent Choices - Positive Interaction - Random Slope for Individuals . . . 84
16.11 Subsequent Choices - Positive Interaction - MICE imputation . . . 85
16.12 Subsequent Choices - Positive Interaction - Reduced Choice set . . . 86
16.13 Subsequent Choices - Positive Interaction - Explorative Robustness Operationalisations . . . 87

References 88

1 Deviation from Preregistration

The subsequent table provides a record of all deviations from the study's preregistration[2] after the data were inspected. These modifications pertain specifically to the data processing and analysis steps.

Deviation	Reasoning
Exclusion of the Hypotheses regarding the "Frequency of Interaction" Variable	The variable exhibited excessive missing data, severely limiting usable observations. Furthermore, the complex, two-dimensional nature of the variable (group nomination and frequency indicator for this group) made standard imputation techniques, such as Multivariate Imputation by Chained Equations (MICE), unsuitable without introducing significant bias regarding assumed group structures and assumptions about the tendency for general group reporting under different frequency indicators. (Note: It was retained as a predictor in the explorative robustness checks.)
Exclusion of the Hypotheses regarding the "Canton" Variable	This variable demonstrated systematic missingness, particularly among foreign students, resulting in a specific subselection of choices for analysis. Including it would have led to a substantial reduction in generalizability. (*Note:* It was retained as a predictor in the explorative robustness checks.)
Disentangling the Three Different Social Contexts for H2A	The three distinct social context name generators (studying, free time, and traveling) were analyzed independently. This approach enables both a differentiated assessment of the individual importance of each context and a combined perspective (via summation) to enhance insight into the multi-faceted nature of social context in the study setting.
Disentangling the Three Different Social Contexts for H2B	See reasoning for "Disentangling the Three Different Social Contexts for H2A"
Dropping Individual Time Point Analysis	Individual time point analysis was discontinued due to the rapid decline in usable sequences over time and the decision to prioritize comprehensive, higher-level analyses, including extensive robustness checks for time and sequence heterogeneity.
Exclusion of Search for Associative Memory (SAM) Model	The overall short sequence lengths in the dataset precluded the effective application and convergence of the SAM model parameters, rendering its use unfeasible.
Exclusion of the Latest Waves per Cohort	The final waves for each cohort were systematically excluded because key variables were not consistently collected, ensuring only data points with a comparable and complete set of variables were retained for analysis.
Separate Modeling for Initial and Subsequent Choices	Our study analytically separates initial recall (the first recalled alter) from subsequent recall (all subsequent alters). We consider the first or initial recall (e.g., when trying to remember a person without prior associations or specific cues) categorical, separate from subsequent recall (e.g., when trying to remember a second person whose recall may be aided by the initial recall or a specific cue) in our study. This analytical distinction is grounded in the general understanding of free-recall tasks primarily for two compelling reasons. First, initial recall is based on different experiments, often seen as the most influenced by primacy-recency effects[3]. In our study, which covers episodic memory, this is especially related to the influence of recency and external stimuli on the initial item recalled (for a detailed overview, see Pachur et al.[4]). Neither aspect can be covered in our study. Second, only the initial recall has a clear observable ego-alter relation that can be used as a recall cue, unlike the subsequent recall, which also allows the alter-alter relationship to be used as a cue[4]. In addition, the first recalled item interferes with the recall of the following items. This enables us to understand the mechanisms related to clustering from the second nomination onward, as the initial nomination has no previous nomination to cluster and interfere with. Of course, associations of other concepts may play a role in the initial recall, but this cannot be adequately determined in our study. To adequately address these circumstances, we decided to separate the two recall steps.

2 Sample Overview

The following tables provide a detailed overview of the number of participants and unique choices that could be used after preprocessing and exclusion criteria.

Table 1. Amount of Participants and Unique Choices Across Cohorts and Time Points

**Friendship**<br>**Time Point**	**Cohort 1**	**Cohort 2**	**Cohort 3**
**TP.1**	67/176	56/147	262/681
**TP.2**	123/451	119/486	274/968
**TP.3**	123/497	116/473	296/1,142
**TP.4**	112/425	118/510	323/1,359
**TP.5**	101/450	100/388	257/1,151
**TP.6**	89/365	86/318	184/844
**TP.7**	61/245	60/235	129/517
**TP.8**	63/277	74/295	180/701
**TP.9**	60/252	70/254	157/627
**TP.10**	51/204	66/246	150/616
**TP.11**	46/165	-/-	-/-
**TP.12**	50/204	-/-	-/-
**TP.13**	52/221	-/-	-/-
**TP.14**	-/-	-/-	-/-

**Positive Interaction**<br>**Time Point**	**Cohort 1**	**Cohort 2**	**Cohort 3**
**TP.1**	121/477	105/474	324/1,156
**TP.2**	155/1,167	140/1,112	304/1,708
**TP.3**	149/1,308	132/1,166	327/1,969
**TP.4**	136/1,025	132/1,159	347/2,321
**TP.5**	120/1,033	117/973	273/1,853
**TP.6**	98/775	97/740	200/1,341
**TP.7**	70/546	67/589	138/877
**TP.8**	64/510	86/629	193/1,230
**TP.9**	68/558	74/545	164/1,028
**TP.10**	58/444	76/542	161/1,027
**TP.11**	51/374	-/-	-/-
**TP.12**	56/419	-/-	-/-
**TP.13**	60/462	-/-	-/-
**TP.14**	-/-	-/-	-/-

Note: The first number in the cell represents the number of participants, and the second number represents the number of unique choices (multiple per response sequence) for each time point and cohort.

Table 2. Amount of Final Analyzed Participants and Unique Choices Across all Used Datasets for the Choice Models

Initial Choice Models - Sample Size - Friendship/Positive Interaction

Database	Amount of Persons included	Amount of Choices included
**Base dataset**	655/665	2,196/1,844
**Frequency dataset**	594/476	1,590/889
**Reciprocity dataset**	522/491	1,148/818
**Social Context Aggregated - dataset**	655/664	2,191/1,842
**Swiss Canton dataset**	492/424	1,417/1,020
**Reduced Choice Set - dataset**	782/852	3,417/3,994

Subsequent Choice Models - Sample Size - Friendship/Positive Interaction

Database	Amount of Persons included	Amount of Choices included
**Base dataset**	693/808	6,113/10,700
**Frequency dataset**	618/717	3,057/3,528
**Reciprocity dataset**	619/736	3,444/5,329
**Social Context Aggregated - dataset**	693/808	6,112/10,699
**Swiss Canton dataset**	627/727	3,494/4,987
**Name Alternative 2 - dataset**	695/811	6,156/10,793
**Reduced Choice Set - dataset**	728/842	9,058/20,927

Note: The first number in the cell represents the specific number for friendship relations, and the second number for positive interactions.

3 Detailed Results of Main Models - Table

Table 3. Summary Statistics of the Posterior Distribution, Mean, Standard Deviation (SD), and 90%-Credibility Interval [CI] for the Base Models.

		Initial Recall		Subsequent Recall	
	**Variable**	**Friendship**	**Positive Interaction**	**Friendship**	**Positive Interaction**
	EGO - ALTER				
Similarity	Gender	**0.39 (0.07)** [0.27, 0.51]	**0.23 (0.08)** [0.10, 0.36]	**0.11 (0.04)** [0.04, 0.18]	0.01 (0.03) [-0.04, 0.05]
	Nationality	**0.17 (0.09)** [0.03, 0.32]	0.01 (0.09) [-0.14, 0.17]	0.04 (0.06) [-0.06, 0.14]	-0.00 (0.04) [-0.07, 0.07]
	Mother Tongue	0.02 (0.08) [-0.12, 0.15]	0.12 (0.09) [-0.03, 0.27]	0.04 (0.05) [-0.04, 0.11]	0.02 (0.03) [-0.03, 0.08]
Social Context	Free Time	**0.68 (0.08)** [0.54, 0.82]	**0.85 (0.09)** [0.70, 1.00]	**0.22 (0.07)** [0.10, 0.35]	0.06 (0.05) [-0.02, 0.15]
	Travel	**0.32 (0.08)** [0.18, 0.45]	**0.34 (0.09)** [0.19, 0.48]	0.08 (0.07) [-0.03, 0.20]	0.08 (0.06) [-0.01, 0.18]
	Study	**0.53 (0.08)** [0.41, 0.66]	**0.71 (0.09)** [0.57, 0.85]	0.11 (0.06) [-0.00, 0.21]	0.04 (0.05) [-0.04, 0.12]
	Group Member	**0.30 (0.12)** [0.11, 0.50]	**0.40 (0.12)** [0.21, 0.60]	-0.06 (0.07) [-0.17, 0.06]	0.00 (0.04) [-0.07, 0.07]
Quality	Support	**1.74 (0.09)** [1.59, 1.89]	**1.63 (0.09)** [1.48, 1.78]	**0.67 (0.04)** [0.60, 0.75]	**0.67 (0.04)** [0.61, 0.74]
Visibility	Known Before	**0.33 (0.10)** [0.16, 0.50]	**0.47 (0.11)** [0.28, 0.65]	**0.20 (0.06)** [0.10, 0.30]	**0.15 (0.05)** [0.07, 0.24]
	Popular	**0.39 (0.08)** [0.25, 0.52]	**0.45 (0.10)** [0.29, 0.60]	**0.20 (0.06)** [0.09, 0.30]	**0.17 (0.05)** [0.09, 0.25]
	ALTER - ALTER				
Similarity	Gender	-	-	**0.31 (0.04)** [0.24, 0.38]	**0.18 (0.03)** [0.13, 0.23]
	Nationality	-	-	0.04 (0.06) [-0.05, 0.14]	0.05 (0.04) [-0.02, 0.12]
	Mother Tongue	-	-	0.01 (0.05) [-0.08, 0.10]	0.04 (0.04) [-0.03, 0.10]
Social Context	Free Time	-	-	**0.25 (0.09)** [0.10, 0.40]	**0.60 (0.07)** [0.48, 0.72]
	Travel	-	-	**0.35 (0.13)** [0.14, 0.57]	**0.28 (0.12)** [0.09, 0.48]
	Study	-	-	**0.37 (0.09)** [0.23, 0.51]	**0.47 (0.07)** [0.35, 0.59]
	Group Member	-	-	**0.75 (0.07)** [0.64, 0.87]	**0.99 (0.05)** [0.91, 1.08]
Quality	Support	-	-	**0.82 (0.05)** [0.73, 0.90]	**0.96 (0.04)** [0.90, 1.02]
Visibility	Known Before	-	-	0.39 (0.32) [-0.13, 0.91]	**0.63 (0.22)** [0.25, 1.00]
	Popular	-	-	0.02 (0.14) [-0.21, 0.25]	0.18 (0.12) [-0.02, 0.38]
-	Name Overlap	-	-	**0.68 (0.14)** [0.44, 0.91]	**0.83 (0.10)** [0.68, 0.99]

Note: Mean, standard deviation (SD), and 90%-credibility interval [CI] (q5, q95) are presented. We marked in bold all predictors with a 90%-CI that does not include zero.

Table 4. Summary Statistics of the Posterior Distribution, Mean, Standard Deviation (SD), and 95%-Credibility Interval [CI] for the Base Models.

	Variable	Initial Recall		Subsequent Recall	
		**Friendship**	**Positive Interaction**	**Friendship**	**Positive Interaction**
	EGO - ALTER				
Similarity	Gender	**0.39 (0.07)** [0.25, 0.54]	**0.23 (0.08)** [0.07, 0.38]	**0.11 (0.04)** [0.03, 0.19]	0.01 (0.03) [-0.05, 0.06]
	Nationality	0.17 (0.09) [-0.00, 0.35]	0.01 (0.09) [-0.17, 0.20]	0.04 (0.06) [-0.07, 0.15]	-0.00 (0.04) [-0.08, 0.08]
	Mother Tongue	0.02 (0.08) [-0.14, 0.18]	0.12 (0.09) [-0.06, 0.30]	0.04 (0.05) [-0.05, 0.13]	0.02 (0.03) [-0.04, 0.09]
Social Context	Free Time	**0.68 (0.08)** [0.52, 0.85]	**0.85 (0.09)** [0.67, 1.03]	**0.23 (0.07)** [0.08, 0.37]	0.06 (0.05) [-0.04, 0.17]
	Travel	**0.32 (0.08)** [0.16, 0.48]	**0.34 (0.09)** [0.17, 0.51]	0.08 (0.07) [-0.05, 0.22]	0.08 (0.06) [-0.03, 0.20]
	Study	**0.53 (0.08)** [0.38, 0.68]	**0.71 (0.09)** [0.54, 0.88]	0.11 (0.06) [-0.02, 0.23]	0.04 (0.05) [-0.06, 0.14]
	Group Member	**0.30 (0.12)** [0.08, 0.54]	**0.40 (0.12)** [0.17, 0.64]	-0.06 (0.07) [-0.20, 0.08]	0.00 (0.04) [-0.08, 0.09]
Quality	Support	**1.74 (0.09)** [1.56, 1.92]	**1.63 (0.09)** [1.45, 1.80]	**0.67 (0.04)** [0.59, 0.76]	**0.67 (0.04)** [0.60, 0.75]
Visibility	Known Before	**0.33 (0.10)** [0.13, 0.53]	**0.47 (0.11)** [0.26, 0.69]	**0.20 (0.06)** [0.07, 0.33]	**0.15 (0.05)** [0.06, 0.25]
	Popular	**0.39 (0.08)** [0.22, 0.55]	**0.45 (0.10)** [0.26, 0.64]	**0.20 (0.06)** [0.07, 0.32]	**0.17 (0.05)** [0.07, 0.27]
	ALTER - ALTER				
Similarity	Gender	-	-	**0.31 (0.04)** [0.23, 0.39]	**0.18 (0.03)** [0.13, 0.24]
	Nationality	-	-	0.04 (0.06) [-0.07, 0.16]	0.05 (0.04) [-0.03, 0.13]
	Mother Tongue	-	-	0.01 (0.05) [-0.09, 0.12]	0.04 (0.04) [-0.04, 0.11]
Social Context	Free Time	-	-	**0.25 (0.09)** [0.08, 0.43]	**0.60 (0.07)** [0.45, 0.75]
	Travel	-	-	**0.35 (0.13)** [0.10, 0.61]	**0.28 (0.12)** [0.06, 0.51]
	Study	-	-	**0.37 (0.09)** [0.20, 0.53]	**0.47 (0.07)** [0.33, 0.62]
	Group Member	-	-	**0.75 (0.07)** [0.61, 0.89]	**0.99 (0.05)** [0.89, 1.09]
Quality	Support	-	-	**0.82 (0.05)** [0.72, 0.91]	**0.96 (0.04)** [0.89, 1.04]
Visibility	Known Before	-	-	0.39 (0.32) [-0.23, 1.01]	**0.63 (0.22)** [0.19, 1.07]
	Popular	-	-	0.02 (0.14) [-0.25, 0.30]	0.18 (0.12) [-0.06, 0.42]
-	Name Overlap	-	-	**0.68 (0.14)** [0.40, 0.96]	**0.83 (0.10)** [0.65, 1.02]

Note: Mean, standard deviation (SD), and 95%-credibility interval [CI] (q2.5, q97.5) are presented. We marked in bold all predictors with a 95%-CI that does not include zero.

Table 5. Summary Statistics of the Probability of Direction for the Base Models.

	Variable	Initial Recall Friendship	Initial Recall Positive Interaction	Subsequent Recall Friendship	Subsequent Recall Positive Interaction
	EGO - ALTER				
Similarity	Gender	1.000 / 0.000	0.998 / 0.002	0.997 / 0.003	0.605 / 0.395
	Nationality	0.975 / 0.025	0.548 / 0.452	0.755 / 0.245	0.497 / 0.503
	Mother Tongue	0.583 / 0.417	0.905 / 0.095	0.787 / 0.213	0.764 / 0.236
Social Context	Free Time	1.000 / 0.000	1.000 / 0.000	0.998 / 0.002	0.880 / 0.120
	Travel	1.000 / 0.001	1.000 / 0.001	0.887 / 0.113	0.930 / 0.070
	Study	1.000 / 0.000	1.000 / 0.000	0.947 / 0.053	0.782 / 0.218
	Group Member	0.995 / 0.005	1.000 / 0.001	0.202 / 0.798	0.520 / 0.480
Quality	Support	1.000 / 0.000	1.000 / 0.000	1.000 / 0.000	1.000 / 0.000
Visibility	Known Before	1.000 / 0.001	1.000 / 0.000	0.999 / 0.001	0.999 / 0.001
	Popular	1.000 / 0.000	1.000 / 0.000	0.999 / 0.001	1.000 / 0.001
	ALTER - ALTER				
Similarity	Gender	-	-	1.000 / 0.000	1.000 / 0.000
	Nationality	-	-	0.757 / 0.243	0.888 / 0.112
	Mother Tongue	-	-	0.600 / 0.400	0.842 / 0.158
Social Context	Free Time	-	-	0.998 / 0.002	1.000 / 0.000
	Travel	-	-	0.996 / 0.004	0.994 / 0.006
	Study	-	-	1.000 / 0.001	1.000 / 0.000
	Group Member	-	-	1.000 / 0.000	1.000 / 0.000
Quality	Support	-	-	1.000 / 0.000	1.000 / 0.000
Visibility	Known Before	-	-	0.890 / 0.110	0.998 / 0.002
	Popular	-	-	0.562 / 0.438	0.931 / 0.069
-	Name Overlap	-	-	1.000 / 0.000	1.000 / 0.000

Note: Posterior probabilities are presented as a tuple: the first value indicates the probability of the posterior mass exceeding zero, and the second indicates the probability of the mass falling below zero.

4 Transition Matrices

4.1 Method

We utilized transition matrices to descriptively characterize the emergent sequential patterns of nominations. These matrices illustrate the conditional probabilities of a nomination transitioning between two states: *match* (the specified relation characteristic is fulfilled) and *no match* (the characteristic is not fulfilled). The resulting probabilities describe the degree of state persistence (low-mixture) or fluctuation (high-mixture) within the sequence. For the calculation, individuals with missing values for the specific variable and all sequences lacking any state switch were excluded. The transition matrices for each relationship type are visualized in Fig. 2.

4.2 Results

Inspecting the emerging patterns for the hypothesized variables reveals a relatively stable dynamic; in almost all cases, the probabilities of staying in the given state are higher than those of switching states. Switching states primarily occurs from a *match* to a *no match* state, suggesting that certain established characteristics are not consistently maintained; this trend is particularly evident in alter-alter relations.

For similarity (gender, nationality, mother tongue), matches show a slight tendency to persist, but if not established, the likelihood of establishing one is balanced for both ego-alter and alter-alter ties, suggesting weak state stability.

Social Context exhibits a strong tendency to remain in the *no match* state and a high probability of switching from *match* to *no match*, indicating less stability in the matching state. However, for ego-alter ties, group membership is an exception, maintaining the *match* state more stably than other context characteristics.

Relationship quality displays weak stable state behavior for both relation types (ego-alter and alter-alter), with the stability being weaker for the alter-alter *match* to *match* transition.

Finally, social visibility exhibits a stable dynamic for *no match* states but a dynamic behavior for *match* states. For ego-alter ties, both aspects tend toward the *no match* state, while popularity in alter-alter ties behaves atypically, showing stable repetition of both *match* and *no match* states.

4.3 Discussion

The descriptive transition matrices highlight that relationship quality and, for ego-alter ties, the social context of social groups are the most consistent factors promoting state persistence. In terms of overall dynamics, relationship quality, social visibility, and social context exhibit substantially unbalanced transition probabilities, suggesting a considerable influence on sequence structure. Conversely, similarity appears only weakly informative regarding transition dynamics. The specific interaction (ego-alter vs. alter-alter) and the relation type consistently influence the importance and dynamics observed across all mechanisms.

Figure 1. Transition Matrices for each Step in the Sequences for each Variable. *No Match* means that the specific mechanism is not fulfilled. *Match* means the specific mechanism is fulfilled.

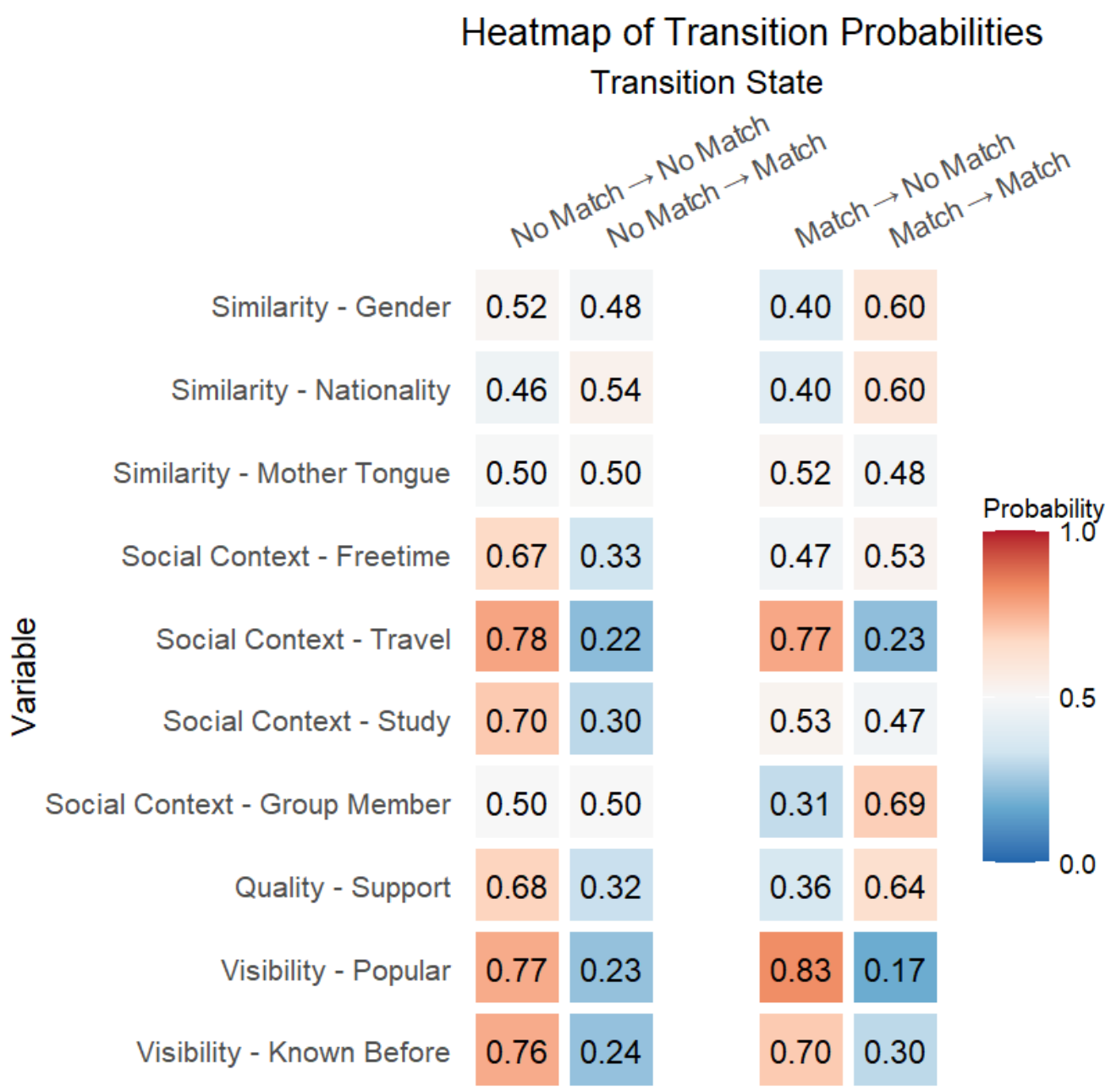

Figure 2. Friendship - EGO ALTER

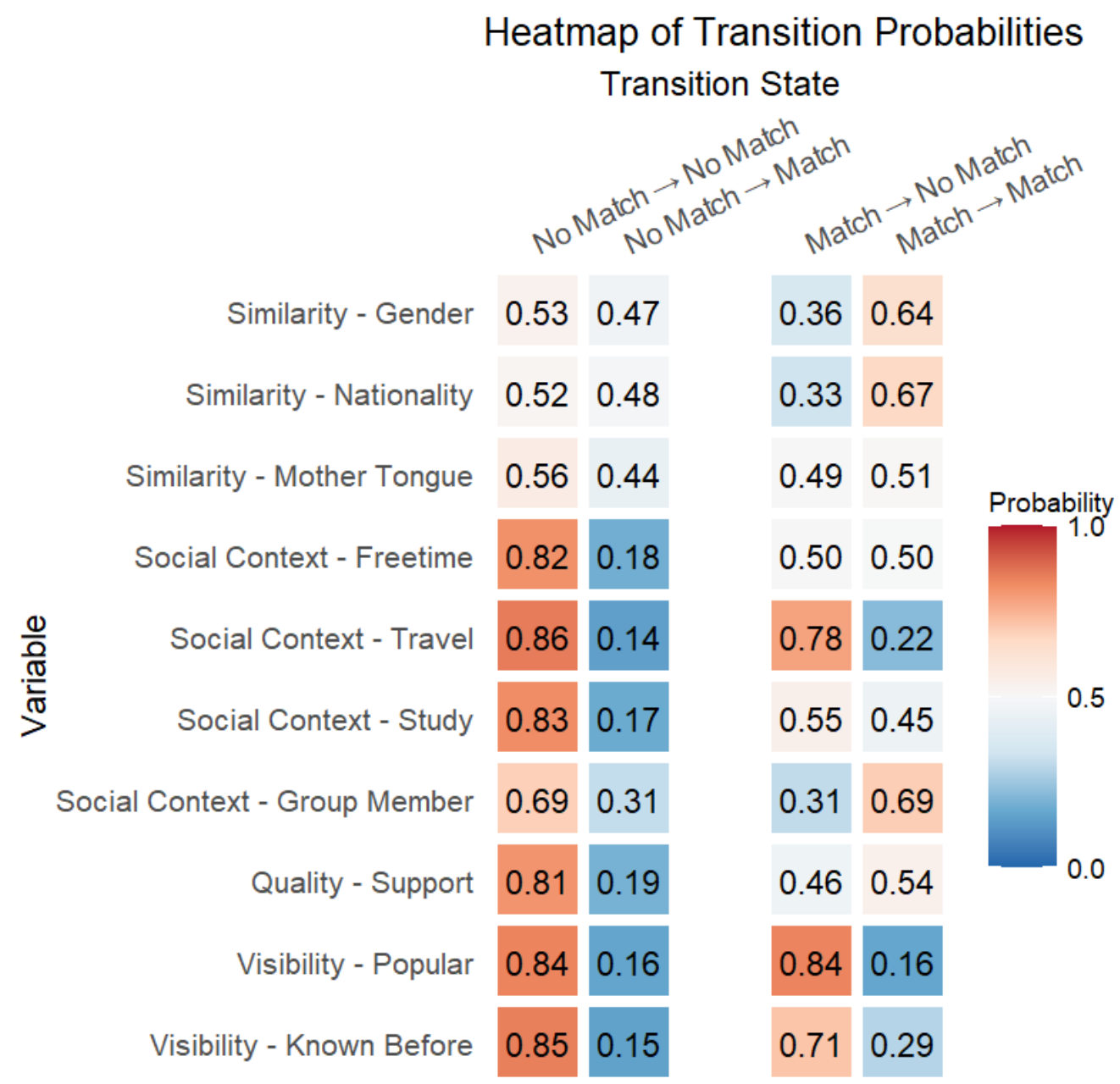

Figure 3. Positive Interaction - EGO ALTER

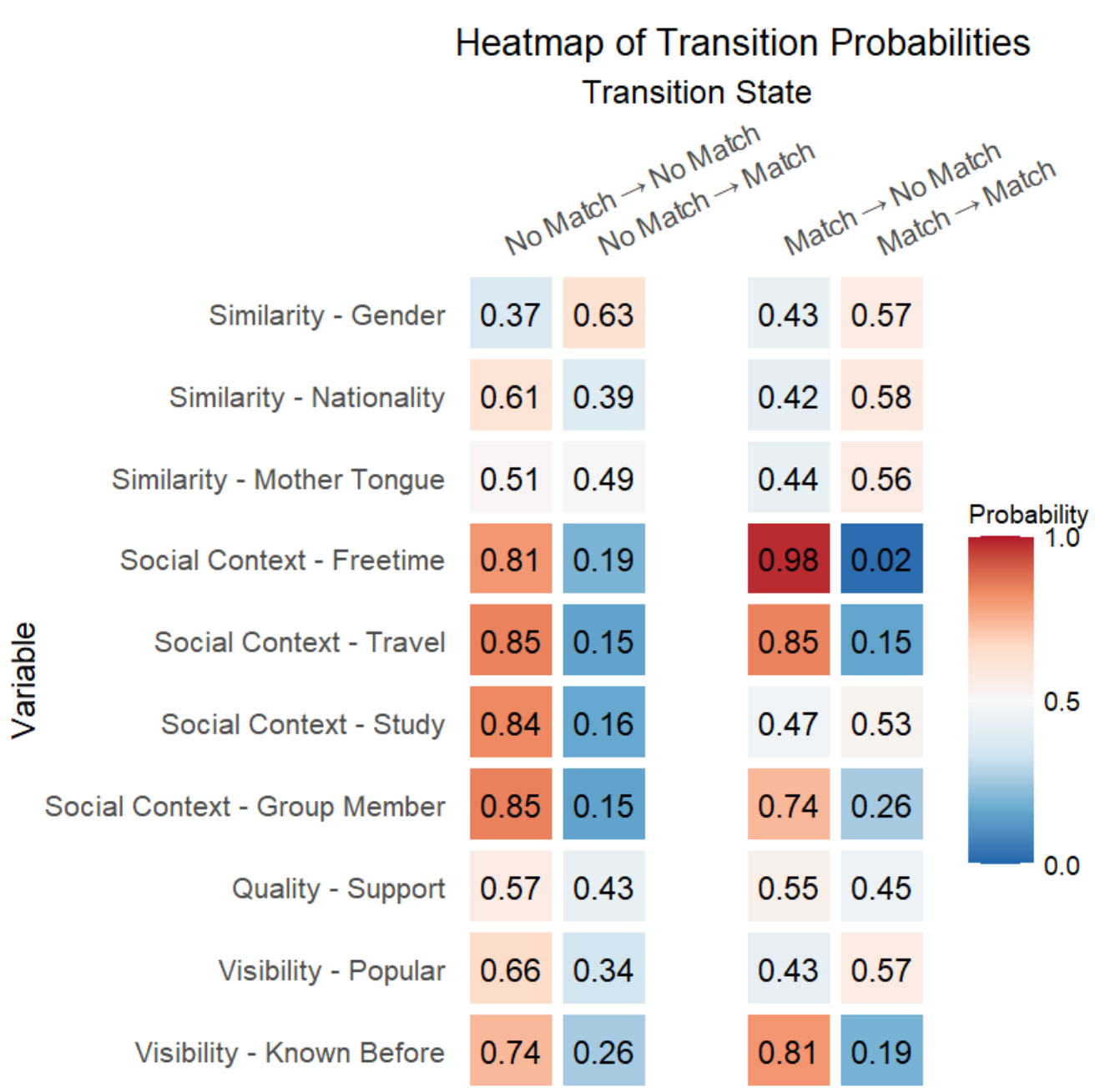

Figure 4. Friendship - ALTER ALTER

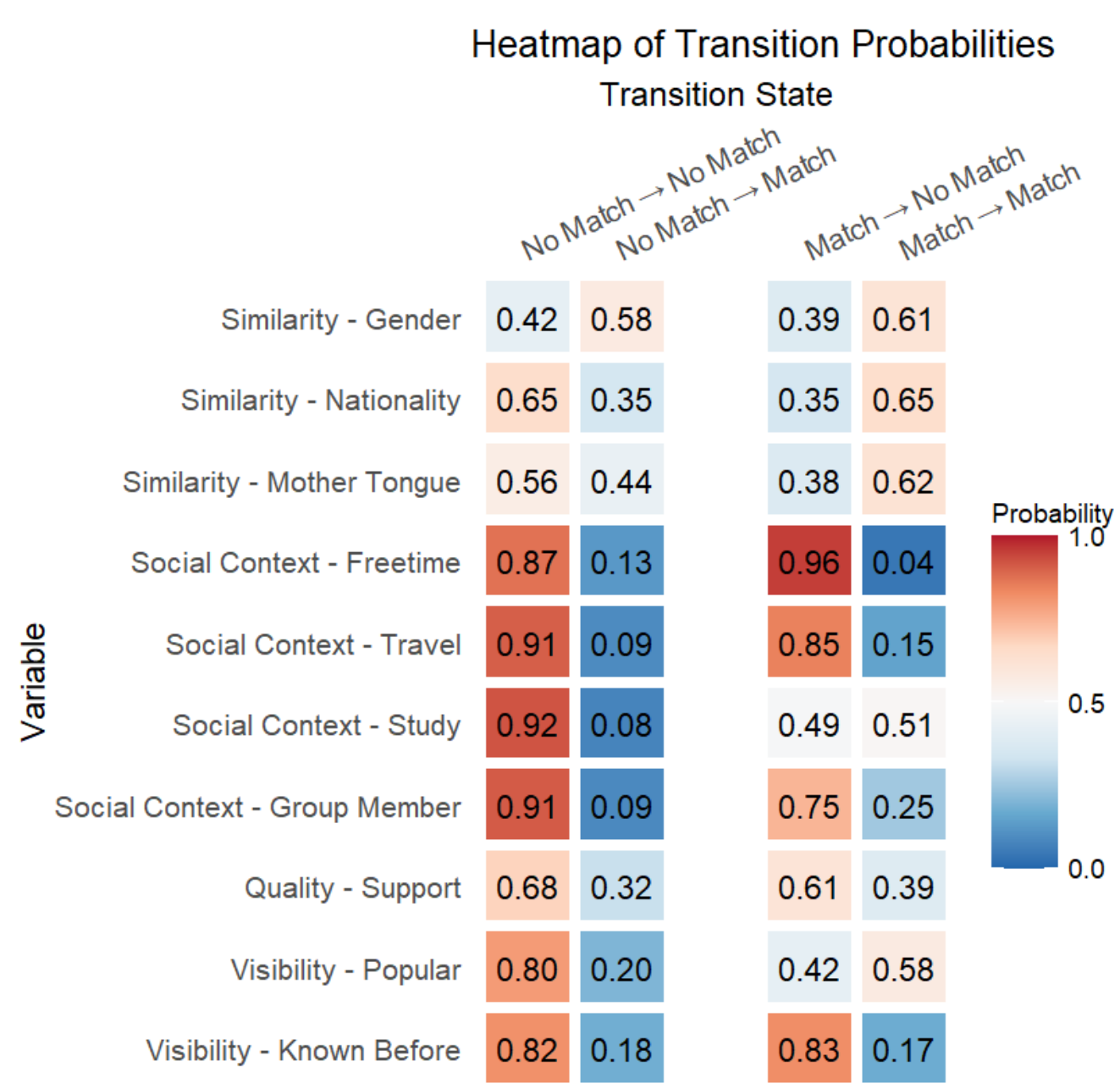

Figure 5. Positive Interaction - ALTER ALTER

5 Adjusted Ratio of Clustering - ARC

5.1 Method

We employed the Adjusted Ratio of Clustering[5] (ARC) to descriptively analyze the tendency of participants to cluster their entire nomination sequence based on specific conditions. ARC is a popular measure of clustering corrected by random chance[6–8]. The concrete formula for the ARC of a sequence for a specific variable is:

$$\text{ARC} = \frac{O-E}{M-E}$$

with:

- O: observed clustering in the sequence
- E: expected clustering at random
- M: maximum potential clustering

For all parameters, clustering is represented as the number of adjacent nominations that share the same value of the specified variable. ARC ranges between zero for a clustering, which is equal to the expected clustering at random, and one if a person produces the maximum clustering, which corresponds to the smallest amount of switches. Due to the relatively short lengths of the sequences, we faced three difficulties: sequences without a switch in category, perfect misalignment, and statistical power. Therefore, we had to modify the existing ARC implementation to drop sequences with only one expression of the variable, which are more likely for shorter sequences, and to fix the minimum of the ARC to the opposite of perfect alignment (perfect misalignment = -1). This was done because short sequences can easily have a sequence pattern that maximizes the number of category switches. This is less of an issue for longer sequences. Due to our modification, the range of the ARC range changed from -1 to 1. It is important to note that minus one indicates the minimum possible value for an anti-clustering pattern in a short sequence. However, in this study, due to the mentioned difficulties, it is treated as an indicator of no substantive clustering/randomness. Additionally, due to the lack of statistical power and the complexity of handling varying sequence lengths, we did not conduct a significance test for the ARCs and omitted direct comparisons of the ARCs with previous research[6,7]. For the only continuous variable, we used a binarized version based on the maximum expression of this variable.

5.2 Results

The modified ARC was used as a descriptive indicator, with positive coefficients (Max = 1) showing higher clustering than expected by chance and negative coefficients (Min = -1) showing avoidance/no indication of clustering. We observed generally similar behavior between friendship and positive interaction, though with significant differences in the importance of specific characteristics.

Relationship quality emerged as the strongest and most context-sensitive clustering factor. For ego-alter ties, support showed the highest clustering across both relations (friendship M = 0.37; positive interaction M = 0.40), indicating a strong tendency to sequence nominations based on the quality of one's own relationship with the alter. However, for alter-alter ties, the importance of support dramatically declined, becoming minor (M = 0.09 for friendship) or even showing no influence (M = -0.04 for positive interaction).

Social context consistently drove substantial clustering regardless of relation type or context. For ego-alter ties, social context variables (group membership, free time, study, travel) showed strong clustering (means around 0.21 to 0.31 for friendship; for positive interaction around 0.16 to 0.37). This strong influence intensified for alter-alter ties, with means ranging from 0.44 to 0.62 in both relations.

Social Visibility exhibited a clear shift in importance depending on the context of the tie and the dimension. In terms of popularity, it played a minor clustering role in ego-alter ties (e.g., popularity: friendship M = 0.09; positive interaction M = 0.04), but became a more substantial clustering factor for alter-alter ties (popularity: friendship M = 0.31; positive interaction M = 0.22). Notably, known before clustered only weakly in ego-alter ties, and showed no clustering for alter-alter relations.

Similarity generally played a weaker role, often yielding ARCs closest to zero. For ego-alter ties, gender showed moderate clustering in friendship (M = 0.20), but clustering was weaker or absent for nationality and mother tongue. For alter-alter ties, Nationality was a clear exception, showing substantial clustering in both relations (friendship M = 0.45; positive interaction M = 0.42), while Gender and Language at least clustered weakly.

5.3 Discussion

The ARC results consistently highlight the central and context-dependent roles of relationship quality and social context for sequence structure. Relationship quality emerged as the most influential factor in determining sequence clustering when

considering ego-alter ties, particularly the level of perceived support an individual experiences with the nominee. However, the importance of support strongly declines when considering alter-alter ties. This drop-off, combined with a significant rise in the clustering effect of popularity among the alters, suggests an interesting potential interaction between relationship quality aspects and visibility aspects. In contrast, social context proved to be a consistently powerful driver. Its influence was already substantial for ego-alter ties and intensified significantly for alter-alter ties across both relation types, positioning shared context as a robust mechanism for non-random clustering. Finally, similarity or known before as the other dimension of social visibility generally play a weaker, more peripheral role compared to the other factors. Nevertheless, nationality stands out as a clear exception, exhibiting substantial clustering for alter-alter ties, suggesting that certain shared background characteristics can exert a powerful homophily effect when considering the shared environment of the alters. Although the standard deviations suggest considerable heterogeneity in individual sequences, the ARCs clearly identify these key, non-random, and context-dependent drivers of the nomination process.

Table 6. ARC - Friendship nomination sequences

Mechanism	Variable	Mean	Median	SD
**EGO-ALTER**				
Similarity	Gender	0.20	0.27	0.71
	Nationality	0.07	0.00	0.72
	Mother Tongue	-0.04	0.00	0.75
Social Context	Free time	0.29	0.38	0.71
	Travel	0.21	0.00	0.76
	Study	0.31	0.40	0.71
	Group Member	0.30	0.40	0.72
Quality	Support $_{\text{binarized}}$	0.37	1.00	0.72
Visibility	Known Before	0.18	0.00	0.76
	Popular	0.09	0.00	0.75
**ALTER-ALTER**				
Similarity	Gender	0.13	0.00	0.72
	Nationality	0.45	0.63	0.63
	Mother Tongue	0.25	0.27	0.71
Social Context	Free time	0.62	1.00	0.57
	Travel	0.44	1.00	0.69
	Study	0.60	1.00	0.59
	Group Member	0.44	1.00	0.66
Quality	Support $_{\text{binarized}}$	0.09	0.00	0.76
Visibility	Known Before	0.05	0.00	0.77
	Popular	0.31	0.46	0.72
-	Name Overlap	0.04	-0.20	0.74

Note: Due to the short sequences used to calculate the ARCs and the related difficulties in the distribution of the ARCs within one variable, no statistical testing was conducted.

Table 7. ARC - Positive Interaction nomination sequences

Mechanism	Variable	Mean	Median	SD
**EGO-ALTER**				
Similarity	Gender	0.12	0.10	0.56
	Nationality	0.05	-0.06	0.58
	Mother Tongue	-0.11	-0.14	0.62
Social Context	Free time	0.36	0.45	0.63
	Travel	0.16	0.00	0.69
	Study	0.36	0.43	0.63
	Group Member	0.37	0.43	0.60
Quality	Support $_{binarized}$	0.40	1.00	0.69
Visibility	Known Before	0.14	0.00	0.71
	Popular	0.04	-0.15	0.66
**ALTER-ALTER**				
Similarity	Gender	0.12	0.05	0.58
	Nationality	0.42	0.46	0.53
	Mother Tongue	0.17	0.17	0.64
Social Context	Free time	0.62	1.00	0.56
	Travel	0.44	1.00	0.68
	Study	0.62	1.00	0.58
	Group Member	0.46	0.59	0.60
Quality	Support $_{binarized}$	-0.04	-0.16	0.72
Visibility	Known Before	-0.14	-0.29	0.75
	Popular	0.22	-0.08	0.70
-	Name Overlap	0.01	-0.20	0.61

Note: Due to the short sequences used to calculate the ARCs and the related difficulties in the distribution of the ARCs within one variable, no statistical testing was conducted.

6 Bayesian Analysis: Discrete Conditional Choice Modeling

A Bayesian hierarchical discrete conditional choice model with a varying choice set was used to analyze the data, following the work of Uzaheta et al.[9]. Implemented in Stan[10], the model assumes that a choice is determined by the utility of its alternatives, which is a linear function of their characteristics. The choices are then modeled with a logit probability distribution.

$$U_{i,t} = x_{i,t}\beta$$
$$P(y_t = j|CS_t) = \frac{\exp(U_{j,t})}{\sum_{k\in CS_t}\exp(U_{k,t})}$$

where:

- y_t: The chosen alternative at time t.
- $x_{i,t}$: A vector of covariates for alternative i at time t.
- β: A vector of coefficients representing preferences.
- $U_{i,t}$: The utility of alternative i at time t.
- CS_t: The set of available alternatives at time t.

6.1 Prior Specifications

For the different model configurations, different priors were used for the coefficients, β, to evaluate their impact on the results or to adapt the estimation to the given model configurations.
For fixed effects, we used:

1. **Normal Priors**
   Normal priors, $\beta \sim \mathcal{N}(0,\sigma^2)$, were used with two different standard deviations (σ). A general weakly informative prior with $\sigma = 1$ was the baseline (see Prior Choice Recommendations of Stan on Github and Gelman et al.[11]). A second, less informative, prior with $\sigma = 2.5$ was used to test sensitivity to prior choice. The first prior was used for the base model configurations, the base model configurations with additional interaction terms, and the further explorative models. The latter is only applied to the base model configuration.

2. **Regularized Horseshoe Prior (RHS)** For the base model, additionally, we used a RHS prior[12], which is particularly well-suited to shrink non-influential variables to zero and retain influential variables in the model, effectively performing a type of automatic variable selection. The regularized horseshoe prior introduces a regularization term to locally shrink uninfluential parameters, thereby preserving influential coefficients. The scale of the global shrinkage prior, τ_0, is set based on an expected number of "effective" non-zero parameters (P_0), and σ, the assumed error variance[13]. Due to the complex nature of our model and a lack of general recommendations, we used multiple P_0 and σ. For the amount of effective parameters P_0, we used three different percentages to calculate P_0 from P via multiplication: 25% – 50% – 75%. For the assumed error variance σ, we used:

   - $\sigma = 1$: Used in regular linear regression context[13]
   - $\sigma = 2$: Proposed for logistic regressions[13]
   - $\sigma = \pi/\sqrt{6}$ as a rough approximation for the Gumbel distribution of the error term in multinomial regressions[14]

3. **Hierarchical Regularized Horseshoe Prior for Interactions** For the model configurations that included additional interaction terms, we applied a hierarchical regularized horseshoe prior, which introduces two distinct groups of coefficients – main effects and interactions – each with its own regularized horseshoe prior, but with different settings for the global shrinkage parameter (τ). This structure allows for different degrees of shrinkage for the two groups. For our application, we only varied the global scale parameter for the interaction effects from 25% to 50% and the assumed error variance σ as described above, excluding the proposal for logistic regressions. The global scale parameter for the main effects was fixed at 50%. This was done, as these model configurations were used to detect important interaction dynamics in our data.

4. **Random Effects Priors** For the random effect models, which in our application represent a respondent-level random slope on one coefficient, the coefficients for the fixed effects (β) were assigned a weakly informative normal prior, $\beta \sim \mathcal{N}(0,1)$. For the standard deviation of the random effect (σ), an exponential prior with a rate of 1 was used, $\sigma \sim \text{Exponential}(1)$. This prior ensures that σ is positive and assigns higher probability to smaller values. The individual random effects were modeled using a non-centered parameterization. The raw (uncentered) effects (γ_{raw}) were given a standard normal prior, $\gamma_{\text{raw}} \sim \mathcal{N}(0,1)$. The centered effects were then derived as $\gamma = \sigma \cdot \gamma_{\text{raw}}$.

6.2 Sampling Configurations

As Stan implements the Hamiltonian Monte Carlo (HMC) method with the No-U-Turn Sampler (NUTS), the Hamiltonian dynamics are led by two key parameters: the step size and the maximum tree depth[10]. The model-specific adapted step size varied between 0.8 (default) and 0.999, while the maximum tree depth for the NUTS algorithm was set between 10 (default) and 15. This fine-tuning was performed to address the complexity of each model and ensure robust and stable estimation.

6.3 Reporting Style

For the reporting of the results, we chose the 90% credible interval as default. This choice was primarily driven by considerations of computational stability, given the complexity of our multinomial choice model and the varying choice sets involved. As noted by McElreath[19], the extreme tails of a posterior distribution are often the most difficult to estimate accurately, and require significantly more MCMC samples to reach high precision. By focusing on the 90% interval, we provide a more stable and reliable summary of the posterior "mass" that is less sensitive to sampling noise in the extreme tails. Given the inherent sensitivity of multinomial models to our complex data structure, we believe the 90% interval offers a more robust representation of our results than the more conventional, but often noisier, 95% interval.

7 MICE Imputation

To test whether missing values in our data set affect the results, we performed a multiple imputation by chained equations (MICE) procedure. Due to the complex structural nature of our dataset, we opted for a parsimonious imputation procedure, in which we only imputed the missing values in the demographic variables used in the model. This had multiple advantages:

1. With this approach, we could focus on the individual-level data. Most of our variables that we are using in the model are inherent dyadic – as they include a receiver and a sender (e.g., A reports that B supports her) – and are time-dependent. This is not the case for the demographic variables, as they are inherent ego-specific and relatively time-stable.

2. Almost all of our variables are interdependent on each other. Meaning that if A would nominate B in a sequence as emotionally supportive. This change in itself changes the likelihood that B would report A as well. Moreover, these nominations are embedded in a social network, which in itself opens significant challenges for handling missing data; for a detailed argumentation, see Krause et al.[15]. This is not the case for the demographic variables again.

3. Of the three demographic variables, one of them are in the lead in terms of missing data percentage over all predictors; Mother tongue with 23% missings for ego-alter and 27% for alter-alter. Therefore, indicating a relatively low number of independent, individual-specific values generates a substantial amount of additional data that can be analyzed within the choice model framework.

Due to all the mentioned reasons, we decided to proceed with the demographic-specific imputation. So we extracted the demographics of each participant per cohort. Demographic data are imputed using all other demographic data as predictors and a random forest method (method = "rf"), which is recommended for categorical values, especially when dealing with many rare events[16], as is the case for mother tongue and nationality. We also included for the imputation model the information from the home canton if available, as this is informative for the native languages of the participants, especially in Switzerland, where the regions have different official languages. For both relation types, only one imputation setup was done, as both share the same participants.

We then imputed 100 different demographic datasets, which is recommended for Bayesian analysis[17], checked the algorithmic convergence of MICE based on trace plots and the imputation convergence based on distribution plots, preprocessed the data exactly the same way as the original data, and calculated the same model with the exact same configurations as the base model with all the different datasets separately. For each of the models, we separately checked the convergence of the HMC algorithm using diagnostic statistics (e.g. rank-normalized split-R statistic, effective sample size)[18] and combined the results by concatenating the posterior draws for each predictor across the 100 datasets. The reported results are based on 4,000 post-warmup draws from four chains, following 2,000 warmup iterations per dataset.

A technical issue arose from the dummy-coding strategy: in a subset of cases, the imputation algorithm imputed a value of zero for all possible language dummies simultaneously, which is logically impossible (every participant must have at least one mother tongue). To avoid the loss of these cases, we performed a necessary post-hoc correction by assigning a language through a random draw from the observed, weighted distribution. This measure, while necessary for data integrity, constitutes an ad-hoc violation of the MAR assumption for the affected subjects, as the imputation for these specific individuals was no longer conditioned on their other demographic information.

Overall, the MICE imputation showed acceptable convergence and generated reasonable distributions of the variables, comparable to the observed distributions (see the following plots and tables). The only weak systematic bias that could not be addressed in the imputation process was a slight underrepresentation of the most frequently occurring group for the nationality and mother tongue variables, and a slight overrepresentation of the second-largest groups. Despite this weak bias, we accepted the imputed datasets as plausible enough to use them further for the analysis due to their proximity to the mean values.

Regarding our post-hoc correction by assigning a language, we observed that in for cohort 1 a maximum of 27% (*Median* = 15.6%) of the missing data cases was post-hoc assigned, in cohort 2 a maximum of 21% (*Median* = 8.6%), and in cohort 3 a maximum of 26% (*Median* = 11.6%). The necessity of the post-hoc correction introduces a limitation, as it means the imputation for these specific cases relies on the marginal distribution rather than the preferred conditional distribution of MICE, thus violating the strict MAR assumption. However, this decision was made as a necessary trade-off to preserve the analytical sample size. Given that the alternative, excluding all individuals where MICE failed to impute a single language, would have resulted in a substantial reduction and introduced an even greater risk of selection bias (i.e., listwise elimination bias), the post hoc correction was deemed the preferable approach. Despite the methodological caveats concerning the mother tongue variable, the final decision to use the imputed dataset rests on its purpose: a robustness check. The results demonstrate that the estimated coefficients (magnitude, direction, and credible intervals) for all predictors remained highly consistent and stable between the imputed and original datasets; if changes occurred, they did not affect the mother tongue variable. This provides confidence that the post-hoc assignment of a single variable did not materially affect the core conclusions of our study. Nevertheless, we remain cautious in interpreting the specific parameter estimates for mother tongue for the models using the MICE dataset, as the described methodological challenge remains.

Table 8. Percentage of Missing Values per Variable in %

	Friendship in Sequence	in Choices	Positive Interaction in Sequence	in Choices
EGO - ALTER				
Gender	0.01	0.09	0.15	0.16
Nationality	0.25	0.23	0.27	0.30
Mother Tongue	23.67	23.09	23.57	23.01
Known before	11.66	15.42	11.49	10.93
Popularity	4.35	4.84	4.91	5.44
Support	4.20	4.76	4.73	5.31
Travel	3.87	4.44	4.23	4.88
Studying	3.85	4.42	4.22	4.87
Free time	3.87	4.44	4.23	4.88
Group nominations	3.88	4.46	4.14	4.82
Frequency of Interaction	23.86	31.99	39.54	49.42
Mutual nomination	31.32	32.83	30.81	31.97
ALTER - ALTER				
Gender	0.18	0.16	0.26	0.27
Nationality	0.47	0.40	0.49	0.52
Mother Tongue	27.82	27.95	28.03	28.13
Known before	20.82	85.62	21.07	21.20
Popularity	4.52	4.51	5.07	5.25
Support	17.07	33.34	16.80	16.50
Travel	4.13	4.15	4.41	4.65
Studying	5.24	5.77	5.12	5.83
Free time	5.70	6.24	5.41	6.13
Shared Group membership	6.18	6.78	5.86	6.80
Name Overlap	0.00	0.00	0.00	0.00
Frequency of Interaction	51.25	69.59	61.54	75.29

Note: Missing values in sequences can produce multiple missing values in the choice format. The missing values for the choices are reported before the additional implementation of using a dummy value for cases of complete missing values for a choice.

Figure 6. Proportion of males per cohort compared to imputed datasets

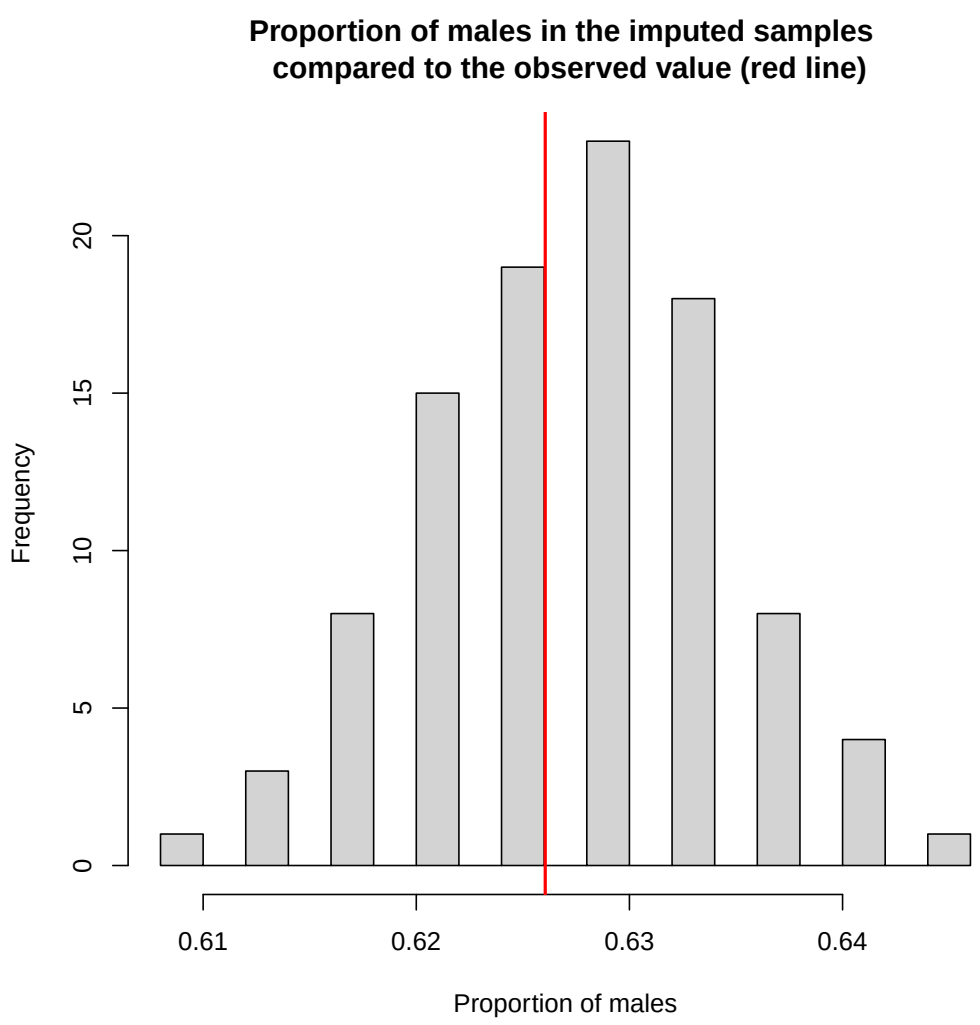

Cohort 1

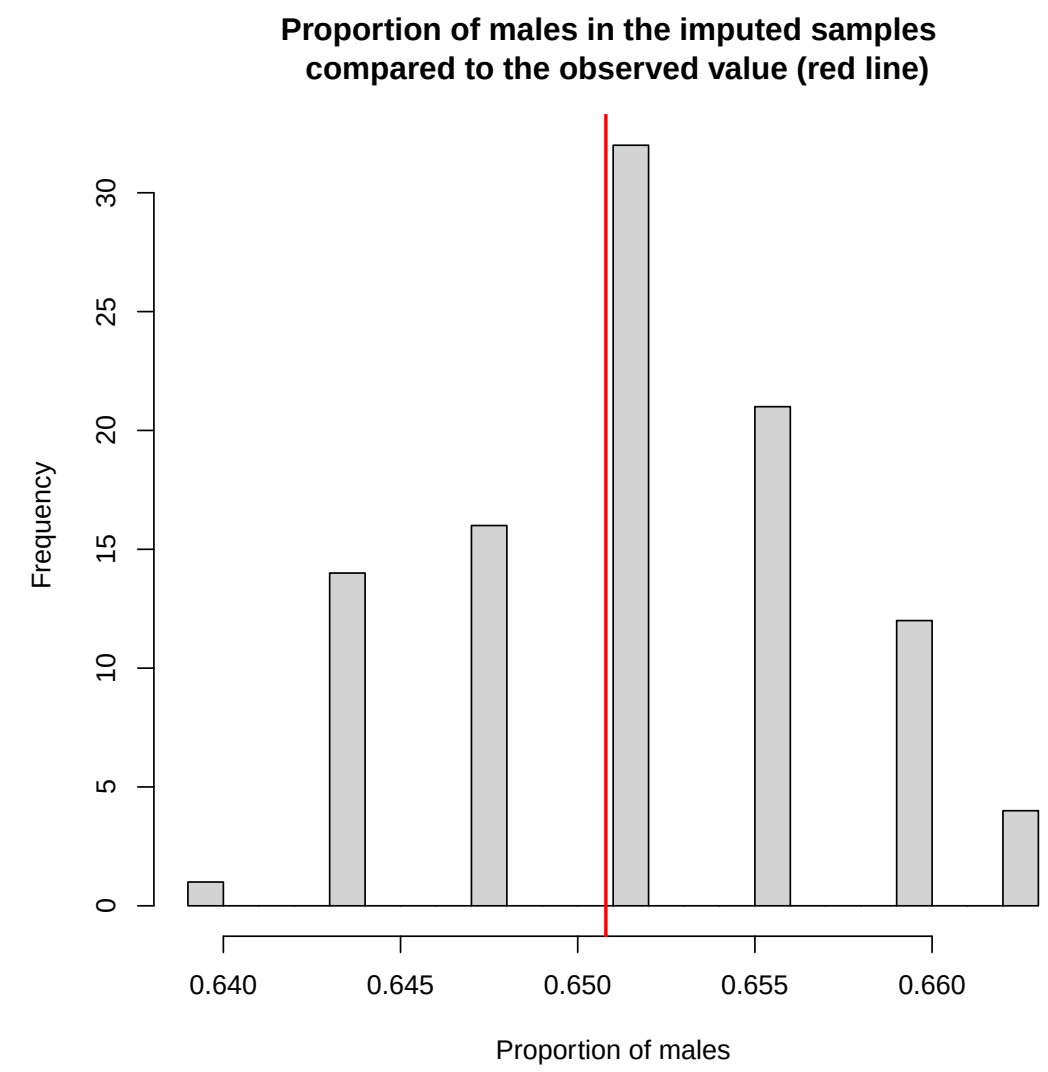

Cohort 2

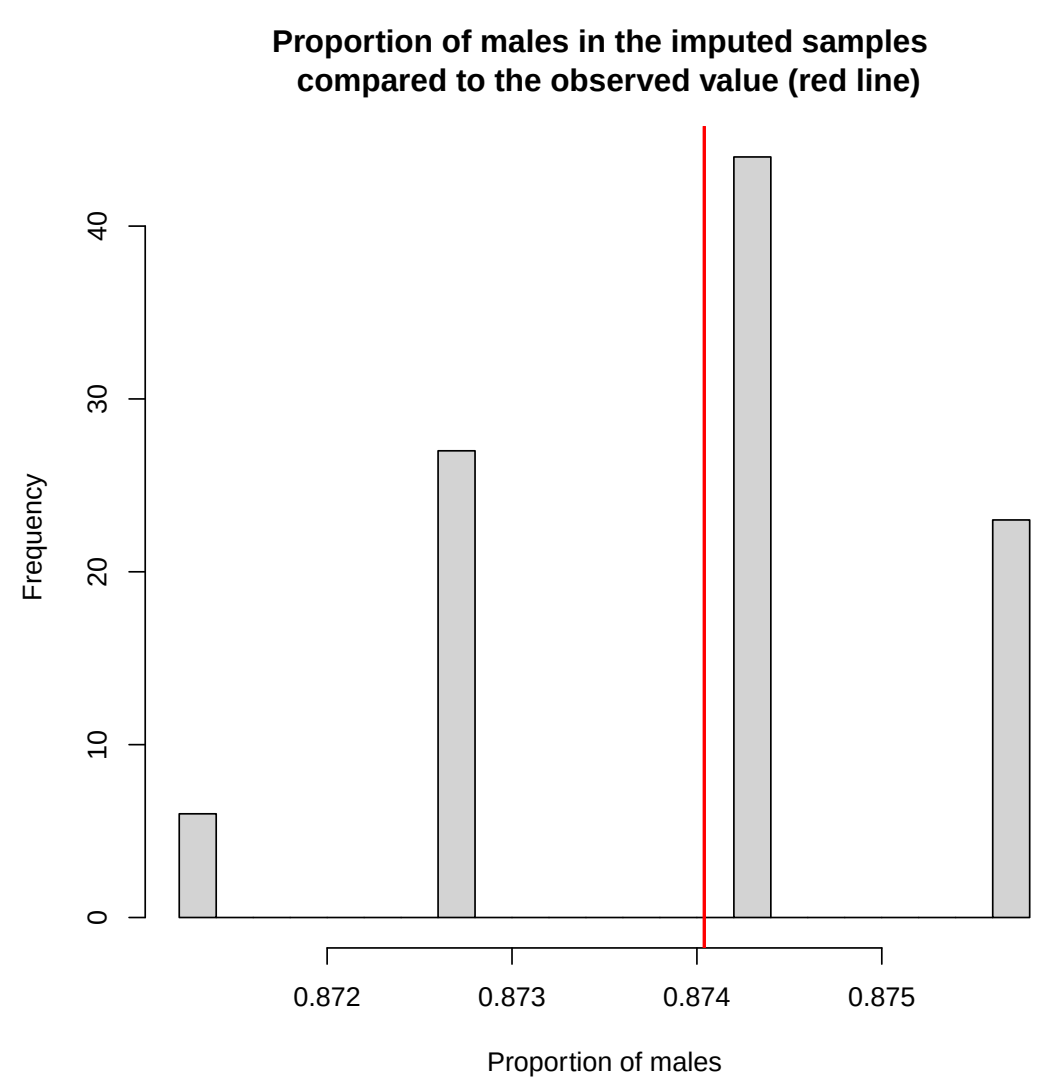

Cohort 3

Figure 7. Distribution of nationalities compared to 100 imputed datasets

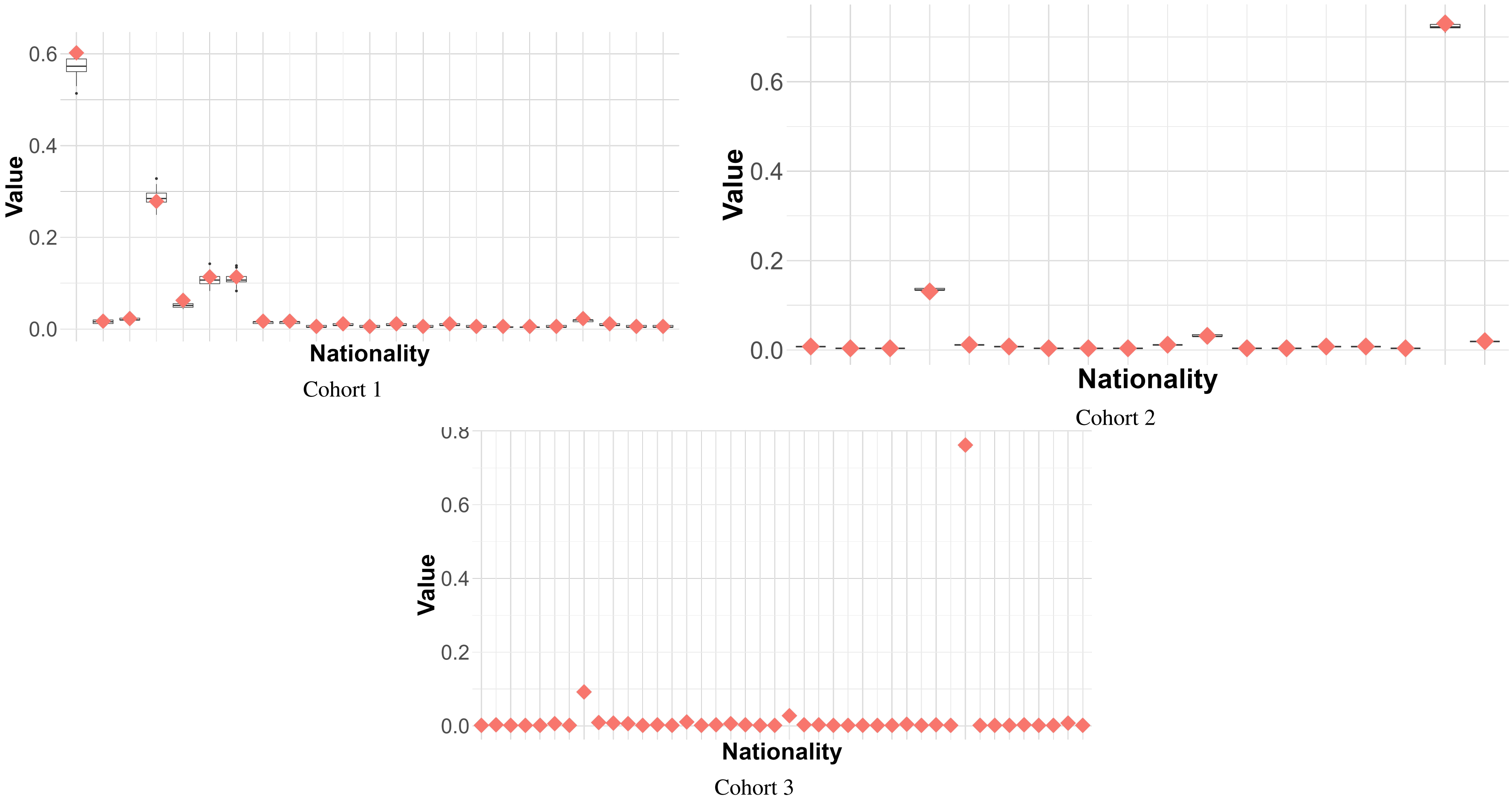

Figure 8. Distribution of mother tongues compared to 100 imputed datasets

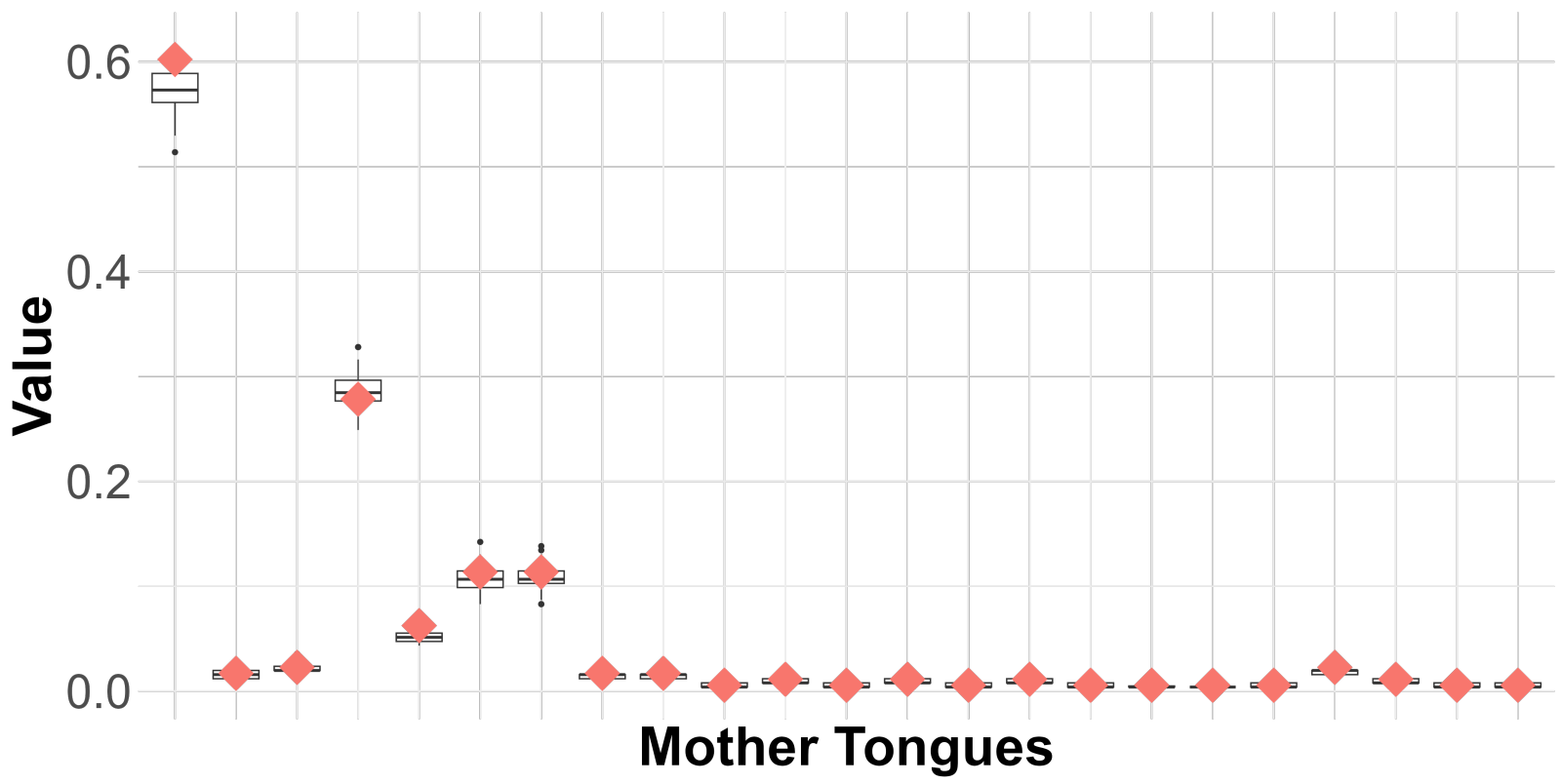

Cohort 1

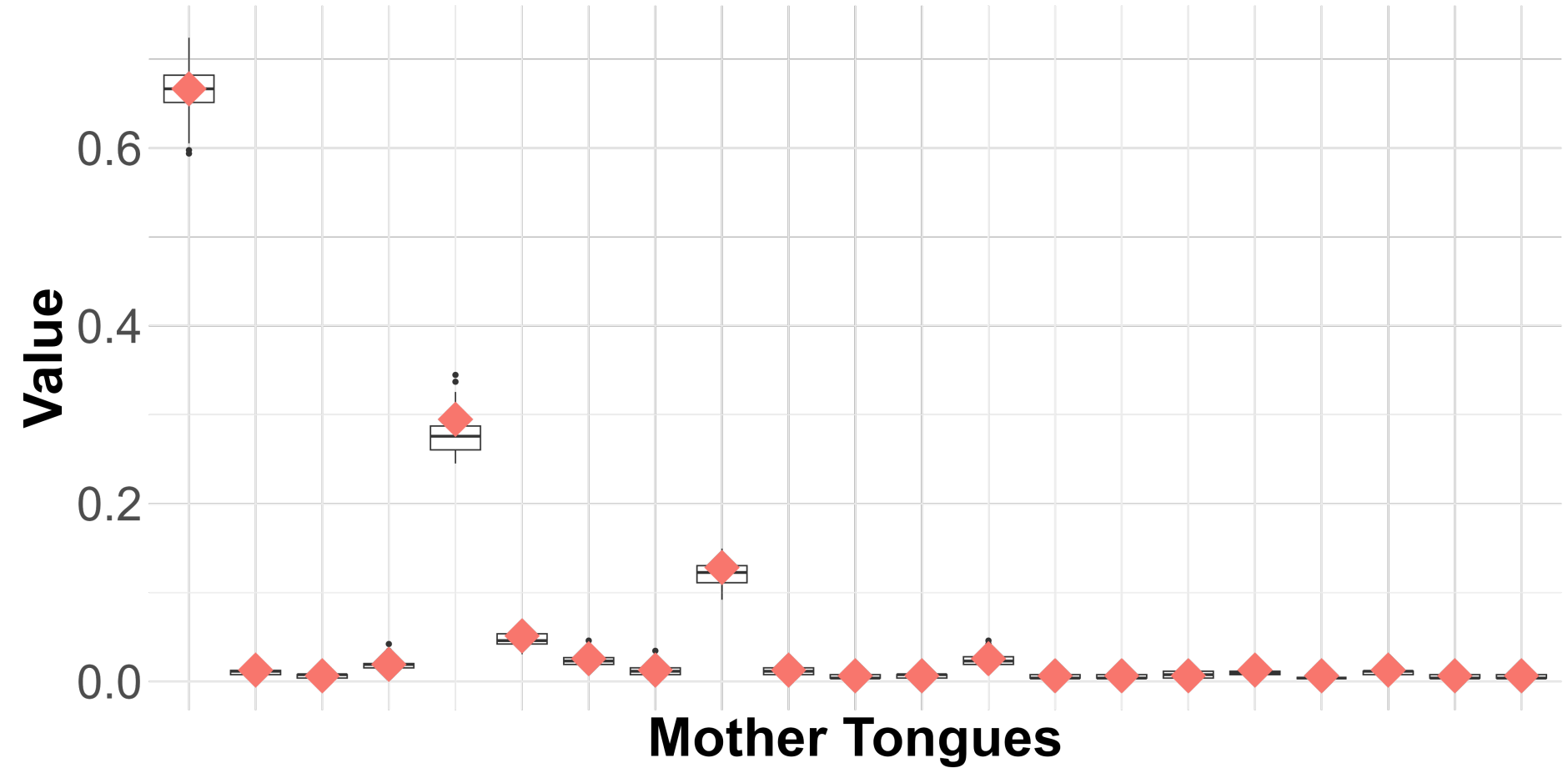

Cohort 2

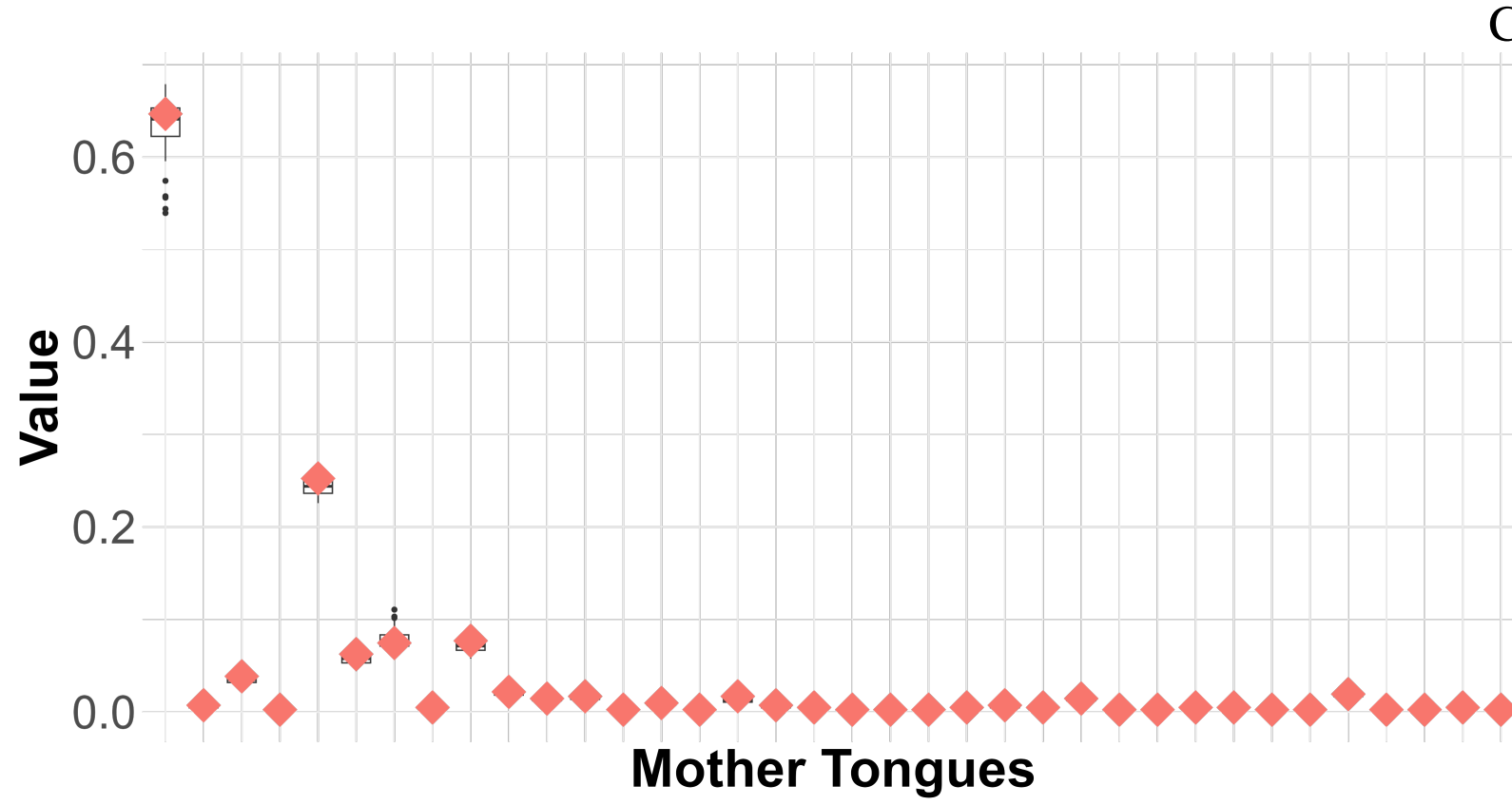

Cohort 3

Table 9. Distribution of Imputed Nationalities Across 100 Datasets compared to the observed values - Cohort 1

Nationality	Mean	SD	Min	Max	$Observed_{Imputation}$
Country 1	0.0047	0.0016	0.0040	0.0079	0.0044
Country 2	0.1596	0.0105	0.1383	0.1976	0.1416
Country 3	0.0142	0.0030	0.0119	0.0277	0.0133
Country 4	0.0089	0.0021	0.0079	0.0158	0.0088
Country 5	0.0049	0.0019	0.0040	0.0119	0.0044
Country 6	0.0048	0.0019	0.0040	0.0119	0.0044
Country 7	0.0045	0.0014	0.0040	0.0079	0.0044
Country 8	0.0045	0.0015	0.0040	0.0119	0.0044
Country 9	0.0051	0.0025	0.0040	0.0158	0.0044
Country 10	0.0148	0.0035	0.0119	0.0277	0.0133
Country 11	0.0048	0.0018	0.0040	0.0119	0.0044
Country 12	0.0631	0.0069	0.0514	0.0830	0.0575
Country 13	0.0044	0.0014	0.0040	0.0119	0.0044
Country 14	0.0047	0.0015	0.0040	0.0079	0.0044
Country 15	0.0046	0.0016	0.0040	0.0119	0.0044
Country 16	0.0136	0.0025	0.0119	0.0198	0.0133
Country 17	0.0101	0.0029	0.0079	0.0198	0.0088
Country 18	0.0049	0.0017	0.0040	0.0079	0.0044
Country 19	0.0047	0.0016	0.0040	0.0079	0.0044
Country 20	0.0045	0.0016	0.0040	0.0119	0.0044
Country 21	0.0045	0.0017	0.0040	0.0158	0.0044
Country 22	0.6285	0.0117	0.6047	0.6601	0.6593
Country 23	0.0045	0.0015	0.0040	0.0119	0.0044
Country 24	0.0040	0.0004	0.0040	0.0079	0.0044
Country 25	0.0047	0.0018	0.0040	0.0119	0.0044
Country 26	0.0042	0.0009	0.0040	0.0079	0.0044
Country 27	0.0045	0.0014	0.0040	0.0119	0.0044

Table 10. Distribution of Imputed Mother Tongue Across 100 Datasets compared to the observed values - Cohort 1

Mother Tongue	Mean	SD	Min	Max	$\text{Observed}_{Imputation}$
Language 1	0.5747	0.0198	0.5138	0.6166	0.6023
Language 2	0.0176	0.0052	0.0119	0.0316	0.0170
Language 3	0.0219	0.0047	0.0158	0.0356	0.0227
Language 4	0.2857	0.0157	0.2490	0.3281	0.2784
Language 5	0.0523	0.0053	0.0435	0.0632	0.0625
Language 6	0.1067	0.0110	0.0830	0.1423	0.1136
Language 7	0.1075	0.0104	0.0830	0.1383	0.1136
Language 8	0.0151	0.0037	0.0119	0.0277	0.0170
Language 9	0.0146	0.0032	0.0119	0.0237	0.0170
Language 10	0.0059	0.0031	0.0040	0.0158	0.0057
Language 11	0.0096	0.0023	0.0079	0.0158	0.0114
Language 12	0.0058	0.0027	0.0040	0.0158	0.0057
Language 13	0.0094	0.0023	0.0079	0.0158	0.0114
Language 14	0.0051	0.0019	0.0040	0.0119	0.0057
Language 15	0.0106	0.0038	0.0079	0.0237	0.0114
Language 16	0.0054	0.0026	0.0040	0.0158	0.0057
Language 17	0.0052	0.0025	0.0040	0.0158	0.0057
Language 18	0.0050	0.0023	0.0040	0.0158	0.0057
Language 19	0.0053	0.0023	0.0040	0.0158	0.0057
Language 20	0.0187	0.0032	0.0158	0.0316	0.0227
Language 21	0.0105	0.0037	0.0079	0.0237	0.0114
Language 22	0.0055	0.0026	0.0040	0.0158	0.0057
Language 23	0.0056	0.0027	0.0040	0.0158	0.0057

Table 11. Distribution of Imputed Nationalities Across 100 Datasets compared to the observed values - Cohort 2

Nationality	Mean	SD	Min	Max	$\text{Observed}_{Imputation}$
Country 1	0.0081	0.0012	0.0077	0.0115	0.0079
Country 2	0.0041	0.0010	0.0038	0.0077	0.0040
Country 3	0.0039	0.0004	0.0038	0.0077	0.0040
Country 4	0.1359	0.0047	0.1264	0.1456	0.1310
Country 5	0.0121	0.0015	0.0115	0.0192	0.0119
Country 6	0.0082	0.0016	0.0077	0.0153	0.0079
Country 7	0.0040	0.0008	0.0038	0.0077	0.0040
Country 8	0.0042	0.0011	0.0038	0.0077	0.0040
Country 9	0.0041	0.0009	0.0038	0.0077	0.0040
Country 10	0.0122	0.0017	0.0115	0.0192	0.0119
Country 11	0.0322	0.0023	0.0307	0.0383	0.0317
Country 12	0.0041	0.0011	0.0038	0.0115	0.0040
Country 13	0.0039	0.0005	0.0038	0.0077	0.0040
Country 14	0.0080	0.0011	0.0077	0.0115	0.0079
Country 15	0.0082	0.0013	0.0077	0.0115	0.0079
Country 16	0.0041	0.0011	0.0038	0.0115	0.0040
Country 17	0.7226	0.0056	0.7088	0.7356	0.7302
Country 18	0.0202	0.0019	0.0192	0.0268	0.0198

Table 12. Distribution of Imputed Mother Tongue Across 100 Datasets compared to the observed values - Cohort 2

Mother Tongue	Mean	SD	Min	Max	$\text{Observed}_{Imputation}$
Language 1	0.6639	0.0254	0.5939	0.7241	0.6667
Language 2	0.0111	0.0035	0.0077	0.0230	0.0128
Language 3	0.0068	0.0032	0.0038	0.0192	0.0064
Language 4	0.0183	0.0055	0.0115	0.0421	0.0192
Language 5	0.2781	0.0208	0.2452	0.3448	0.2949
Language 6	0.0466	0.0078	0.0307	0.0651	0.0513
Language 7	0.0238	0.0057	0.0153	0.0460	0.0256
Language 8	0.0124	0.0049	0.0077	0.0345	0.0128
Language 9	0.1210	0.0129	0.0920	0.1494	0.1282
Language 10	0.0121	0.0039	0.0077	0.0230	0.0128
Language 11	0.0051	0.0024	0.0038	0.0153	0.0064
Language 12	0.0068	0.0034	0.0038	0.0192	0.0064
Language 13	0.0247	0.0063	0.0153	0.0460	0.0256
Language 14	0.0049	0.0019	0.0038	0.0115	0.0064
Language 15	0.0061	0.0028	0.0038	0.0153	0.0064
Language 16	0.0076	0.0037	0.0038	0.0192	0.0064
Language 17	0.0103	0.0033	0.0077	0.0230	0.0128
Language 18	0.0050	0.0023	0.0038	0.0153	0.0064
Language 19	0.0108	0.0038	0.0077	0.0230	0.0128
Language 20	0.0057	0.0027	0.0038	0.0153	0.0064
Language 21	0.0057	0.0029	0.0038	0.0153	0.0064

Table 13. Distribution of Imputed Nationalities Across 100 Datasets compared to the observed values - Cohort 3

Nationality	Mean	SD	Min	Max	$\text{Observed}_{Imputation}$
Nationality 1	0.0015	0.0002	0.0015	0.0030	0.0015
Nationality 2	0.0030	0.0002	0.0030	0.0045	0.0031
Nationality 3	0.0015	0.0000	0.0015	0.0015	0.0015
Nationality 4	0.0015	0.0002	0.0015	0.0030	0.0015
Nationality 5	0.0016	0.0003	0.0015	0.0030	0.0015
Nationality 6	0.0062	0.0005	0.0061	0.0091	0.0061
Nationality 7	0.0016	0.0003	0.0015	0.0030	0.0015
Nationality 8	0.0940	0.0019	0.0909	0.0985	0.0922
Nationality 9	0.0093	0.0005	0.0091	0.0106	0.0092
Nationality 10	0.0078	0.0005	0.0076	0.0091	0.0077
Nationality 11	0.0062	0.0004	0.0061	0.0076	0.0061
Nationality 12	0.0015	0.0002	0.0015	0.0030	0.0015
Nationality 13	0.0031	0.0004	0.0030	0.0045	0.0031
Nationality 14	0.0015	0.0002	0.0015	0.0030	0.0015
Nationality 15	0.0109	0.0006	0.0106	0.0136	0.0108
Nationality 16	0.0016	0.0004	0.0015	0.0030	0.0015
Nationality 17	0.0032	0.0005	0.0030	0.0045	0.0031
Nationality 18	0.0061	0.0003	0.0061	0.0076	0.0061
Nationality 19	0.0031	0.0003	0.0030	0.0045	0.0031
Nationality 20	0.0016	0.0003	0.0015	0.0030	0.0015
Nationality 21	0.0015	0.0002	0.0015	0.0030	0.0015
Nationality 22	0.0281	0.0010	0.0273	0.0303	0.0276
Nationality 23	0.0031	0.0002	0.0030	0.0045	0.0031
Nationality 24	0.0031	0.0005	0.0030	0.0061	0.0031
Nationality 25	0.0016	0.0003	0.0015	0.0030	0.0015
Nationality 26	0.0015	0.0002	0.0015	0.0030	0.0015
Nationality 27	0.0016	0.0003	0.0015	0.0030	0.0015
Nationality 28	0.0015	0.0002	0.0015	0.0030	0.0015
Nationality 29	0.0015	0.0002	0.0015	0.0030	0.0015
Nationality 30	0.0046	0.0004	0.0045	0.0061	0.0046
Nationality 31	0.0015	0.0002	0.0015	0.0030	0.0015
Nationality 32	0.0032	0.0004	0.0030	0.0045	0.0031
Nationality 33	0.0015	0.0000	0.0015	0.0015	0.0015
Nationality 34	0.7584	0.0024	0.7530	0.7636	0.7619
Nationality 35	0.0015	0.0000	0.0015	0.0015	0.0015
Nationality 36	0.0016	0.0003	0.0015	0.0030	0.0015
Nationality 37	0.0016	0.0003	0.0015	0.0030	0.0015
Nationality 38	0.0031	0.0004	0.0030	0.0061	0.0031
Nationality 39	0.0015	0.0000	0.0015	0.0015	0.0015
Nationality 40	0.0015	0.0002	0.0015	0.0030	0.0015
Nationality 41	0.0078	0.0006	0.0076	0.0091	0.0077
Nationality 42	0.0015	0.0002	0.0015	0.0030	0.0015

Table 14. Distribution of Imputed Mother Tongue Across 100 Datasets compared to the observed values - Cohort 3

Mother Tongue	Mean	SD	Min	Max	$\text{Observed}_{Imputation}$
Language 1	0.6352	0.0263	0.5394	0.6788	0.6466
Language 2	0.0066	0.0018	0.0045	0.0121	0.0072
Language 3	0.0353	0.0042	0.0258	0.0470	0.0385
Language 4	0.0022	0.0010	0.0015	0.0061	0.0024
Language 5	0.2439	0.0094	0.2258	0.2667	0.2524
Language 6	0.0577	0.0054	0.0439	0.0712	0.0625
Language 7	0.0777	0.0095	0.0606	0.1106	0.0745
Language 8	0.0035	0.0009	0.0030	0.0061	0.0048
Language 9	0.0707	0.0060	0.0576	0.0864	0.0769
Language 10	0.0200	0.0033	0.0136	0.0288	0.0216
Language 11	0.0138	0.0028	0.0091	0.0227	0.0144
Language 12	0.0155	0.0030	0.0106	0.0242	0.0168
Language 13	0.0022	0.0010	0.0015	0.0061	0.0024
Language 14	0.0082	0.0019	0.0061	0.0152	0.0096
Language 15	0.0020	0.0009	0.0015	0.0061	0.0024
Language 16	0.0123	0.0016	0.0106	0.0182	0.0168
Language 17	0.0068	0.0021	0.0045	0.0136	0.0072
Language 18	0.0038	0.0012	0.0030	0.0091	0.0048
Language 19	0.0021	0.0010	0.0015	0.0076	0.0024
Language 20	0.0021	0.0011	0.0015	0.0061	0.0024
Language 21	0.0020	0.0008	0.0015	0.0045	0.0024
Language 22	0.0042	0.0014	0.0030	0.0076	0.0048
Language 23	0.0065	0.0019	0.0045	0.0136	0.0072
Language 24	0.0042	0.0012	0.0030	0.0076	0.0048
Language 25	0.0134	0.0025	0.0091	0.0212	0.0144
Language 26	0.0020	0.0008	0.0015	0.0045	0.0024
Language 27	0.0019	0.0008	0.0015	0.0045	0.0024
Language 28	0.0040	0.0014	0.0030	0.0106	0.0048
Language 29	0.0044	0.0015	0.0030	0.0076	0.0048
Language 30	0.0019	0.0009	0.0015	0.0061	0.0024
Language 31	0.0025	0.0011	0.0015	0.0061	0.0024
Language 32	0.0177	0.0026	0.0121	0.0258	0.0192
Language 33	0.0022	0.0010	0.0015	0.0061	0.0024
Language 34	0.0022	0.0010	0.0015	0.0061	0.0024
Language 35	0.0044	0.0014	0.0030	0.0091	0.0048
Language 36	0.0021	0.0009	0.0015	0.0061	0.0024

8 Glossary - Model Identification

- OC – Other choices; representing the subsequent choices (SC).
- FC – First choice; representing the initial choices (FC).
- FE – Fixed effects.
- RE – Random effect. Indicates that the model includes a random slope on the following specified predictor.
- RHS – Regularized Horse Shoe prior.

  rhsXXX: Numbers after rhs indicate the assumed effective parameters (025 = 25%, 05 = 50%, 075 = 75%) for the RHS.

  rhsXXX_type: Type is indicating which sigma prior is used for the RHS (default/no indicator = 1; gumbel = $\pi/\sqrt{6}$; logreg = 2).

- EA – Ego-Alter.
- AA – Alter-Alter.
- weaker_prior – Model with weaker priors ($\beta \sim \mathcal{N}(0, 2.5)$).
- Choice – Indicator that the model includes interactions based on the position of the choices in the sequence.
- ChoiceL1 – Indicator that the model includes interactions based on the position of the choices in the sequence; simplified to the first two nominations versus all afterwards.
- Loglength – Indicator that the model includes interactions based on the length of the overall sequence.
- Wave – Indicator that the model includes interactions based on the time point of the sequence.
- WaveL1 (for Initial Choice: L1) – Indicator that the model includes interactions based on the time point of the sequence; simplified to the first time point versus all afterwards.
- ZxY (e.g., choicexwaves) – Indicator that the model includes interactions based on Z (here: the time point of the sequence) and Y (here: the position of the choices in the sequence)

9 LOO Results

Here, the raw LOO results are shown for all four different model comparisons.

Table 15. LOO - Results for Initial Choice - Friendship

Models	elpd_loo	SE_{elpd_loo}	p_loo	SE_{p_loo}
fc_fe_plain	-2189.61	37.10	10.33	0.26
fc_fe_plain_weaker_prior25	-2189.72	37.20	10.45	0.27
fc_fe_rhs075	-2189.76	36.89	10.32	0.26
fc_fe_rhs05	-2189.95	36.87	10.49	0.27
fc_fe_rhs025	-2189.90	36.89	10.46	0.27
fc_fe_rhs05_gumbel	-2189.78	36.88	10.34	0.26
fc_fe_rhs05_logreg	-2189.87	36.88	10.43	0.27
fc_fe_wave_interactionall	-2199.67	37.56	39.06	1.12
fc_fe_wave_interactionRHS_rhs05	-2190.51	36.90	13.17	0.34
fc_fe_wave_interactionRHS_rhs025	-2190.14	36.89	11.96	0.31
fc_fe_wave_interactionRHS_rhs025_gumbel	-2190.20	36.90	12.20	0.31
fc_fe_L1_interactionall	-2189.23	37.25	19.35	0.92
fc_fe_L1_interactionRHS_rhs05	-2189.24	36.90	13.69	0.49
fc_fe_L1_interactionRHS_rhs025	-2189.48	36.90	12.56	0.40
fc_fe_L1_interactionRHS_rhs025_gumbel	-2189.61	36.88	12.90	0.42
fc_fe_loglength_interactionall	-2192.88	37.31	20.53	0.69
fc_fe_loglength_interactionRHS_rhs05	-2189.78	36.88	13.32	0.39
fc_fe_loglength_interactionRHS_rhs025	-2189.78	36.88	12.45	0.35
fc_fe_loglength_interactionRHS_rhs025_gumbel	-2189.74	36.89	12.63	0.36
fc_re_ea_gender	-2179.34	37.34	68.85	2.79
fc_re_ea_natio	-2169.46	37.44	86.61	3.85
fc_re_ea_lang	-2171.28	37.60	88.66	3.57
fc_re_ea_popular	-2165.71	37.79	113.49	5.37
fc_re_ea_support	-2156.87	37.07	155.41	7.20
fc_re_ea_freetime	-2185.67	37.25	81.47	3.83
fc_re_ea_study	-2187.74	37.08	62.34	2.49
fc_re_ea_travel	-2186.89	37.16	54.05	2.21
fc_re_ea_known_before	-2183.42	37.26	58.68	3.12
fc_re_ea_groupmember	-2190.44	37.17	24.10	1.32

Note: The best fitting model is defined by the model with the highest elpd_loo (for negative values with the elpd_loo nearest to zero). p_loo is difficult to interpret in the presence of random slopes, indicated with "_re_" in the model label.

Table 16. LOO - Results for Subsequent Choices - Friendship

Models	elpd_loo	SE_{elpd_loo}	p_loo	SE_{p_loo}
oc_fe_plain	-6001.46	48.90	22.20	0.35
oc_fe_plain_weaker_prior25	-6001.75	48.97	22.51	0.37
oc_fe_rhs075	-6000.63	48.54	20.53	0.29
oc_fe_rhs05	-6000.62	48.54	20.51	0.29
oc_fe_rhs025	-6000.50	48.55	20.38	0.29
oc_fe_rhs05_gumbel	-6000.64	48.54	20.53	0.29
oc_fe_rhs05_logreg	-6000.64	48.55	20.54	0.29
oc_fe_wave_interactionall	-6020.35	49.78	82.43	1.47
oc_fe_wave_interactionRHS_rhs05	-5996.50	48.63	30.72	0.42
oc_fe_wave_interactionRHS_rhs025	-5996.72	48.60	29.01	0.39
oc_fe_wave_interactionRHS_rhs025_gumbel	-5996.78	48.60	29.39	0.40
oc_fe_waveL1_interactionall	-6011.07	49.14	40.78	1.28
oc_fe_waveL1_interactionRHS_rhs05	-6001.11	48.54	21.49	0.31
oc_fe_waveL1_interactionRHS_rhs025	-6000.81	48.55	20.98	0.30
oc_fe_waveL1_interactionRHS_rhs025_gumbel	-6000.86	48.55	21.10	0.30
oc_fe_choice_interactionall	-5979.48	49.93	64.03	1.06
oc_fe_choice_interactionRHS_rhs05	-5972.32	48.92	35.45	0.50
oc_fe_choice_interactionRHS_rhs025	-5972.41	48.90	34.15	0.48
oc_fe_choice_interactionRHS_rhs025_gumbel	-5972.48	48.92	34.49	0.48
oc_fe_choiceL1_interactionall	-5971.41	49.69	43.09	0.65
oc_fe_choiceL1_interactionRHS_rhs05	-5969.84	48.95	30.62	0.42
oc_fe_choiceL1_interactionRHS_rhs025	-5970.26	48.94	30.21	0.41
oc_fe_choiceL1_interactionRHS_rhs025_gumbel	-5970.16	48.93	30.28	0.41
oc_fe_wave_choice_interactionall	-6000.43	50.80	124.18	2.02
oc_fe_choicexwave_interactionRHS_rhs05	-5968.51	48.95	45.16	0.60
oc_fe_choicexwave_interactionRHS_rhs025	-5968.46	48.94	43.18	0.57
oc_fe_choicexwave_interactionRHS_rhs025_gumbel	-5968.64	48.94	43.70	0.58
oc_fe_waveL1_choiceL1_interactionall	-5983.24	49.88	61.91	1.43
oc_fe_choiceL1waveL1_interactionRHS_rhs05	-5971.70	48.92	31.14	0.44
oc_fe_choiceL1waveL1_interactionRHS_rhs025	-5971.85	48.91	29.89	0.42
oc_fe_choiceL1waveL1_interactionRHS_rhs025_gumbel	-5971.85	48.93	30.16	0.42
oc_fe_loglength_interact	-5985.02	49.61	41.85	0.77
oc_fe_loglength_interactionRHS_rhs05	-5981.77	48.82	30.39	0.46
oc_fe_loglength_interactionRHS_rhs025	-5982.18	48.81	29.90	0.45
oc_fe_loglength_interactionRHS_rhs025_gumbel	-5982.25	48.80	30.02	0.45
oc_re_ea_gender	-5987.70	49.02	108.29	2.07
oc_re_ea_natio	-5988.52	49.08	103.45	2.29
oc_re_ea_lang	-5999.30	48.94	61.62	1.03
oc_re_ea_pop	-5999.63	48.88	57.48	1.31
oc_re_ea_knownbefore	-5996.67	48.94	62.04	1.56
oc_re_ea_groupmember	-6001.76	48.94	57.79	1.34
oc_re_ea_support	-5975.76	48.98	151.19	2.94
oc_re_ea_freetime	-5995.77	48.79	84.14	1.71
oc_re_ea_study	-5994.44	48.78	93.40	1.86
oc_re_ea_travel	-6001.30	48.90	52.54	1.19
oc_re_aa_gender	-5988.27	49.08	106.53	2.06
oc_re_aa_natio	-5994.77	49.00	92.03	1.91
oc_re_aa_lang	-5996.84	49.02	76.73	1.60
oc_re_aa_knownbefore	-6002.15	48.91	26.15	1.04
oc_re_aa_study	-6000.27	48.84	61.53	1.52
oc_re_aa_travel	-5999.66	48.87	43.69	1.63
oc_re_aa_freetime	-5998.04	48.84	71.33	1.84
oc_re_aa_percievedgroup	-6000.59	48.96	60.45	1.46
oc_re_aa_name	-5996.17	48.96	68.23	3.94
oc_re_aa_popular	-6001.95	48.90	28.60	0.66
oc_re_aa_support	-5996.33	48.95	87.42	1.82

Note: The best fitting model is defined by the model with the highest elpd_loo (for negative values with the elpd_loo nearest to zero). p_loo is difficult to interpret in the presence of random slopes, indicated with "_re_" in the model label.

Table 17. LOO - Results for Initial Choice - Positive Interaction

Models	elpd_loo	SE_{elpd_loo}	p_loo	SE_{p_loo}
fc_fe_plain	-2078.37	40.87	10.09	0.27
fc_fe_plain_weaker_prior25	-2078.43	40.98	10.17	0.28
fc_fe_rhs075	-2078.45	40.66	10.05	0.27
fc_fe_rhs05	-2078.44	40.65	10.03	0.27
fc_fe_rhs025	-2078.43	40.66	10.03	0.27
fc_fe_rhs05_gumbel	-2078.34	40.66	9.94	0.27
fc_fe_rhs05_logreg	-2078.42	40.67	10.02	0.27
fc_fe_wave_interactionall	-2083.73	41.61	38.02	1.13
fc_fe_wave_interactionRHS_rhs05	-2077.97	40.71	16.70	0.50
fc_fe_wave_interactionRHS_rhs025	-2078.33	40.69	14.53	0.42
fc_fe_wave_interactionRHS_rhs025_gumbel	-2078.00	40.74	17.21	0.52
fc_fe_L1_interactionall	-2080.51	41.12	19.54	0.82
fc_fe_L1_interactionRHS_rhs05	-2078.61	40.68	12.24	0.39
fc_fe_L1_interactionRHS_rhs025	-2078.51	40.66	11.27	0.33
fc_fe_L1_interactionRHS_rhs025_gumbel	-2078.70	40.67	12.56	0.40
fc_fe_loglength_interactionall	-2067.66	40.99	20.49	0.73
fc_fe_loglength_interactionRHS_rhs05	-2065.19	40.54	15.18	0.48
fc_fe_loglength_interactionRHS_rhs025	-2065.42	40.52	14.74	0.46
fc_fe_loglength_interactionRHS_rhs025_gumbel	-2065.19	40.54	15.33	0.49
fc_re_ea_gender	-2062.69	41.03	85.75	3.87
fc_re_ea_natio	-2071.89	40.98	65.39	3.20
fc_re_ea_lang	-2073.03	41.04	61.37	2.76
fc_re_ea_popular	-2077.38	40.89	28.75	1.27
fc_re_ea_support	-2041.17	40.60	153.61	7.13
fc_re_ea_freetime	-2075.38	40.81	76.54	3.73
fc_re_ea_study	-2056.24	40.51	121.68	5.55
fc_re_ea_travel	-2071.35	40.78	75.85	3.52
fc_re_ea_known_before	-2061.87	40.73	66.75	4.05
fc_re_ea_groupmember	-2070.67	40.71	69.11	5.23

Note: The best fitting model is defined by the model with the highest elpd_loo (for negative values with the elpd_loo nearest to zero). p_loo is difficult to interpret in the presence of random slopes, indicated with "_re_" in the model label.

Table 18. LOO - Results for Subsequent Choices - Positive Interaction

Models	elpd_loo	SE_{elpd_loo}	p_loo	SE_{p_loo}
oc_fe_plain	-12485.77	73.13	22.49	0.27
oc_fe_plain_weaker_prior25	-12485.85	73.19	22.60	0.27
oc_fe_rhs075	-12485.34	72.91	21.22	0.29
oc_fe_rhs05	-12485.27	72.91	21.13	0.29
oc_fe_rhs025	-12485.17	72.92	21.04	0.29
oc_fe_rhs05_gumbel	-12485.45	72.91	21.31	0.29
oc_fe_rhs05_logreg	-12485.22	72.91	21.10	0.29
oc_fe_wave_interactionall	-12492.39	73.88	84.91	1.12
oc_fe_wave_interactionRHS_rhs05	-12484.57	72.92	31.68	0.47
oc_fe_wave_interactionRHS_rhs025	-12484.92	72.92	28.40	0.42
oc_fe_wave_interactionRHS_rhs025_gumbel	-12484.99	72.91	29.12	0.43
oc_fe_waveL1_interactionall	-12474.66	73.49	42.81	0.95
oc_fe_waveL1_interactionRHS_rhs05	-12471.66	73.05	29.06	0.49
oc_fe_waveL1_interactionRHS_rhs025	-12472.05	73.04	28.93	0.49
oc_fe_waveL1_interactionRHS_rhs025_gumbel	-12472.03	73.05	28.96	0.49
oc_fe_choice_interactionall	-12423.35	74.23	65.62	0.79
oc_fe_choice_interactionRHS_rhs05	-12412.41	73.55	35.11	0.40
oc_fe_choice_interactionRHS_rhs025	-12412.48	73.54	34.34	0.39
oc_fe_choice_interactionRHS_rhs025_gumbel	-12412.72	73.52	34.67	0.40
oc_fe_choiceL1_interactionall	-12441.64	73.71	44.09	0.63
oc_fe_choiceL1_interactionRHS_rhs05	-12436.12	73.26	25.80	0.35
oc_fe_choiceL1_interactionRHS_rhs025	-12436.14	73.26	24.88	0.34
oc_fe_choiceL1_interactionRHS_rhs025_gumbel	-12436.09	73.26	24.99	0.33
oc_fe_waveL1_choiceL1_interactionall	-12433.23	74.00	64.66	1.16
oc_fe_choiceL1waveL1_interactionRHS_rhs05	-12423.84	73.35	34.87	0.53
oc_fe_choiceL1waveL1_interactionRHS_rhs025	-12423.77	73.35	34.26	0.52
oc_fe_choiceL1waveL1_interactionRHS_rhs025_gumbel	-12423.93	73.35	34.37	0.52
oc_fe_wave_choice_interactionall	-12430.74	74.93	128.07	1.55
oc_fe_choicexwave_interactionRHS_rhs05	-12410.03	73.53	48.74	0.60
oc_fe_choicexwave_interactionRHS_rhs025	-12410.79	73.52	46.99	0.58
oc_fe_choicexwave_interactionRHS_rhs025_gumbel	-12410.86	73.51	47.44	0.59
oc_fe_loglength_interact	-12425.17	73.73	42.73	0.59
oc_fe_loglength_interactionRHS_rhs05	-12425.83	73.14	35.63	0.47
oc_fe_loglength_interactionRHS_rhs025	-12425.62	73.14	35.17	0.46
oc_fe_loglength_interactionRHS_rhs025_gumbel	-12425.67	73.13	35.31	0.47
oc_re_ea_gender	-12480.21	73.21	93.16	1.14
oc_re_ea_natio	-12486.05	73.15	41.71	0.48
oc_re_ea_lang	-12485.38	73.15	46.75	0.50
oc_re_ea_pop	-12479.50	73.22	79.69	1.70
oc_re_ea_knownbefore	-12478.85	73.26	74.98	1.69
oc_re_ea_groupmember	-12466.51	73.35	144.73	2.64
oc_re_ea_support	-12451.06	73.42	180.13	3.02
oc_re_ea_freetime	-12480.83	73.21	89.48	1.51
oc_re_ea_study	-12484.57	73.15	72.23	1.12
oc_re_ea_travel	-12484.68	73.20	62.43	1.30
oc_re_aa_gender	-12475.50	73.28	111.12	1.42
oc_re_aa_natio	-12485.67	73.16	56.63	0.70
oc_re_aa_lang	-12484.16	73.18	64.90	0.89
oc_re_aa_knownbefore	-12484.43	73.17	41.85	2.69
oc_re_aa_study	-12484.80	73.08	61.48	1.42
oc_re_aa_travel	-12485.49	73.11	37.80	1.09
oc_re_aa_freetime	-12482.62	73.18	77.94	1.96
oc_re_aa_percievedgroup	-12452.03	73.24	180.29	4.26
oc_re_aa_name	-12476.23	73.36	96.58	4.34
oc_re_aa_popular	-12486.53	73.15	31.18	0.74
oc_re_aa_support	-12469.80	73.23	137.86	2.40

Note: The best fitting model is defined by the model with the highest elpd_loo (for negative values with the elpd_loo nearest to zero). p_loo is difficult to interpret in the presence of random slopes, indicated with "_re_" in the model label.

Table 19. LOO - Results for Exploratory Robustness Checks

Models	elpd_loo	SE_{elpd_loo}	p_loo	SE_{p_loo}
EGO-ALTER - Friendship				
fc_fe_rob_freq	-1509.88	30.22	10.97	0.40
fc_fe_rob_recip	-1023.86	26.08	10.98	0.41
fc_fe_rob_socialcontext_agg	-2192.19	36.81	8.33	0.22
fc_fe_rob_canton	-1293.86	29.66	11.20	0.39
fc_fe_redchoice	-3391.72	48.82	10.35	0.22
ALTER-ALTER - Friendship				
oc_fe_rob_freq	-2806.82	28.59	23.69	0.53
oc_fe_rob_recip	-3119.25	33.33	22.63	0.47
oc_fe_rob_name2	-6040.64	48.86	21.96	0.34
oc_fe_rob_socialcontext_agg	-6017.69	48.78	18.12	0.32
oc_fe_rob_canton	-3180.35	35.15	24.22	0.49
oc_fe_redchoice	-9200.67	63.71	22.19	0.26
EGO-ALTER - Positive Interaction				
fc_fe_rob_freq	-917.64	25.60	10.48	0.49
fc_fe_rob_recip	-791.01	24.93	11.02	0.49
fc_fe_rob_socialcontext_agg	-2085.51	40.55	8.09	0.23
fc_fe_rob_canton	-1084.87	29.53	11.05	0.42
fc_fe_redchoice	-4598.24	62.43	10.45	0.19
ALTER-ALTER - Positive Interaction				
oc_fe_rob_freq	-3534.06	30.51	23.70	0.54
oc_fe_rob_recip	-5495.95	44.86	23.36	0.39
oc_fe_rob_name2	-12578.30	73.12	22.32	0.26
oc_fe_rob_socialcontext_agg	-12513.89	72.85	18.13	0.24
oc_fe_rob_canton	-5096.64	45.31	24.21	0.40
oc_fe_redchoice	-26215.17	113.32	22.13	0.16

Note: Due to divergent datasets used in their estimation, these results cannot be compared directly (neither with one another nor within their model family). We report all results for transparency.

10 LOO Comparison Results without Correction

Here, the uncorrected LOO-comparisons are reported for a holistic perspective. Due to the number of models to compare and our step-wise procedure, the interpretation of the uncorrected LOO-comparison is not recommended as it is biased (see Section 11).

Table 20. LOO Comparison - Results for Initial Choice - Friendship

Model	elpd_diff	SE_{elpd_diff}
fc_re_ea_support	0.0	0.0
fc_re_ea_popular	-8.8	11.6
fc_re_ea_natio	-12.6	10.4
fc_re_ea_lang	-14.4	10.4
fc_re_ea_gender	-22.5	9.3
fc_re_ea_known_before	-26.5	9.3
fc_re_ea_freetime	-28.8	9.4
fc_re_ea_travel	-30.0	9.2
fc_re_ea_study	-30.9	8.9
fc_fe_L1_interactionall	-32.4	9.8
fc_fe_L1_interactionRHS_rhs05	-32.4	9.0
fc_fe_L1_interactionRHS_rhs025	-32.6	8.9
fc_fe_plain	-32.7	8.9
fc_fe_L1_interactionRHS_rhs025_gumbel	-32.7	8.9
fc_fe_plain_weaker_prior25	-32.8	8.9
fc_fe_loglength_interactionRHS_rhs025_gumbel	-32.9	8.8
fc_fe_rhs075	-32.9	8.8
fc_fe_loglength_interactionRHS_rhs05	-32.9	8.8
fc_fe_loglength_interactionRHS_rhs025	-32.9	8.8
fc_fe_rhs05_gumbel	-32.9	8.8
fc_fe_rhs05_logreg	-33.0	8.8
fc_fe_rhs025	-33.0	8.8
fc_fe_rhs05	-33.1	8.8
fc_fe_wave_interactionRHS_rhs025	-33.3	8.9
fc_fe_wave_interactionRHS_rhs025_gumbel	-33.3	8.9
fc_re_ea_groupmember	-33.6	8.9
fc_fe_wave_interactionRHS_rhs05	-33.6	8.9
fc_fe_loglength_interactionall	-36.0	9.4
fc_fe_wave_interactionall	-42.8	10.5

Table 21. LOO Comparison - Results for Subsequent Choice - Friendship

Model	elpd_diff	SE_{elpd_diff}
oc_fe_choicexwave_interactionRHS_rhs025	0.0	0.0
oc_fe_choicexwave_interactionRHS_rhs05	0.0	0.3
oc_fe_choicexwave_interactionRHS_rhs025_gumbel	-0.2	0.1
oc_fe_choiceL1_interactionRHS_rhs05	-1.4	4.8
oc_fe_choiceL1_interactionRHS_rhs025_gumbel	-1.7	4.7
oc_fe_choiceL1_interactionRHS_rhs025	-1.8	4.7
oc_fe_choiceL1_interactionall	-2.9	7.4
oc_fe_choiceL1waveL1_interactionRHS_rhs05	-3.2	4.7
oc_fe_choiceL1waveL1_interactionRHS_rhs025_gumbel	-3.4	4.6
oc_fe_choiceL1waveL1_interactionRHS_rhs025	-3.4	4.6
oc_fe_choice_interactionRHS_rhs05	-3.9	3.7
oc_fe_choice_interactionRHS_rhs025	-3.9	3.6
oc_fe_choice_interactionRHS_rhs025_gumbel	-4.0	3.6
oc_re_ea_support	-7.3	10.9
oc_fe_choice_interactionall	-11.0	7.8
oc_fe_loglength_interactionRHS_rhs05	-13.3	9.7
oc_fe_loglength_interactionRHS_rhs025	-13.7	9.6
oc_fe_loglength_interactionRHS_rhs025_gumbel	-13.8	9.6
oc_fe_waveL1_choiceL1_interactionall	-14.8	8.2
oc_fe_loglength_interact	-16.6	11.1
oc_re_ea_gender	-19.2	10.2
oc_re_aa_gender	-19.8	10.2
oc_re_ea_natio	-20.1	10.0
oc_re_ea_study	-26.0	9.3
oc_re_aa_natio	-26.3	9.4
oc_re_ea_freetime	-27.3	9.0
oc_re_aa_name	-27.7	9.4
oc_re_aa_support	-27.9	9.3
oc_fe_wave_interactionRHS_rhs05	-28.0	7.4
oc_re_ea_knownbefore	-28.2	8.9
oc_fe_wave_interactionRHS_rhs025	-28.3	7.5
oc_fe_wave_interactionRHS_rhs025_gumbel	-28.3	7.5
oc_re_aa_lang	-28.4	9.0
oc_re_aa_freetime	-29.6	8.8
oc_re_ea_lang	-30.8	8.7
oc_re_ea_pop	-31.2	8.6
oc_re_aa_travel	-31.2	8.5
oc_re_aa_study	-31.8	8.6
oc_fe_wave_choice_interactionall	-32.0	9.4
oc_fe_rhs025	-32.0	8.2
oc_re_aa_percievedgroup	-32.1	8.7
oc_fe_rhs05	-32.2	8.2
oc_fe_rhs075	-32.2	8.2
oc_fe_rhs05_gumbel	-32.2	8.2
oc_fe_rhs05_logreg	-32.2	8.2
oc_fe_waveL1_interactionRHS_rhs025	-32.3	8.2
oc_fe_waveL1_interactionRHS_rhs025_gumbel	-32.4	8.2
oc_fe_waveL1_interactionRHS_rhs05	-32.6	8.2
oc_re_ea_travel	-32.8	8.5
oc_fe_plain	-33.0	8.3
oc_fe_plain_weaker_prior25	-33.3	8.3
oc_re_ea_groupmember	-33.3	8.6
oc_re_aa_popular	-33.5	8.3
oc_re_aa_knownbefore	-33.7	8.3
oc_fe_waveL1_interactionall	-42.6	9.0
oc_fe_wave_interactionall	-51.9	9.9

Table 22. LOO Comparison - Results for Initial Choice - Positive Interaction

Model	elpd_diff	SE_{elpd_diff}
fc_re_ea_support	0.0	0.0
fc_re_ea_study	-15.1	9.9
fc_re_ea_known_before	-20.7	9.3
fc_re_ea_gender	-21.5	10.3
fc_fe_loglength_interactionRHS_rhs05	-24.0	9.3
fc_fe_loglength_interactionRHS_rhs025_gumbel	-24.0	9.3
fc_fe_loglength_interactionRHS_rhs025	-24.2	9.2
fc_fe_loglength_interactionall	-26.5	10.1
fc_re_ea_groupmember	-29.5	9.3
fc_re_ea_travel	-30.2	9.1
fc_re_ea_natio	-30.7	9.8
fc_re_ea_lang	-31.9	9.7
fc_re_ea_freetime	-34.2	8.9
fc_re_ea_popular	-36.2	9.3
fc_fe_wave_interactionRHS_rhs05	-36.8	9.4
fc_fe_wave_interactionRHS_rhs025_gumbel	-36.8	9.5
fc_fe_wave_interactionRHS_rhs025	-37.2	9.3
fc_fe_rhs05_gumbel	-37.2	9.2
fc_fe_plain	-37.2	9.2
fc_fe_rhs05_logreg	-37.2	9.2
fc_fe_rhs025	-37.3	9.2
fc_fe_plain_weaker_prior25	-37.3	9.3
fc_fe_rhs05	-37.3	9.2
fc_fe_rhs075	-37.3	9.2
fc_fe_L1_interactionRHS_rhs025	-37.3	9.2
fc_fe_L1_interactionRHS_rhs05	-37.4	9.2
fc_fe_L1_interactionRHS_rhs025_gumbel	-37.5	9.3
fc_fe_L1_interactionall	-39.3	10.1
fc_fe_wave_interactionall	-42.6	11.6

Table 23. LOO Comparison - Results for Subsequent Choice - Positive Interaction

Model	elpd_diff	SE_{elpd_diff}
oc_fe_choicexwave_interactionRHS_rhs05	0.0	0.0
oc_fe_choicexwave_interactionRHS_rhs025	-0.8	0.3
oc_fe_choicexwave_interactionRHS_rhs025_gumbel	-0.8	0.3
oc_fe_choice_interactionRHS_rhs05	-2.4	2.9
oc_fe_choice_interactionRHS_rhs025	-2.4	2.9
oc_fe_choice_interactionRHS_rhs025_gumbel	-2.7	2.9
oc_fe_choice_interactionall	-13.3	6.8
oc_fe_choiceL1waveL1_interactionRHS_rhs025	-13.7	9.4
oc_fe_choiceL1waveL1_interactionRHS_rhs05	-13.8	9.4
oc_fe_choiceL1waveL1_interactionRHS_rhs025_gumbel	-13.9	9.4
oc_fe_loglength_interact	-15.1	16.8
oc_fe_loglength_interactionRHS_rhs025	-15.6	15.5
oc_fe_loglength_interactionRHS_rhs025_gumbel	-15.6	15.5
oc_fe_loglength_interactionRHS_rhs05	-15.8	15.5
oc_fe_wave_choice_interactionall	-20.7	10.4
oc_fe_waveL1_choiceL1_interactionall	-23.2	11.6
oc_fe_choiceL1_interactionRHS_rhs025_gumbel	-26.1	8.3
oc_fe_choiceL1_interactionRHS_rhs05	-26.1	8.3
oc_fe_choiceL1_interactionRHS_rhs025	-26.1	8.3
oc_fe_choiceL1_interactionall	-31.6	9.6
oc_re_ea_support	-41.0	14.1
oc_re_aa_percievedgroup	-42.0	15.7
oc_re_ea_groupmember	-56.5	14.4
oc_re_aa_support	-59.8	14.3
oc_fe_waveL1_interactionRHS_rhs05	-61.6	13.4
oc_fe_waveL1_interactionRHS_rhs025_gumbel	-62.0	13.4
oc_fe_waveL1_interactionRHS_rhs025	-62.0	13.4
oc_fe_waveL1_interactionall	-64.6	14.3
oc_re_aa_gender	-65.5	13.6
oc_re_aa_name	-66.2	14.0
oc_re_ea_knownbefore	-68.8	13.3
oc_re_ea_pop	-69.5	13.2
oc_re_ea_gender	-70.2	13.2
oc_re_ea_freetime	-70.8	12.9
oc_re_aa_freetime	-72.6	12.9
oc_re_aa_lang	-74.1	12.9
oc_re_aa_knownbefore	-74.4	12.9
oc_fe_wave_interactionRHS_rhs05	-74.5	12.4
oc_re_ea_study	-74.5	12.9
oc_re_ea_travel	-74.6	12.9
oc_re_aa_study	-74.8	12.9
oc_fe_wave_interactionRHS_rhs025	-74.9	12.5
oc_fe_wave_interactionRHS_rhs025_gumbel	-75.0	12.4
oc_fe_rhs025	-75.1	12.7
oc_fe_rhs05_logreg	-75.2	12.7
oc_fe_rhs05	-75.2	12.7
oc_fe_rhs075	-75.3	12.7
oc_re_ea_lang	-75.3	12.7
oc_fe_rhs05_gumbel	-75.4	12.7
oc_re_aa_travel	-75.5	12.7
oc_re_aa_natio	-75.6	12.8
oc_fe_plain	-75.7	12.7
oc_fe_plain_weaker_prior25	-75.8	12.7
oc_re_ea_natio	-76.0	12.7
oc_re_aa_popular	-76.5	12.7
oc_fe_wave_interactionall	-82.4	14.8

11 LOO Comparison Results with Correction

Due to the widespread use of our model comparison, as demonstrated by McLatchie & Vehtari[13], we need to correct for improved model performance based on randomness and the biased forward selection approach. To do so, we used the suggested parsimonious correction based on order-statistics, as suggested in the paper, as predictive projection is not developed yet and not trivial for our statistical model. The results of the model comparison with correction are shown in the plot below. For all of them, we considered the models to be a relevant extension, with better model fit if they surpassed the upper boundary (indicated in red). The model without error bars is the reference model used for the correction.

Figure 9. Distribution of elpd-loo after correction - Initial Choices - Friendship

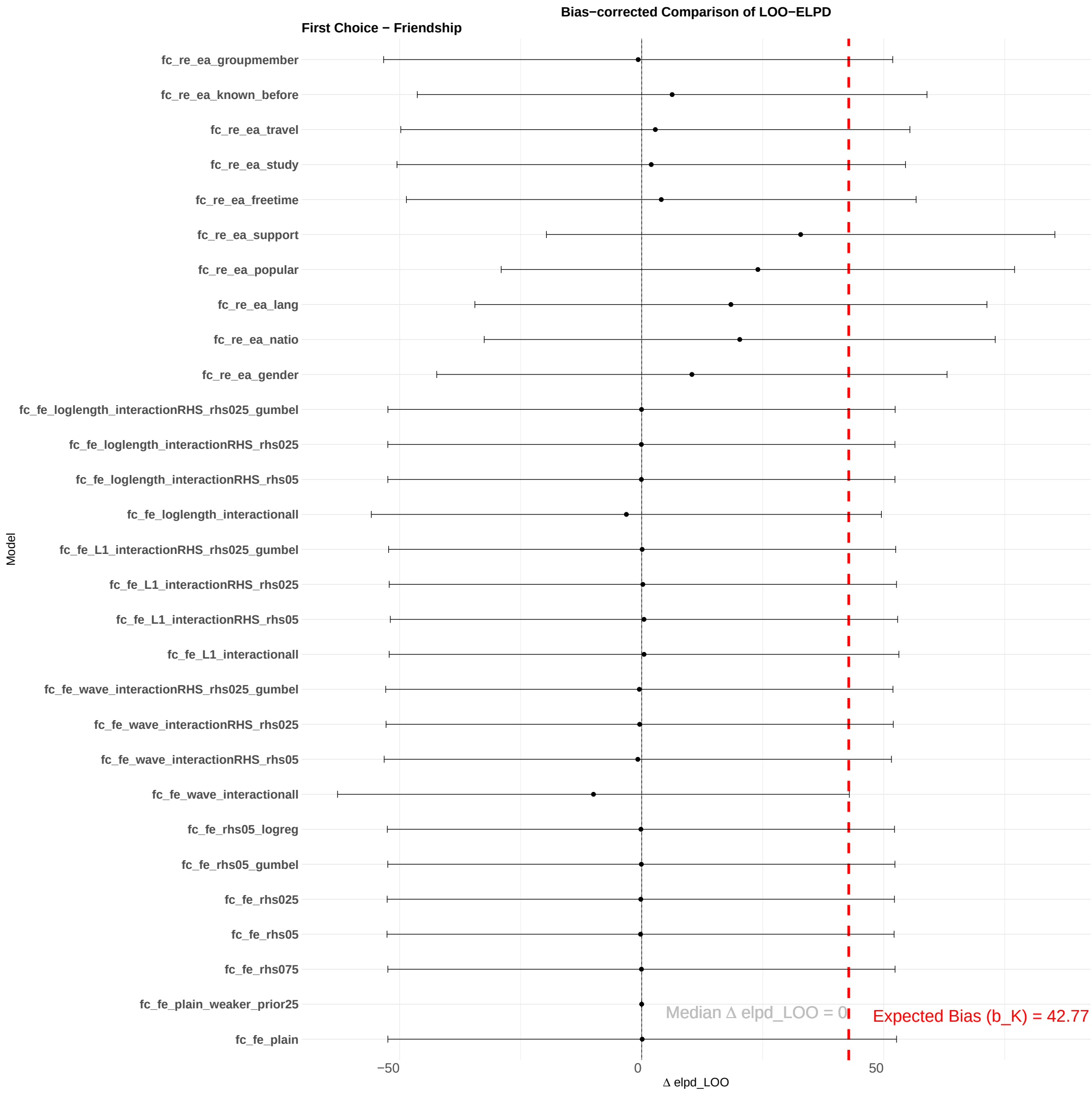

Figure 10. Distribution of elpd-loo after correction - Initial Choices - Positive Interaction

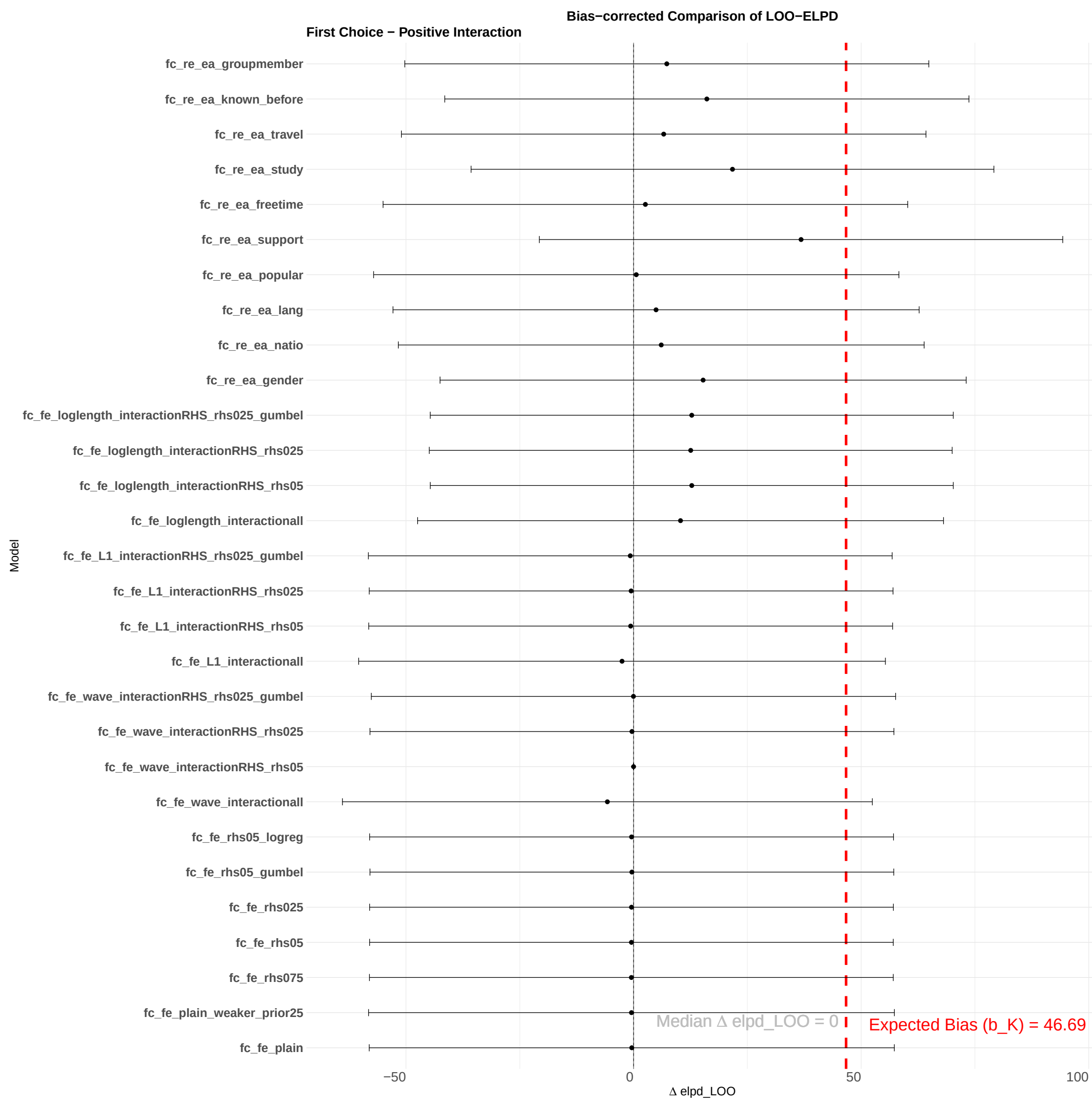

Figure 11. Distribution of elpd-loo after correction - Subsequent Choices - Friendship

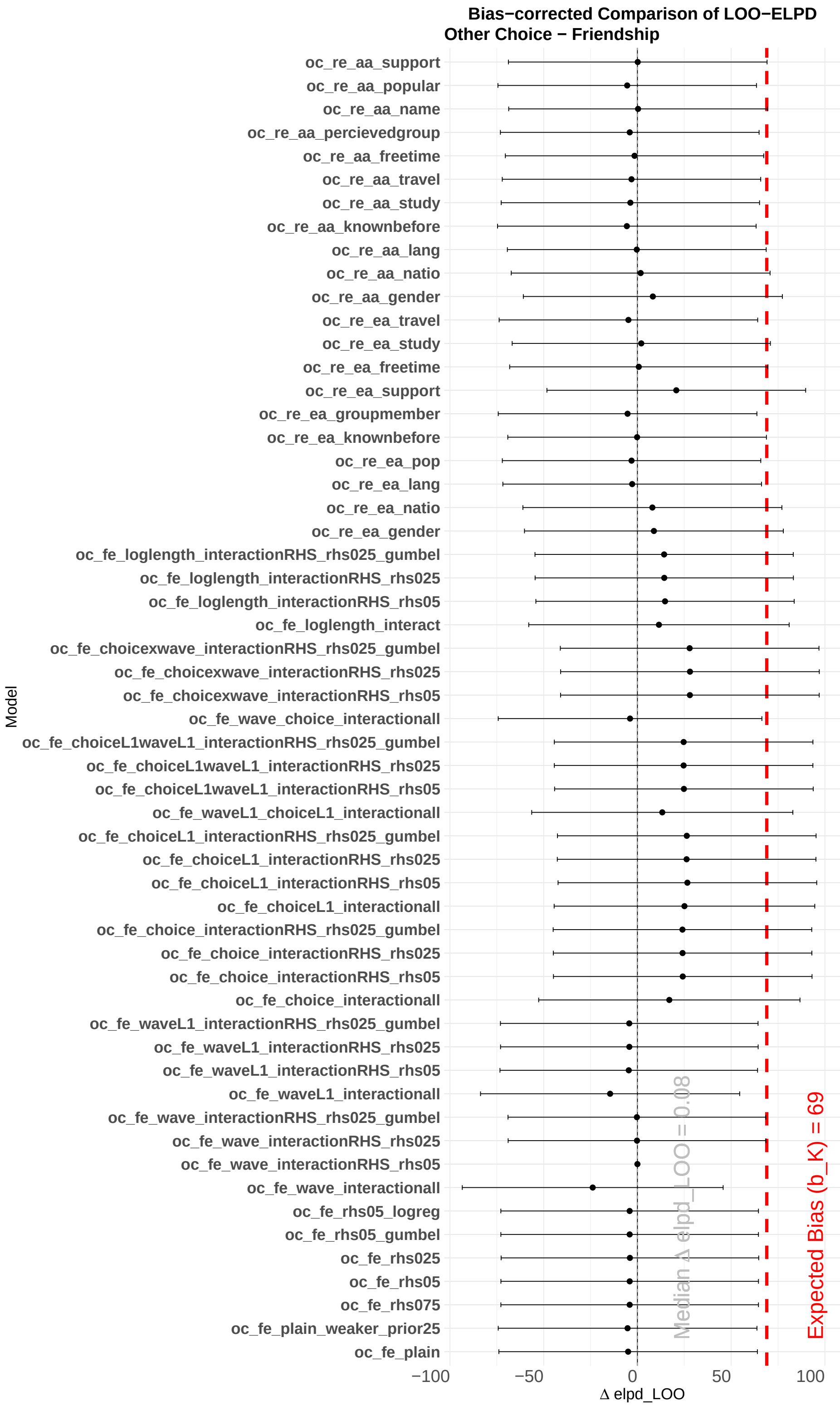

Figure 12. Distribution of elpd-loo after correction - Subsequent Choices - Positive Interaction

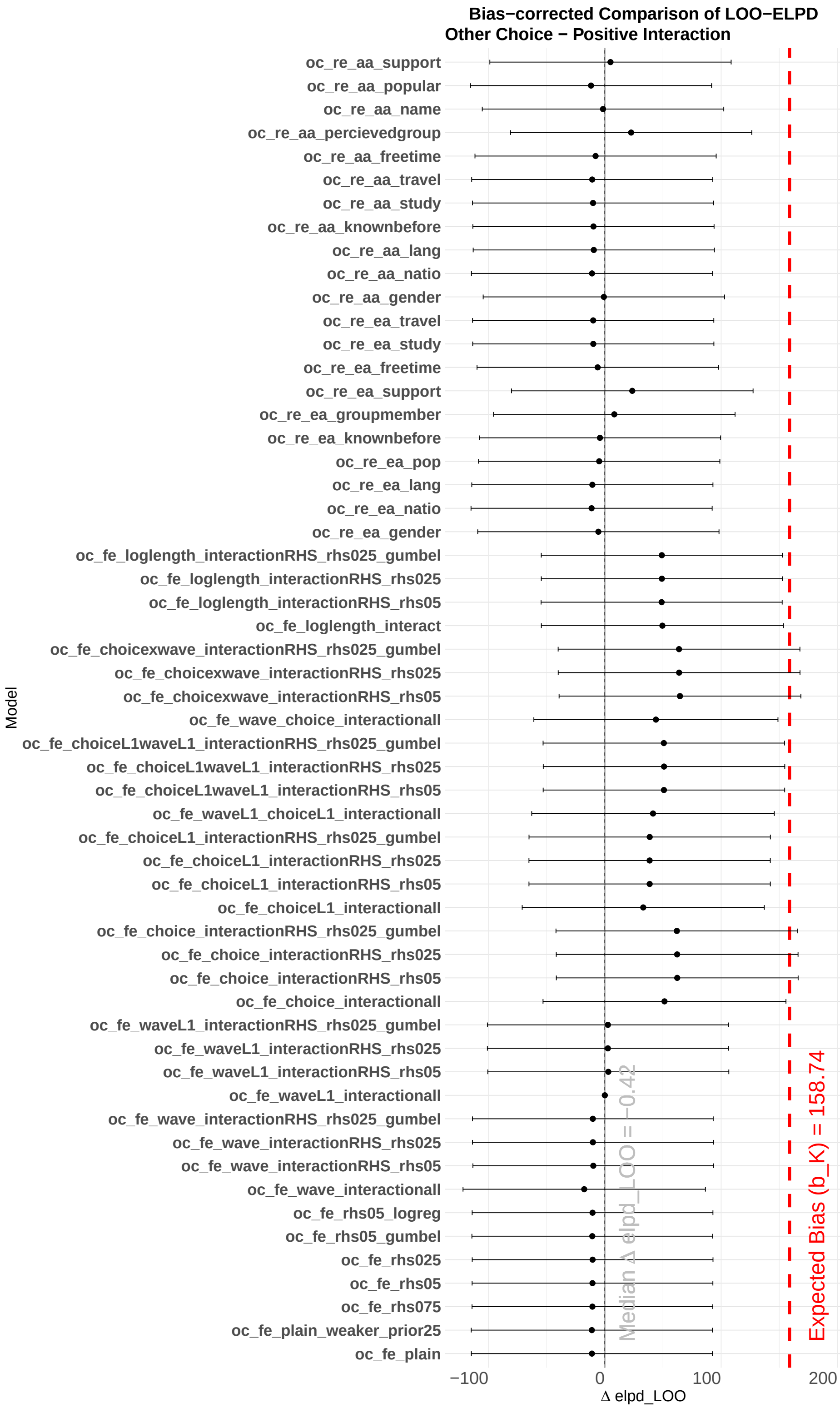

12 Usage of AI

Table 24. Use of AI in the Manuscript

AI-Based tool	Use Case	Scope/Remark
Gemini 2.5 Flash<br>Last accessed:<br>June 15, 2026	Correction of grammatical and stylistic mistakes<br>Suggestions for stylistic phrasings<br>Code completion and Code review	Entire work
Grammarly<br>Last accessed:<br>June 15, 2026	Correction of grammatical and stylistic mistakes	Entire work / Premium plan with Overleaf integration.
GitHub Copilot<br>Last accessed:<br>June 15, 2026	Code completion and Code review	Generation of code for results in R.

13 Initial Choice - Friendship

The following plots the results of each model for the initial nomination of friendship sequences. The models are grouped (especially for the interaction models) so that similar configurations are together, and the effects represent the same abstract meaning.

13.1 Initial Choice - Friendship - Weaker Prior

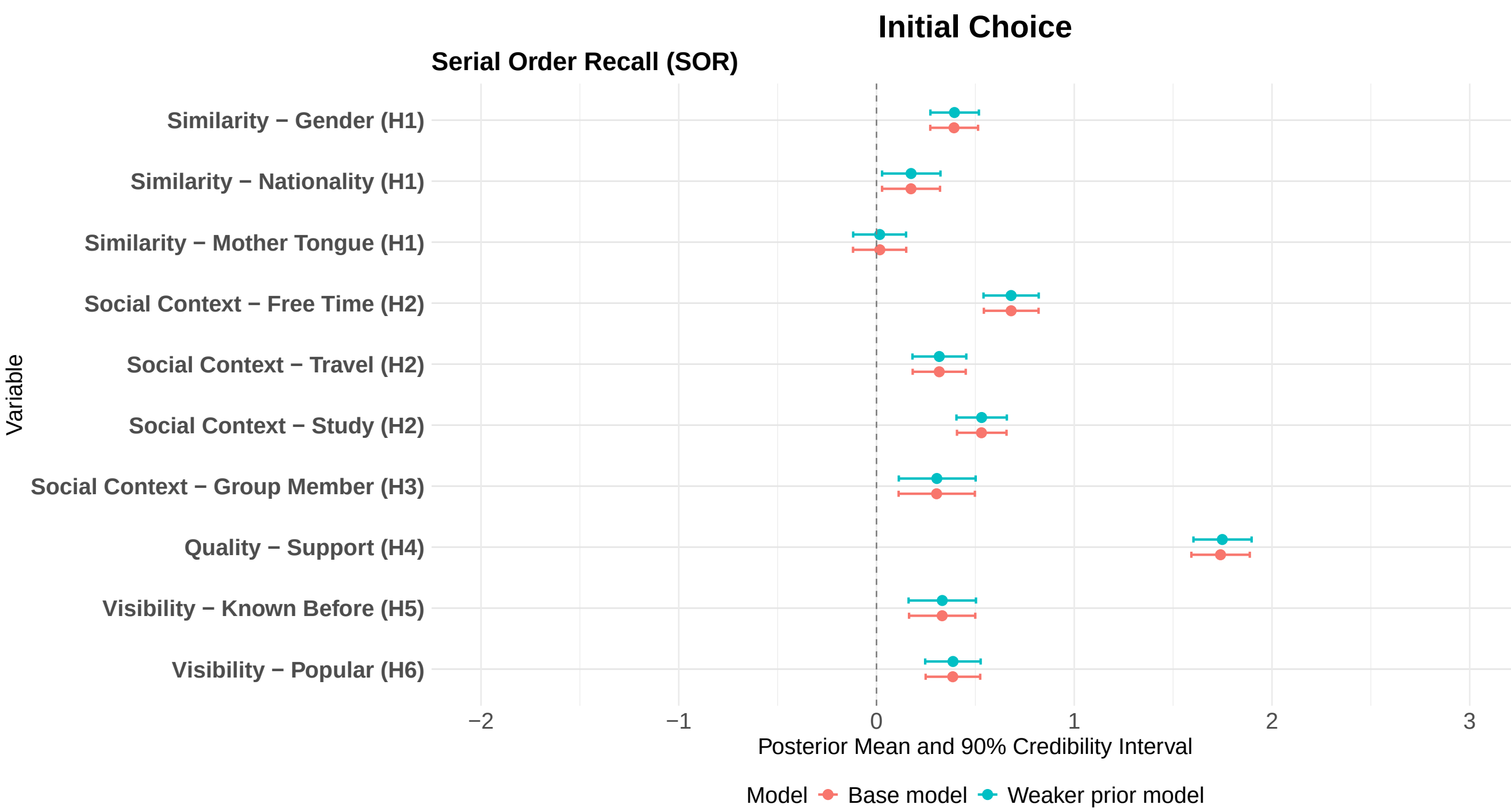

Figure 13. Comparing the base model with an alternative configuration with a weaker prior.

13.2 Initial Choice - Friendship - Regularized Horseshoe Prior (RHS)

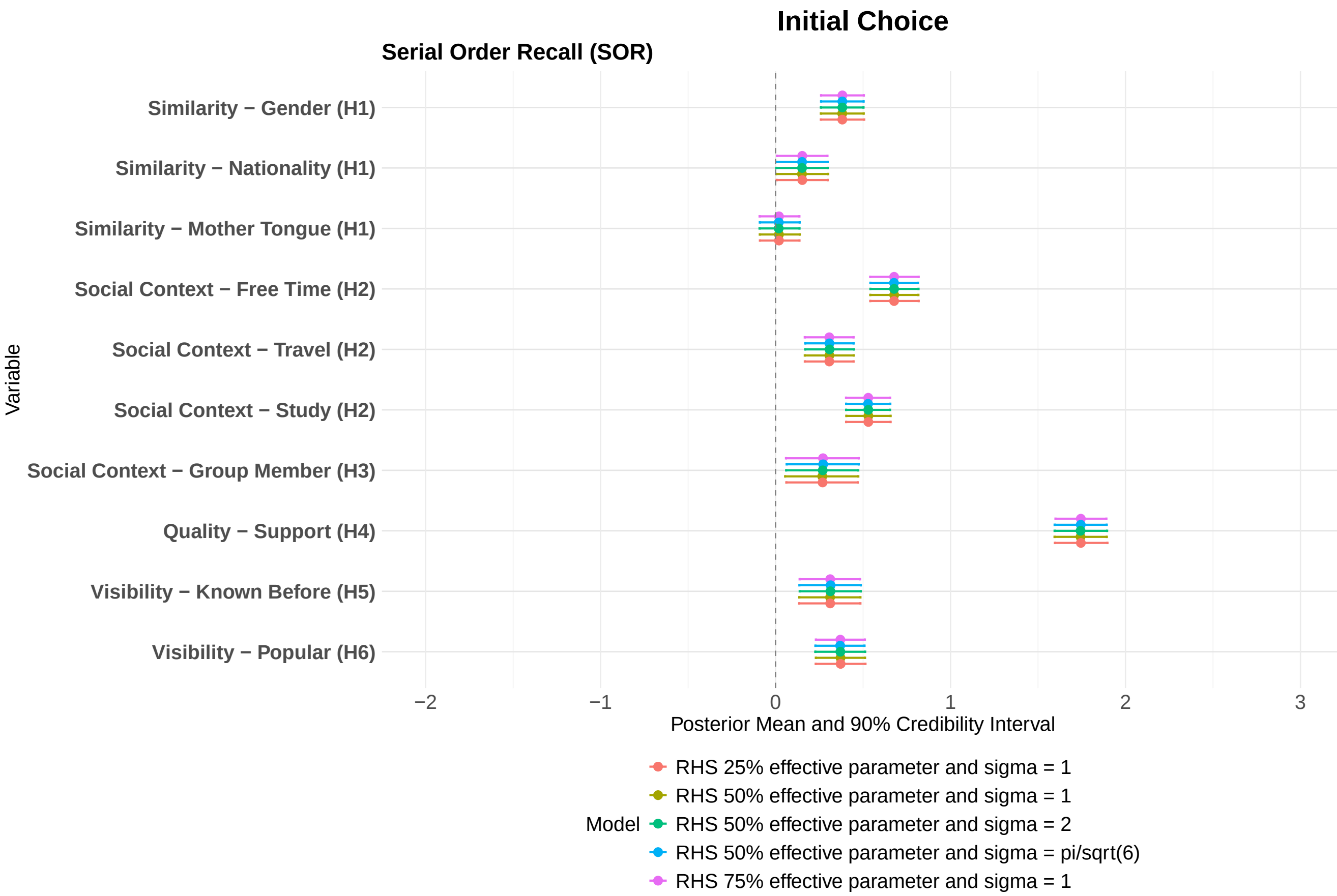

Figure 14. Comparing the base model with an alternative configuration with a regularized horseshoe prior under different configurations.

13.3 Initial Choice - Friendship - Time Interaction

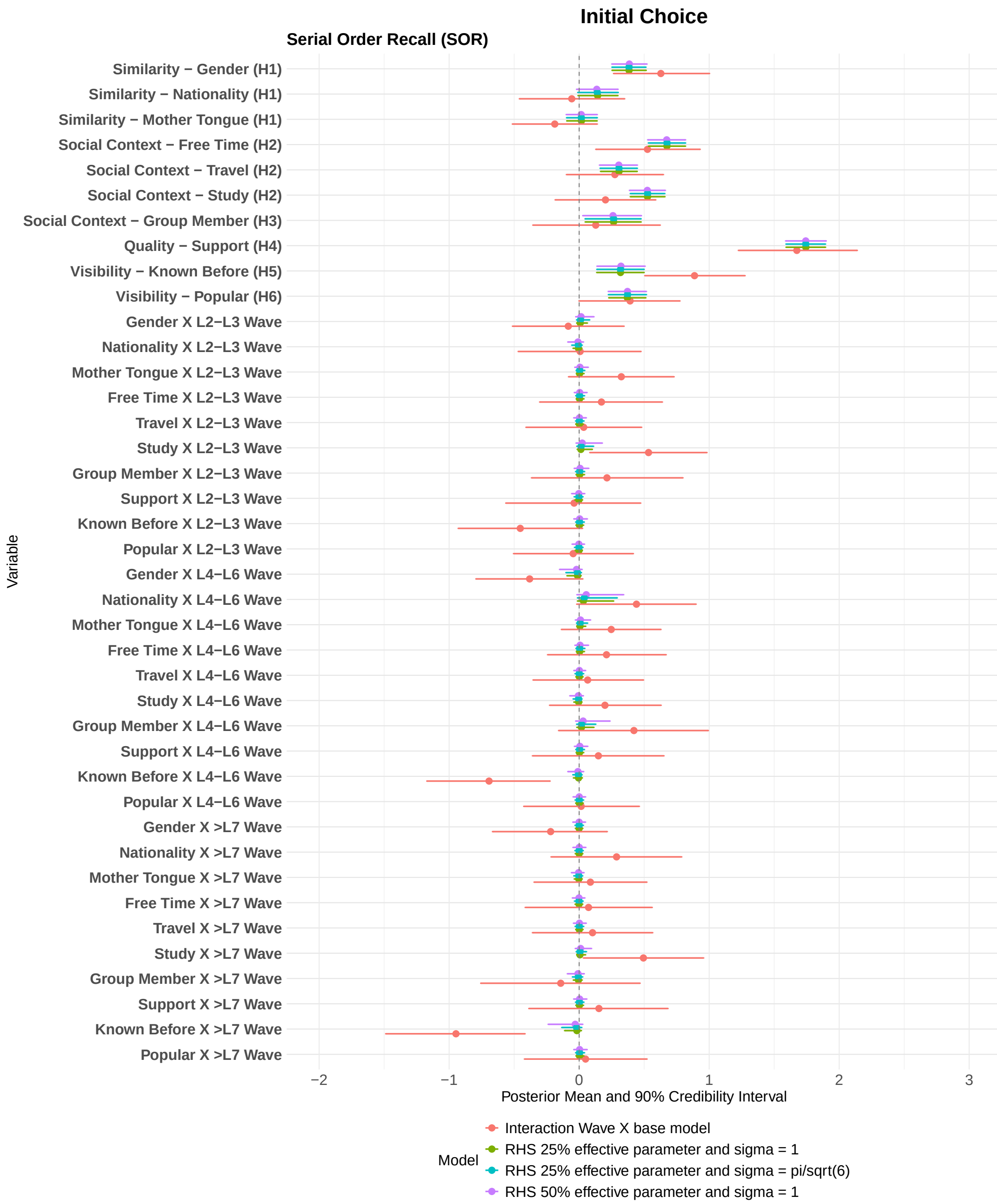

Figure 15. Comparing the base model with alternative models including time-related interactions.

13.4 Initial Choice - Friendship - Time Interaction with Focus on First Wave

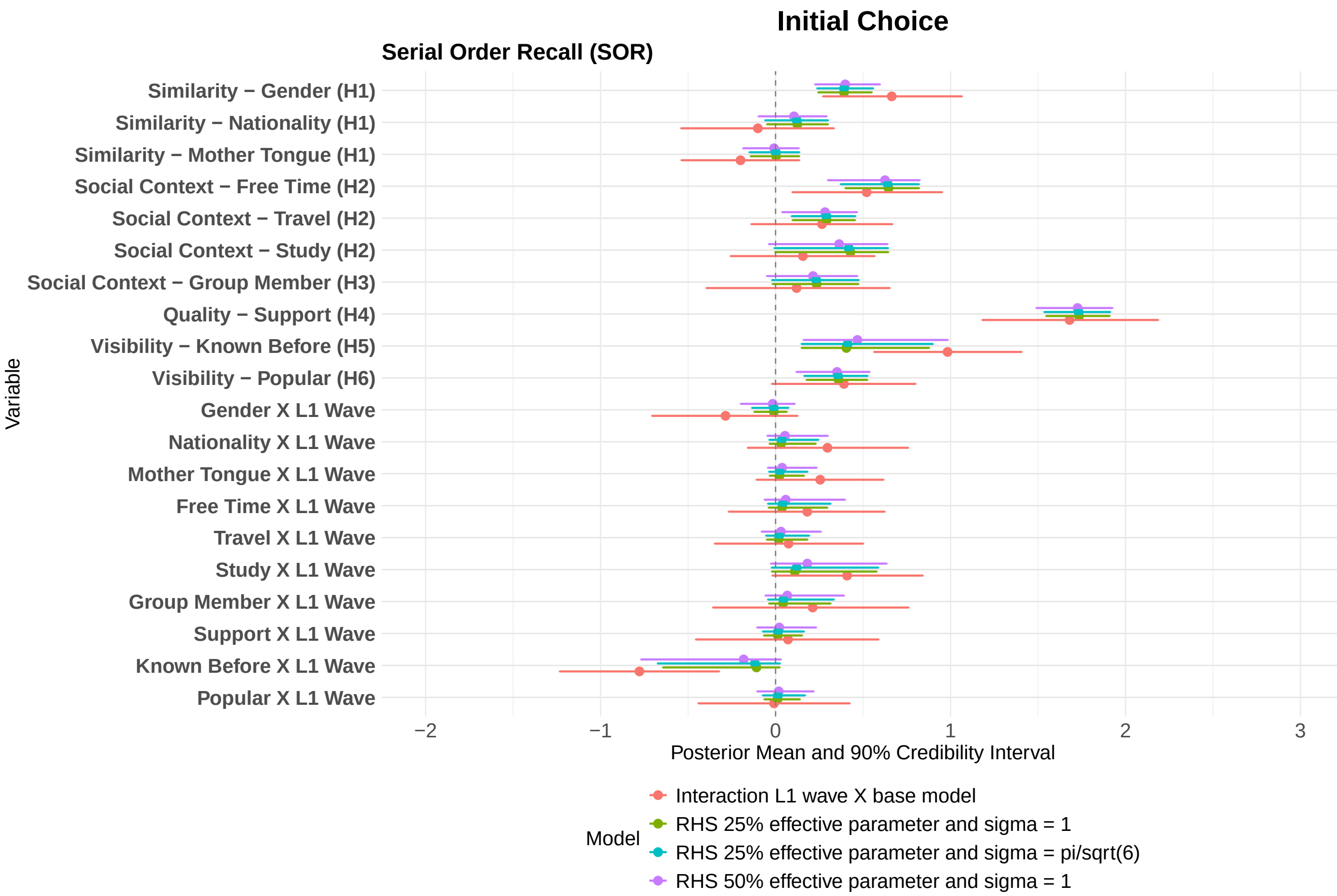

Figure 16. Comparing the base model with alternative models including time-related interactions - focusing only on Wave 1.

13.5 Initial Choice - Friendship - Sequence Length Interaction

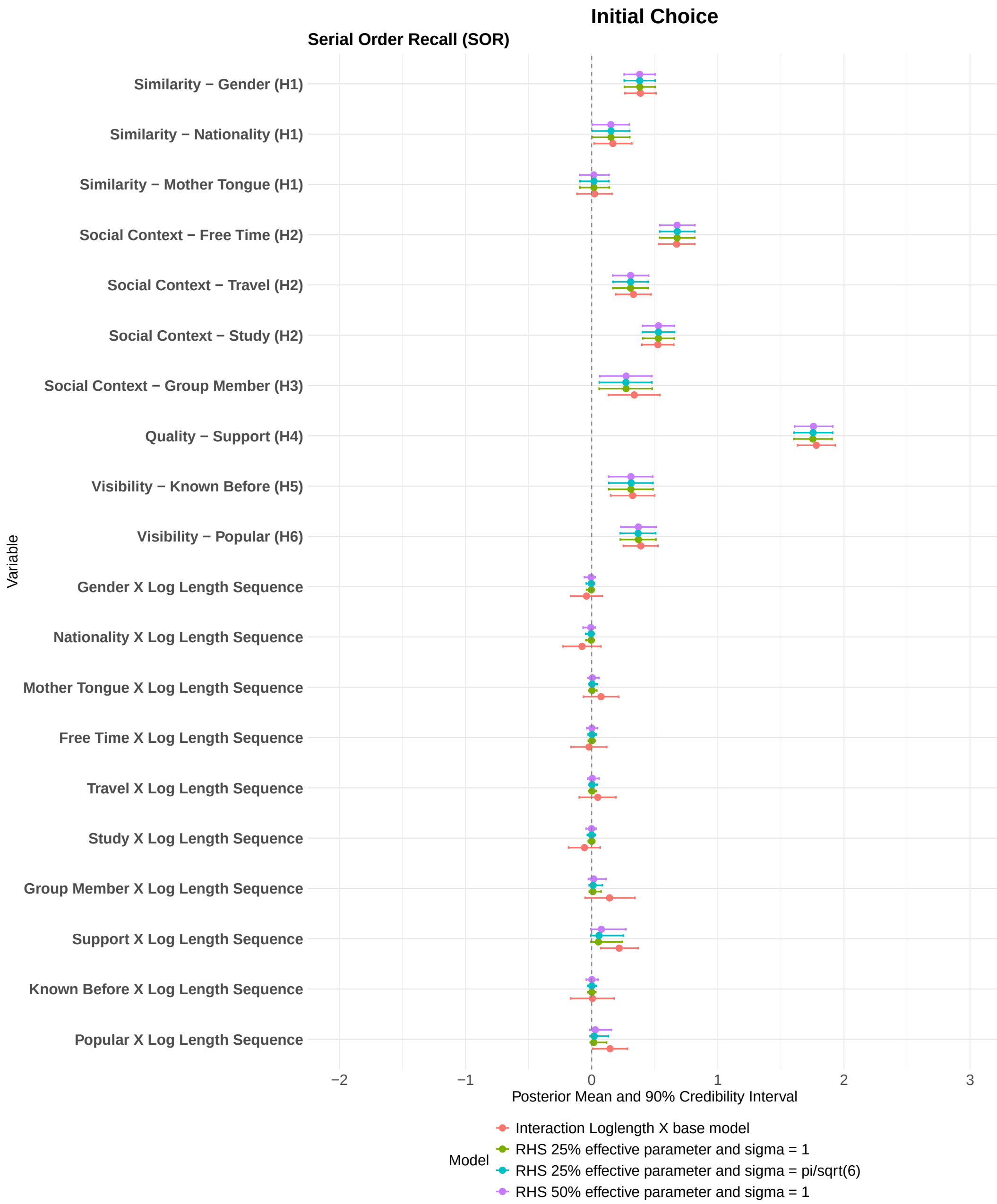

Figure 17. Comparing the base model with alternative models, including sequence length-related interactions.

13.6 Initial Choice - Friendship - Random Slope for Individuals

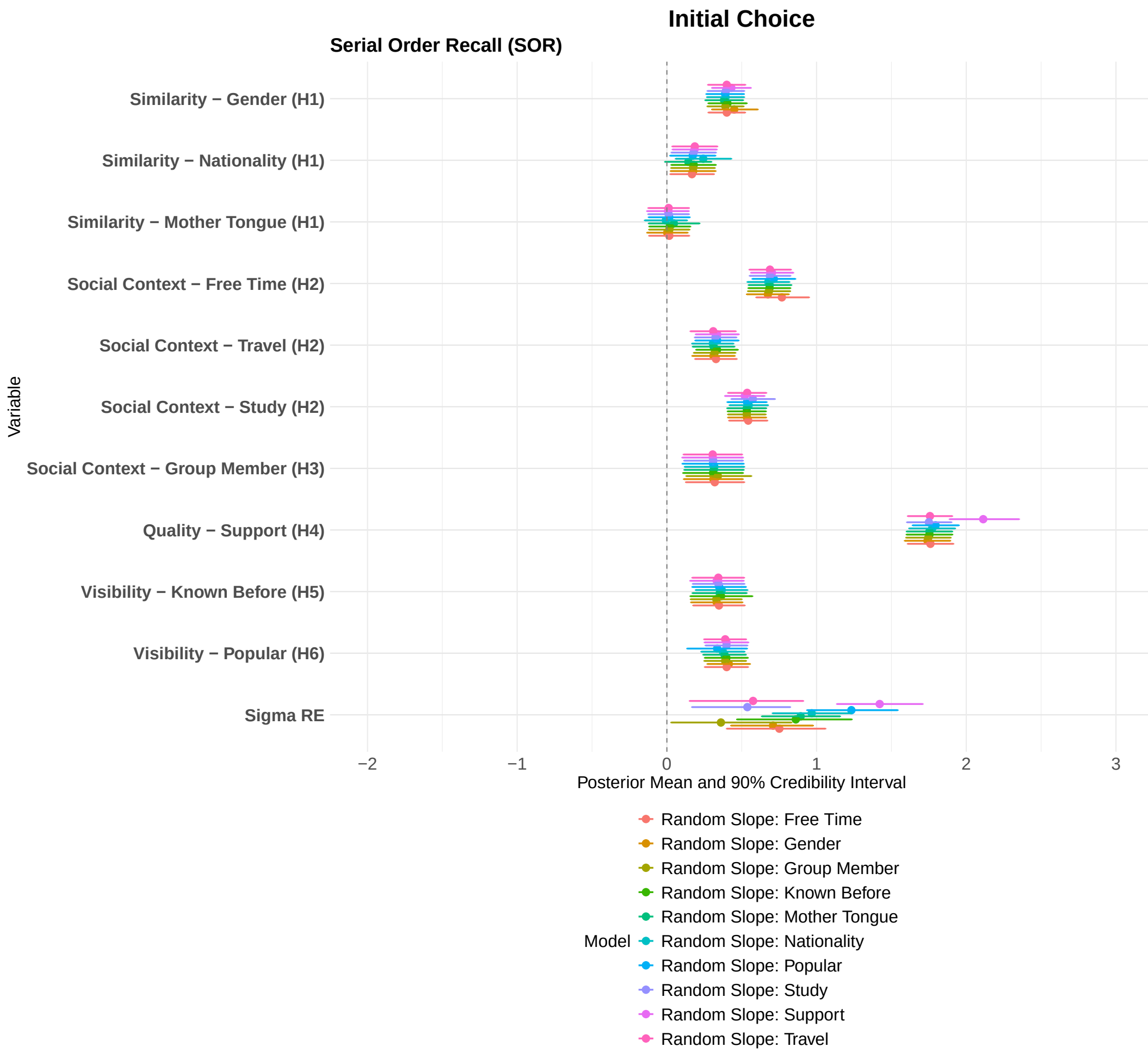

Figure 18. Comparing the base model with alternative models, including a random slope for each predictor separately based on individuals.

13.7 Initial Choice - Friendship - MICE imputation

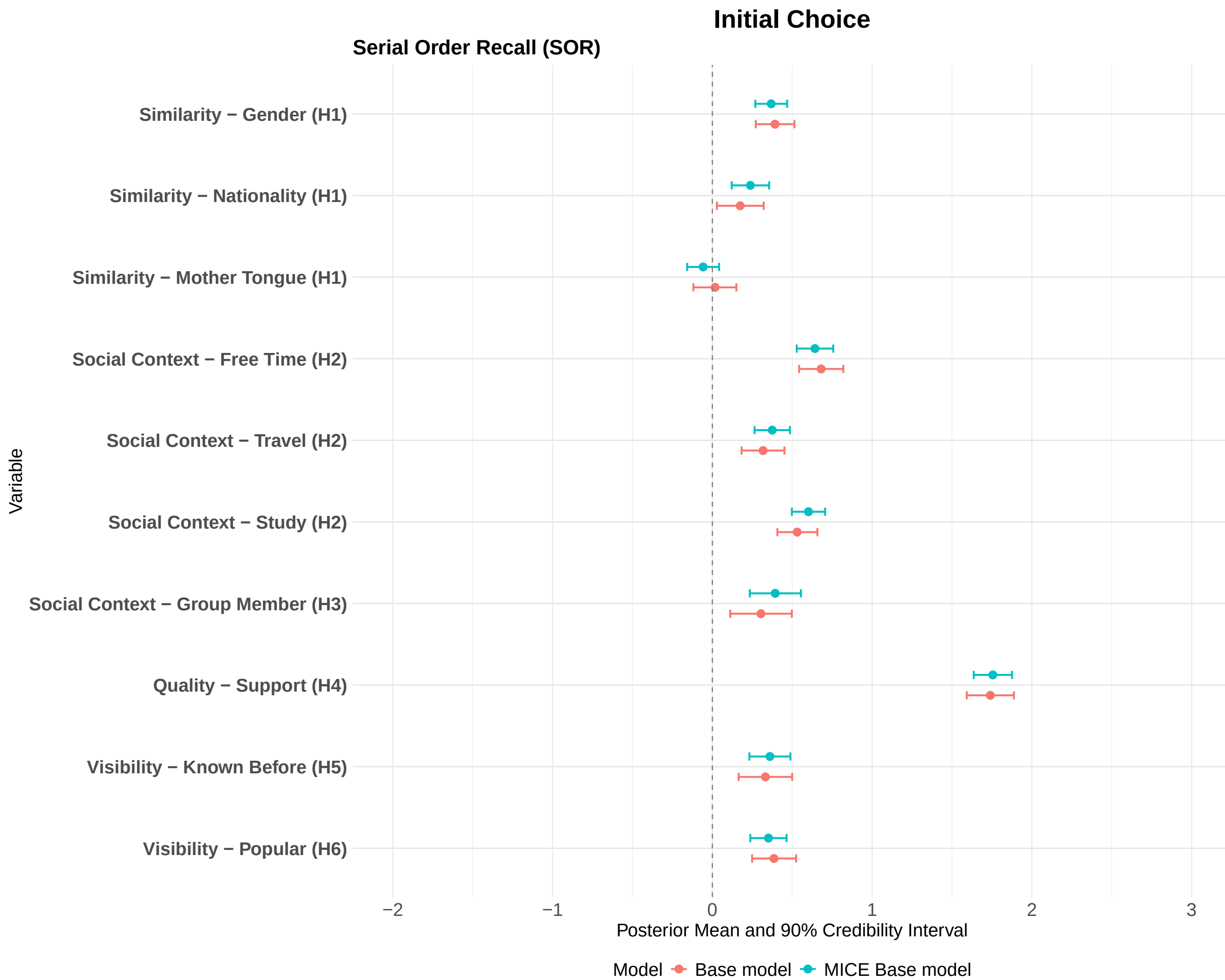

Figure 19. Comparing the base model with an alternative model using the mice imputed datasets.

13.8 Initial Choice - Friendship - Reduced Choice set

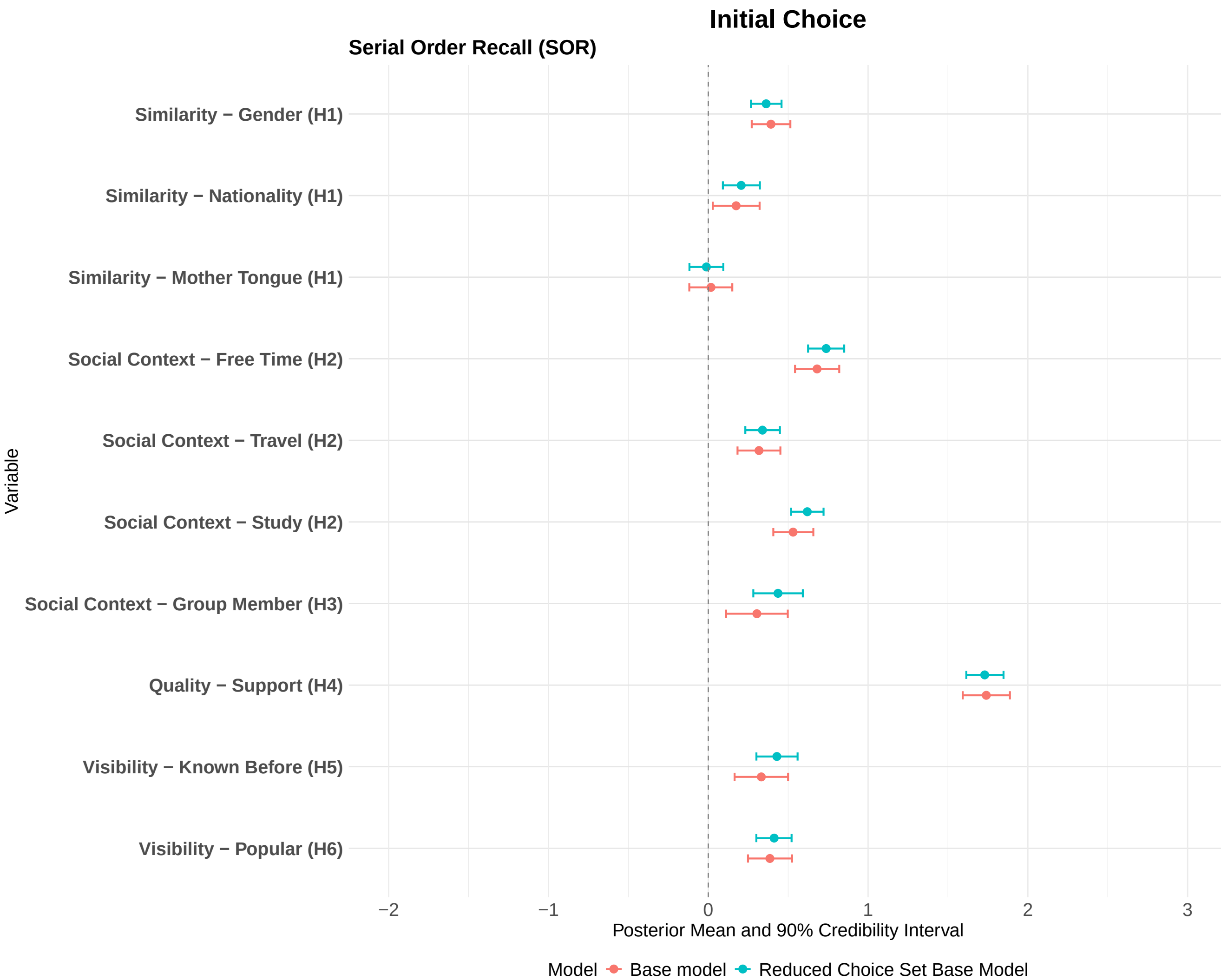

Figure 20. Comparing the base model with an alternative model using the reduced choice sets.

13.9 Initial Choice - Friendship - Explorative Robustness Operationalisations

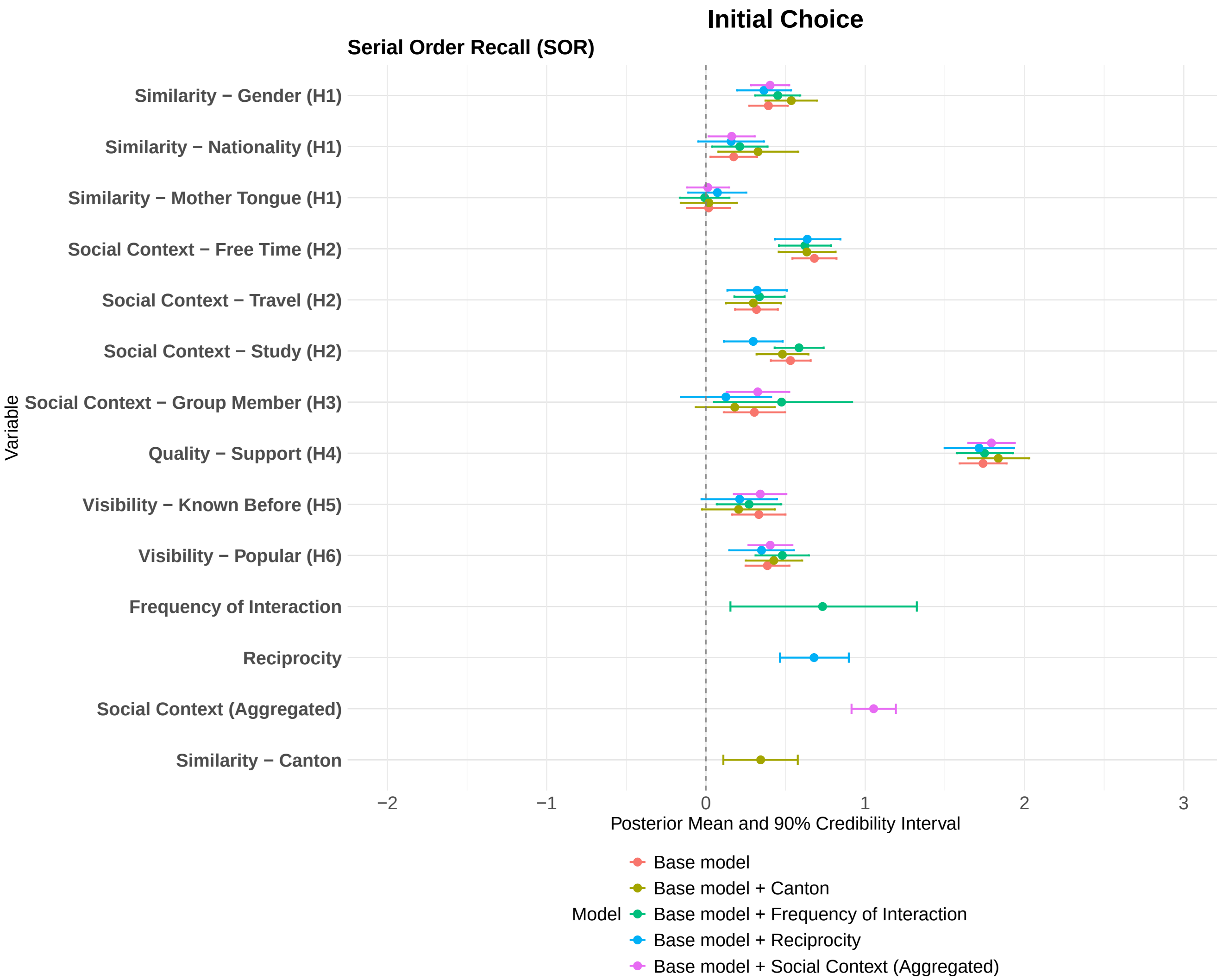

Figure 21. Comparing the base model with alternative models, using different Operationalisations and new predictors.

14 Initial Choice - Positive Interaction

The following plots the results of each model for the initial nomination of positive interaction sequences. The models are grouped (especially for the interaction models) so that similar configurations are together, and the effects represent the same abstract meaning.

14.1 Initial Choice - Positive Interaction - Weak Prior

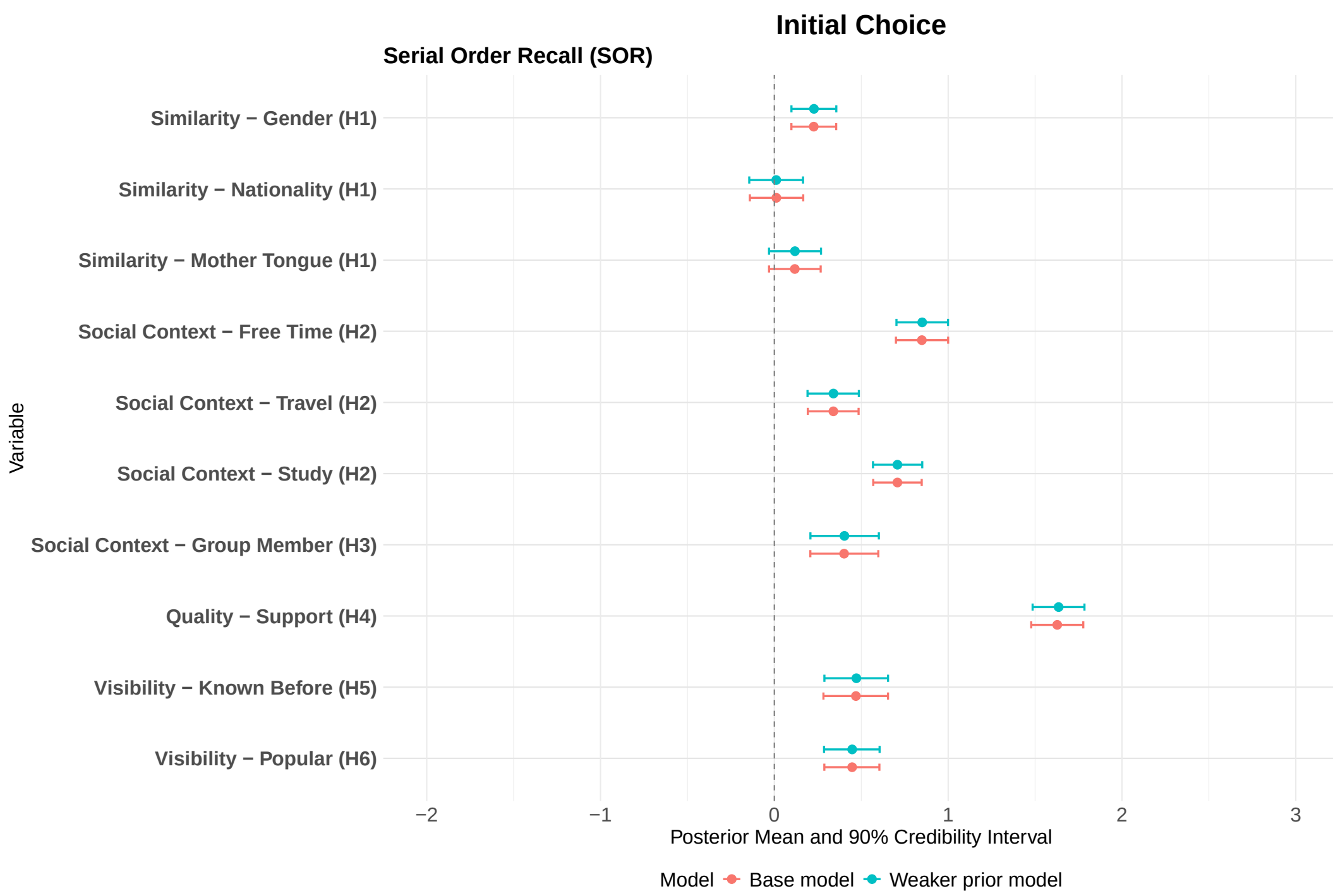

Figure 22. Comparing the base model with an alternative configuration with a weaker prior.

14.2 Initial Choice - Positive Interaction - Regularized Horseshoe Prior (RHS)

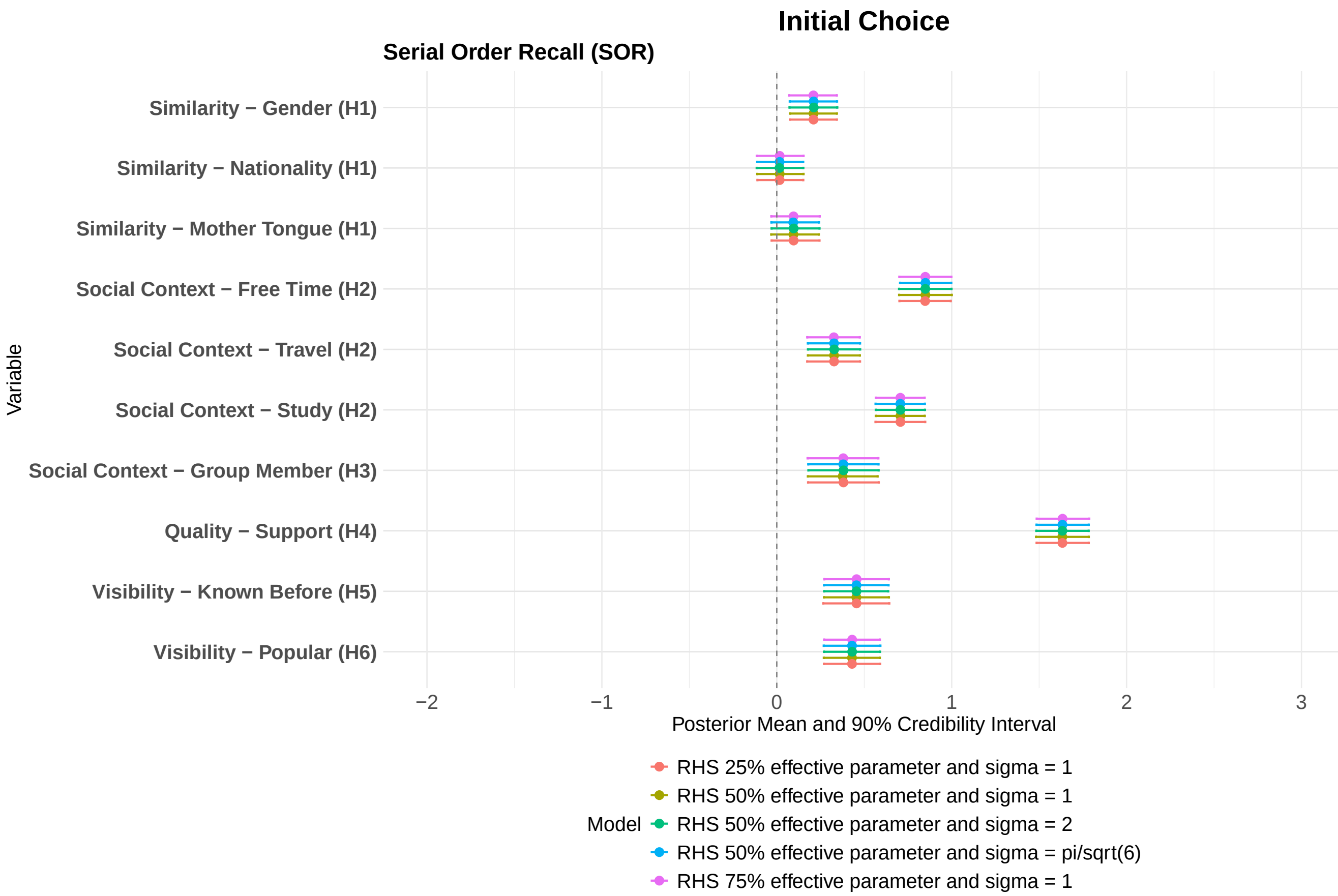

Figure 23. Comparing the base model with an alternative configuration with a regularized horseshoe prior under different configurations.

14.3 Initial Choice - Positive Interaction - Time Interaction

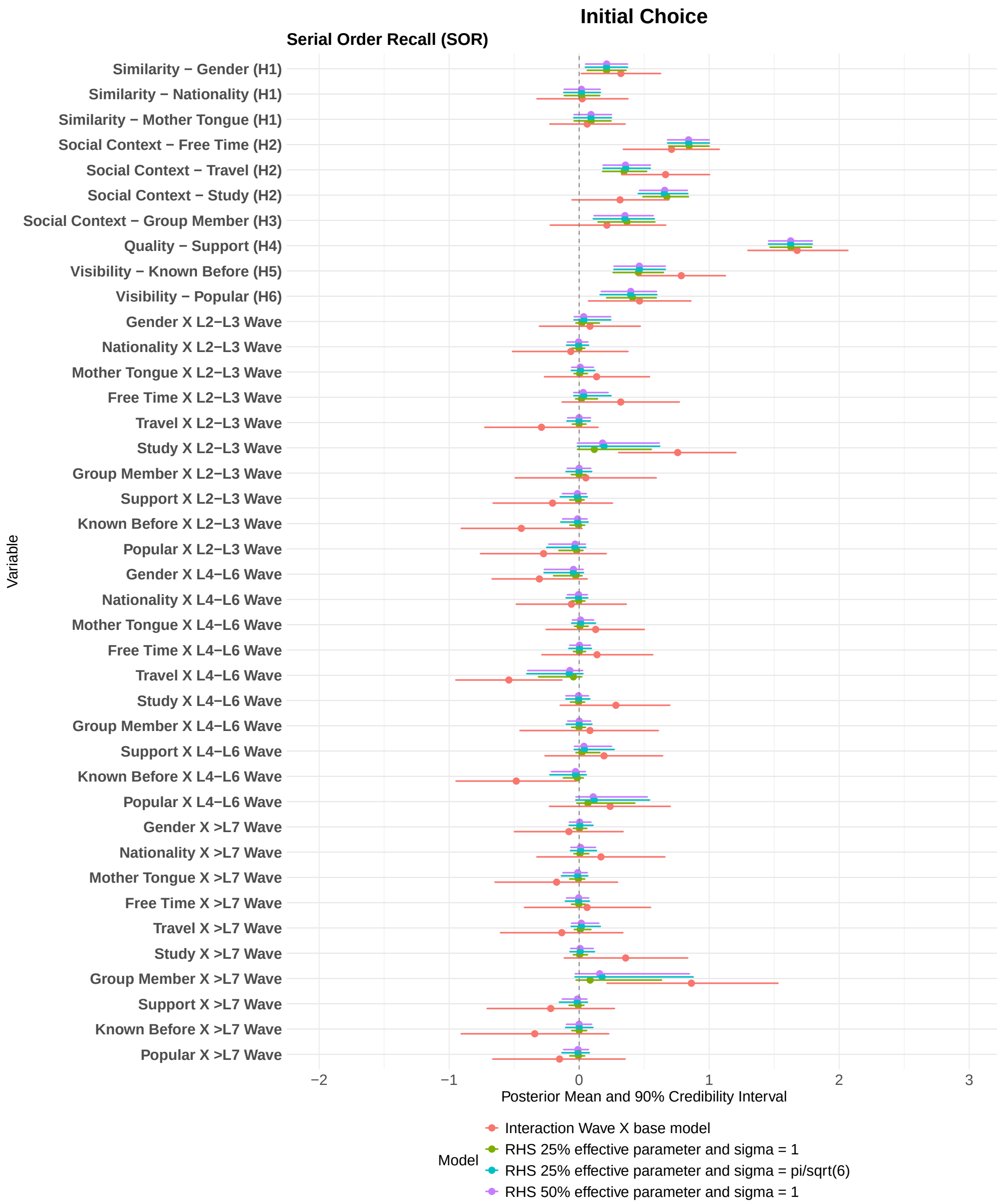

Figure 24. Comparing the base model with alternative models including time-related interactions.

14.4 Initial Choice - Positive Interaction - Time Interaction with Focus on First Wave

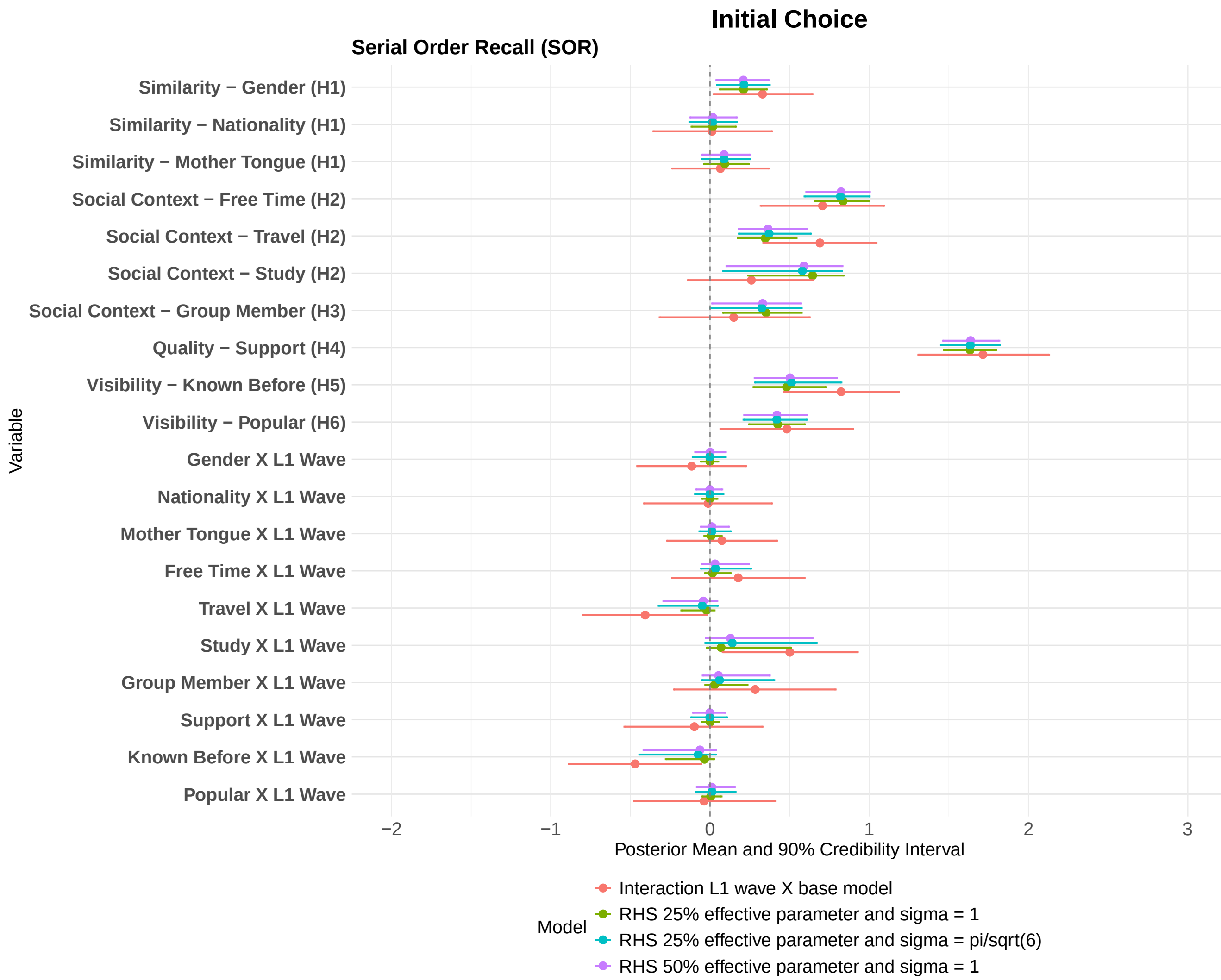

Figure 25. Comparing the base model with alternative models including time-related interactions - focusing only on Wave 1.

14.5 Initial Choice - Positive Interaction - Sequence Length Interaction

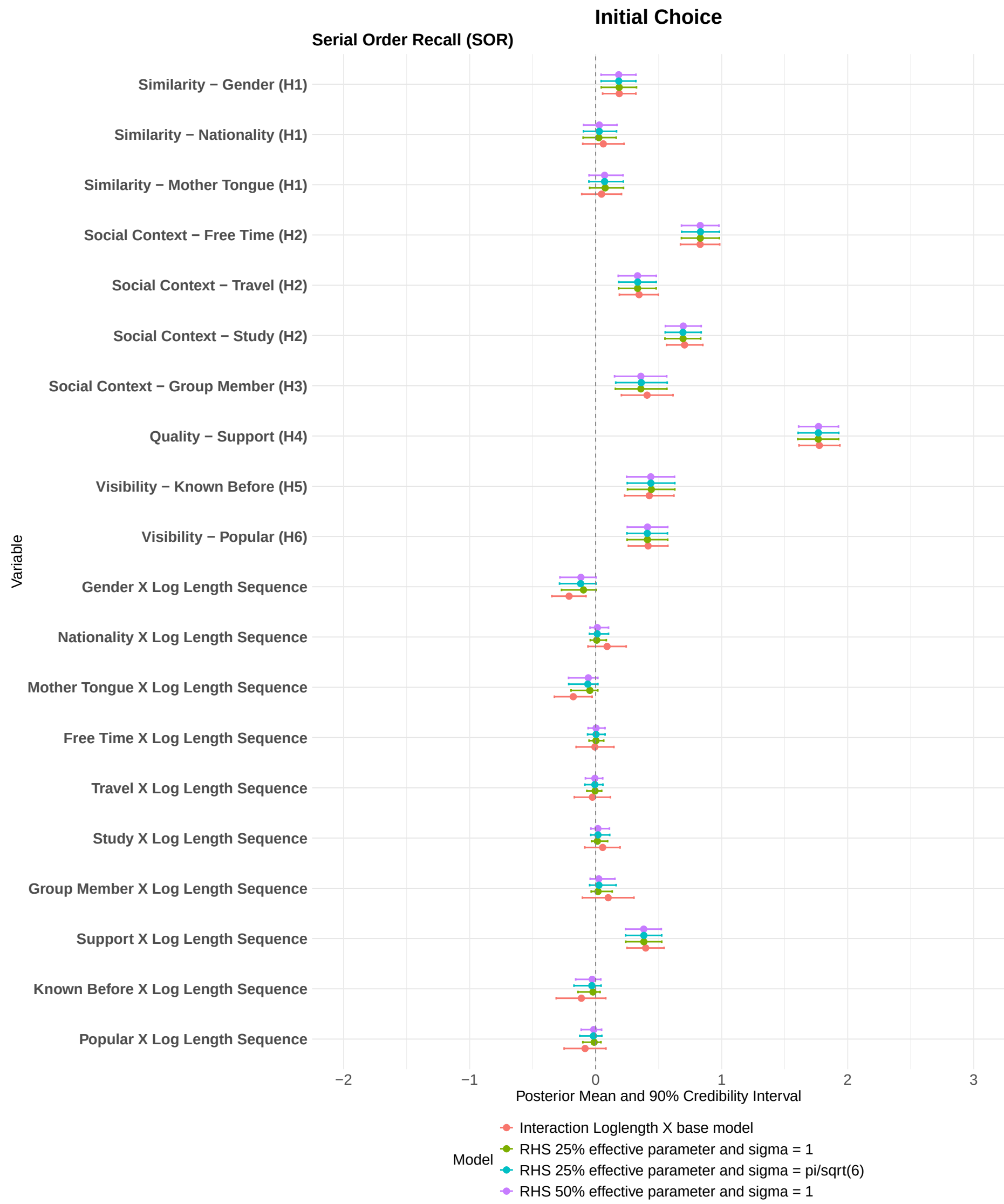

Figure 26. Comparing the base model with alternative models, including sequence length-related interactions.

14.6 Initial Choice - Positive Interaction - Random Slope for Individuals

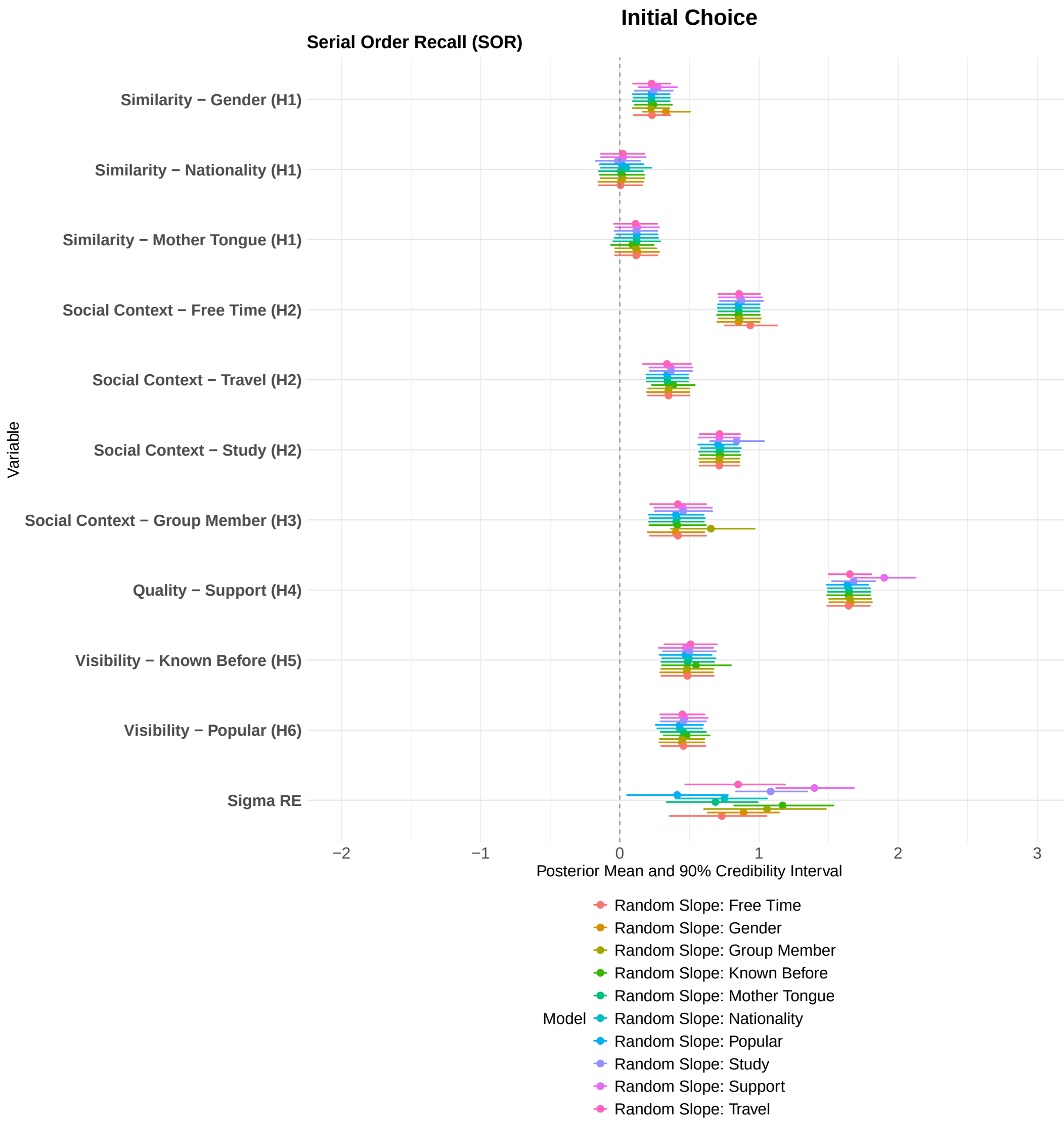

Figure 27. Comparing the base model with alternative models, including a random slope for each predictor separately based on individuals.

14.7 Initial Choice - Positive Interaction - MICE imputation

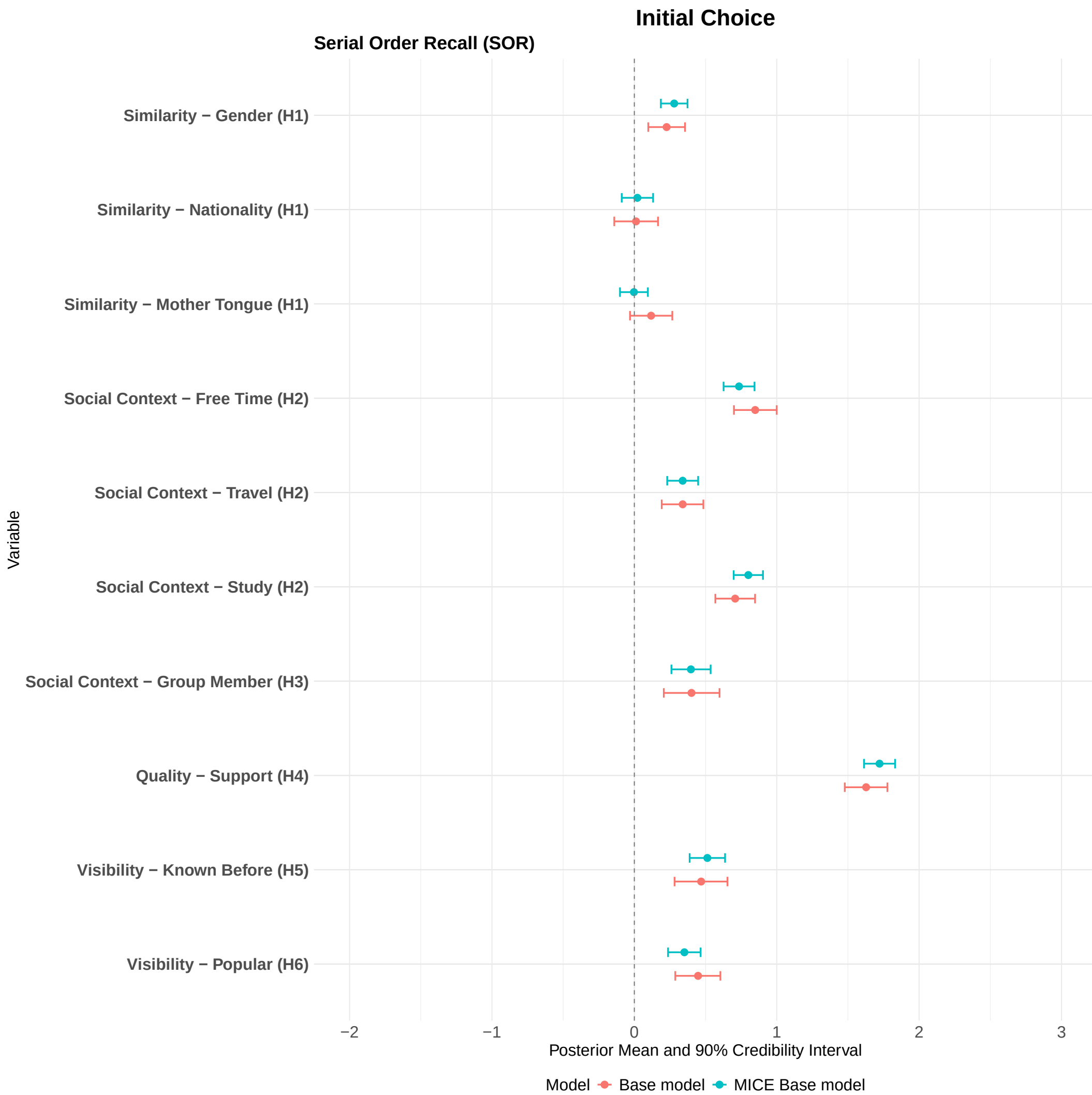

Figure 28. Comparing the base model with an alternative model using the mice imputed datasets.

14.8 Initial Choice - Positive Interaction - Reduced Choice set

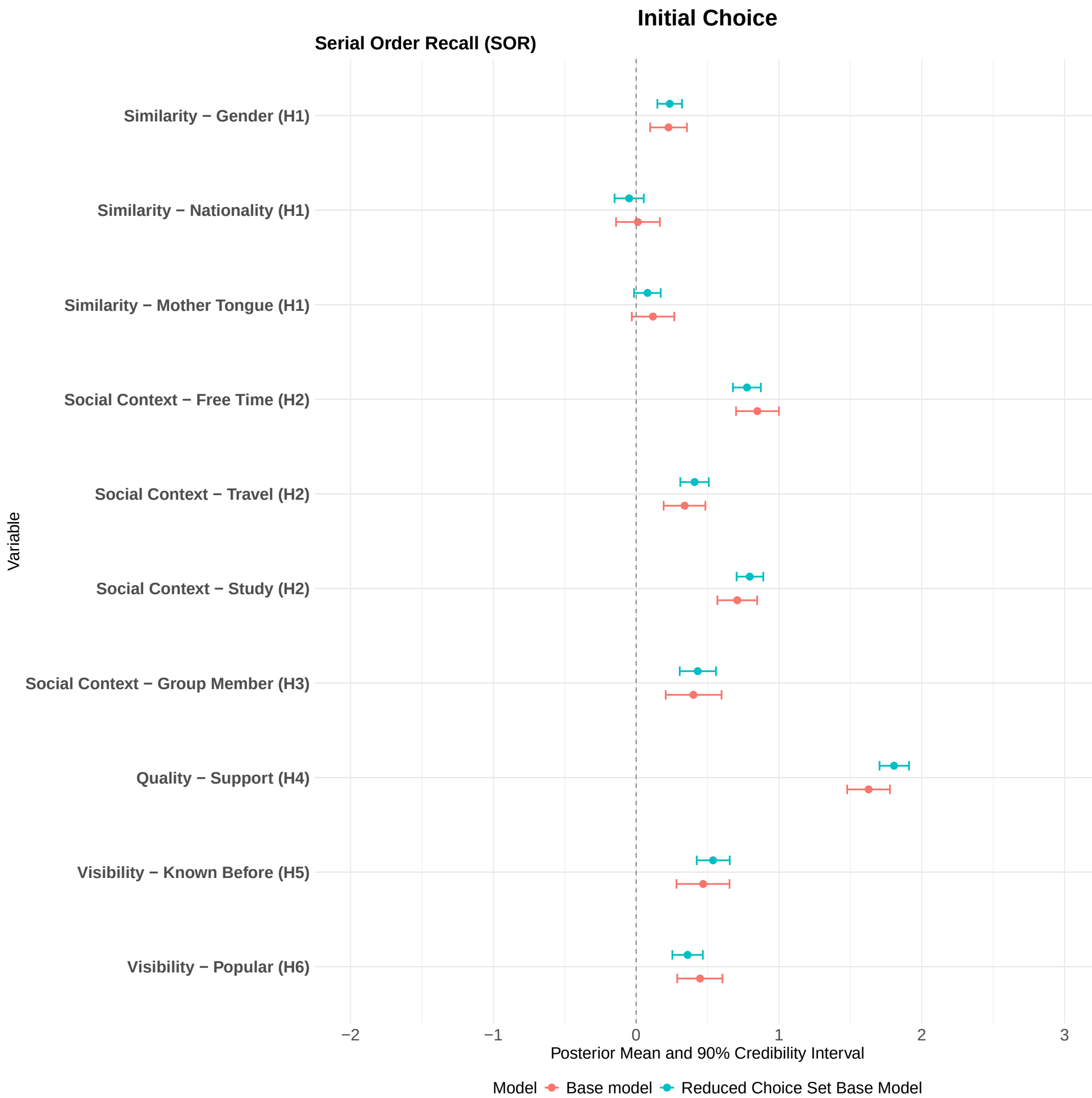

Figure 29. Comparing the base model with an alternative model using the reduced choice sets.

14.9 Initial Choice - Positive Interaction - Explorative Robustness Operationalisations

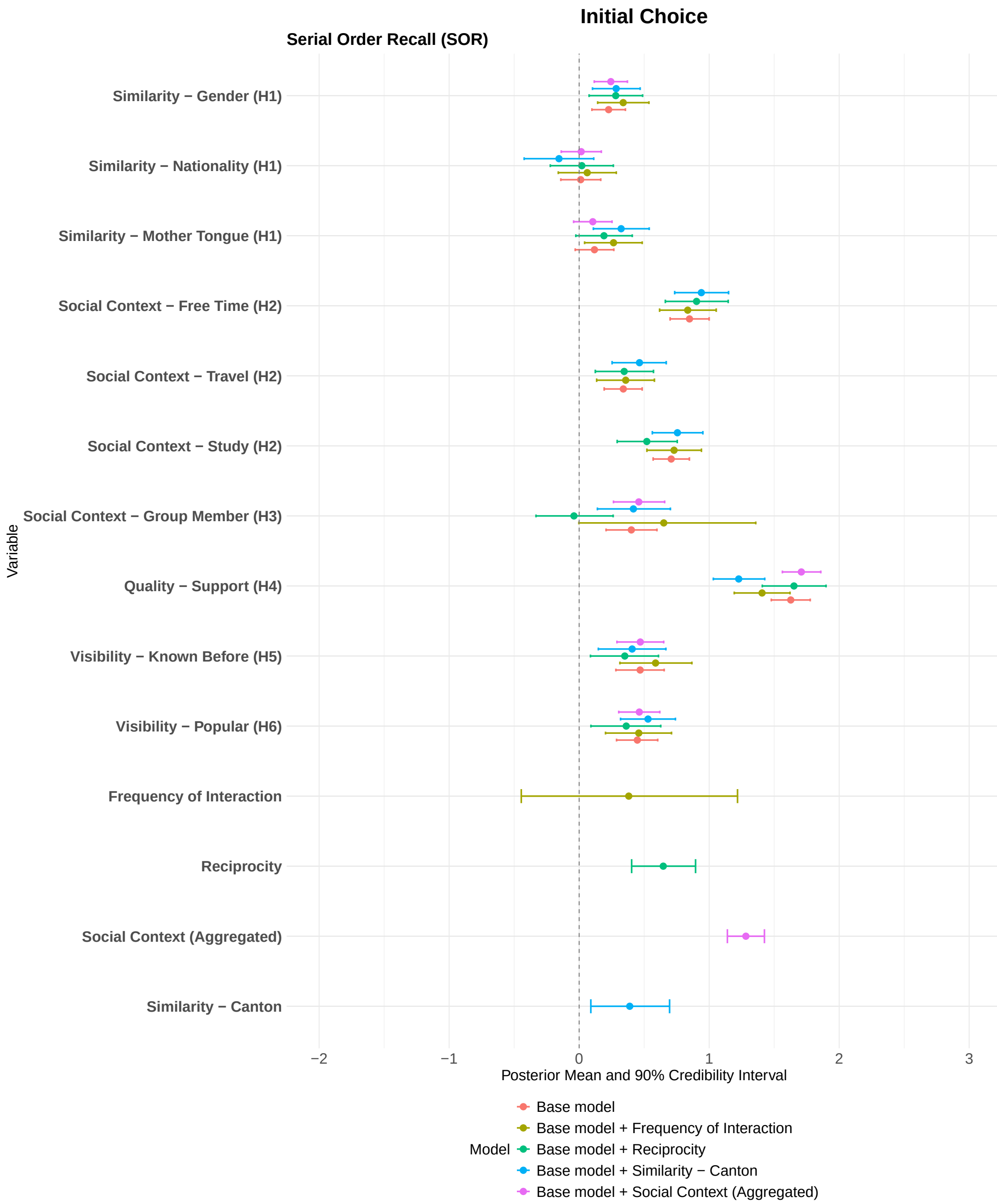

Figure 30. Comparing the base model with alternative models, using different Operationalisations and new predictors.

15 Subsequent Choices - Friendship

The following plots the results of each model for subsequent nominations of friendship sequences. The models are grouped (especially for the interaction models) so that similar configurations are together, and the effects represent the same abstract meaning.

15.1 Subsequent Choices - Friendship - Weak Prior

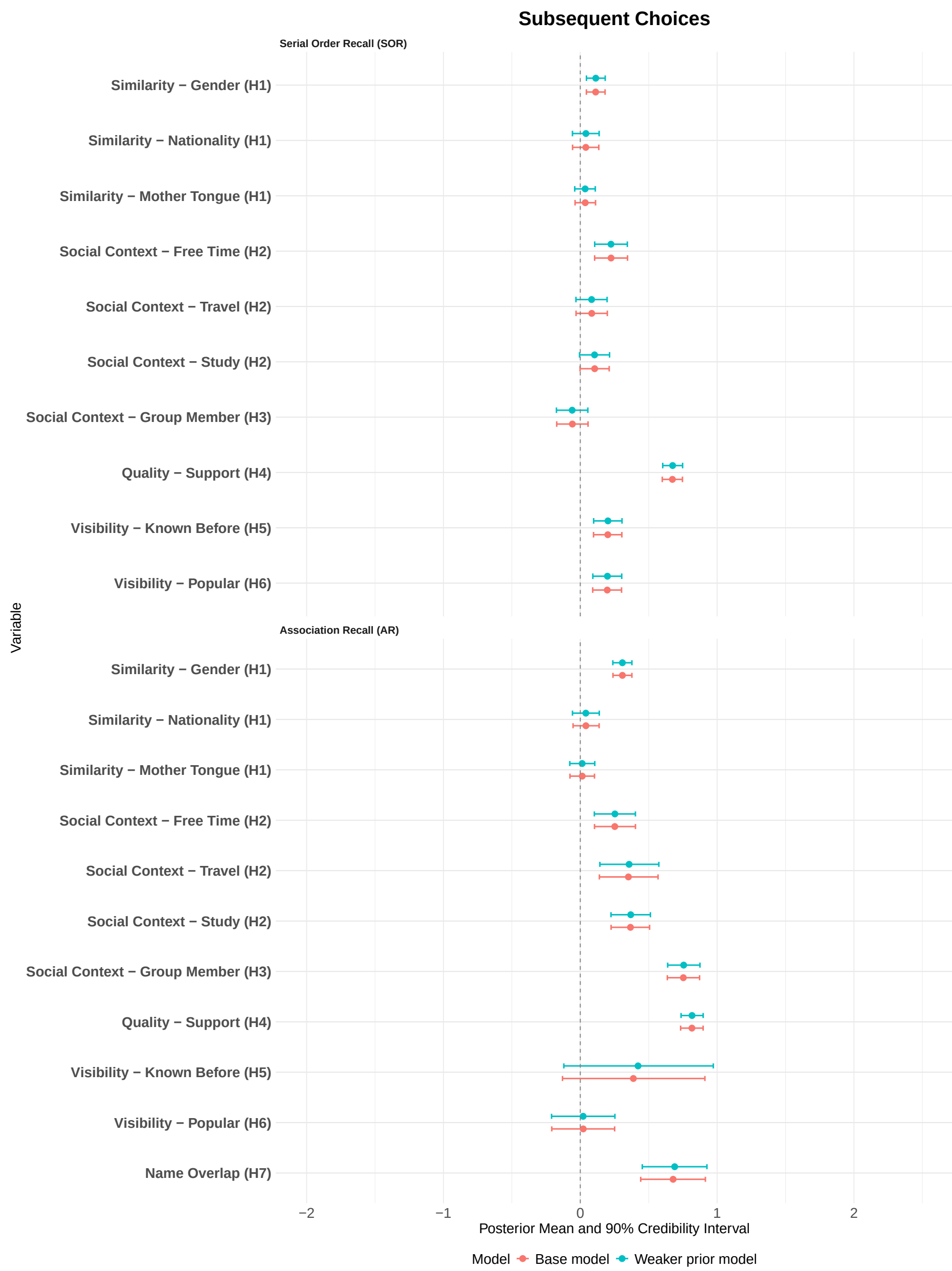

Figure 31. Comparing the base model with an alternative configuration with a weaker prior.

15.2 Subsequent Choices - Friendship - Regularized Horseshoe Prior (RHS)

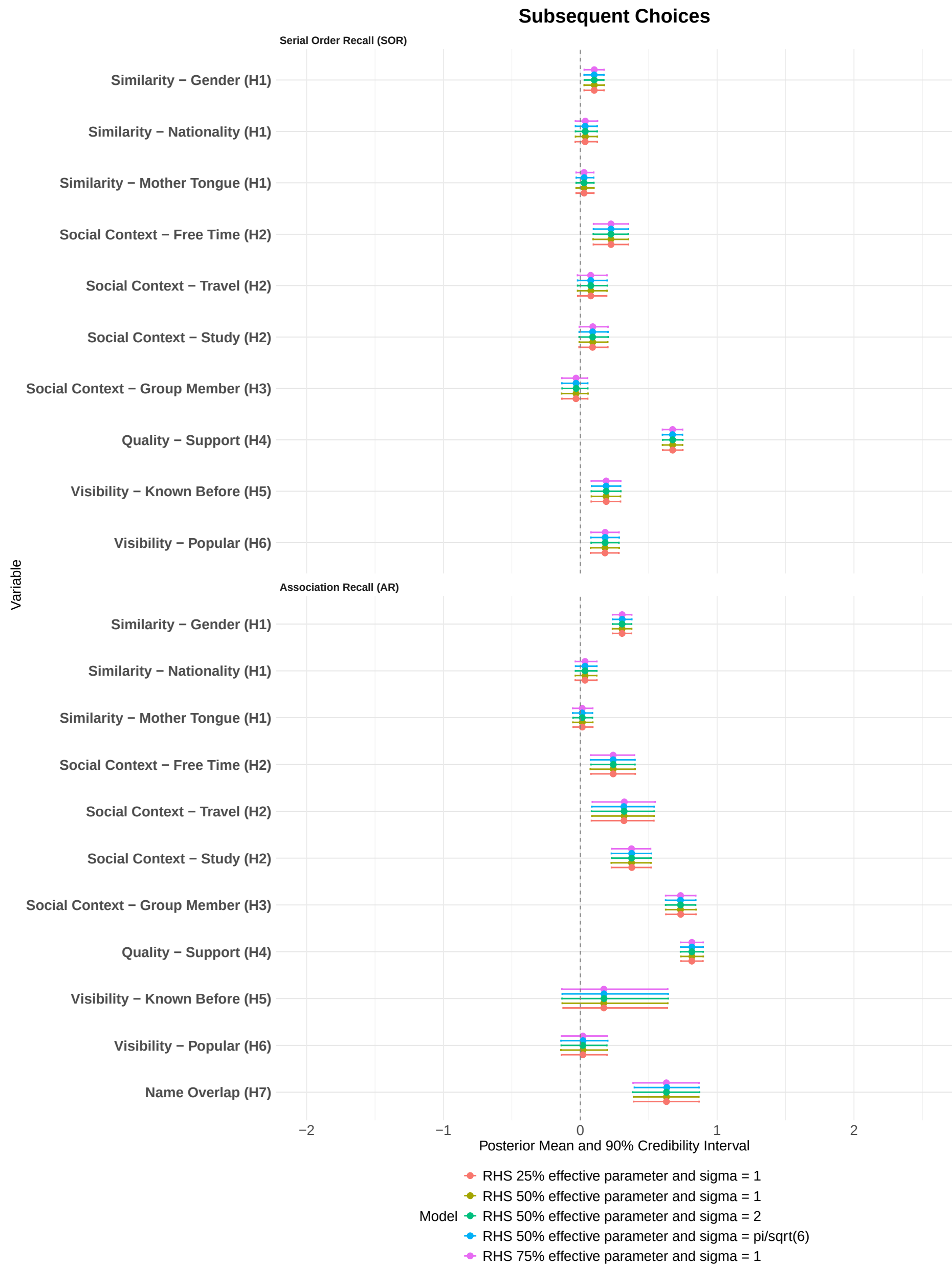

Figure 32. Comparing the base model with an alternative configuration with a regularized horseshoe prior under different configurations.

15.3 Subsequent Choices - Friendship - Time Interaction

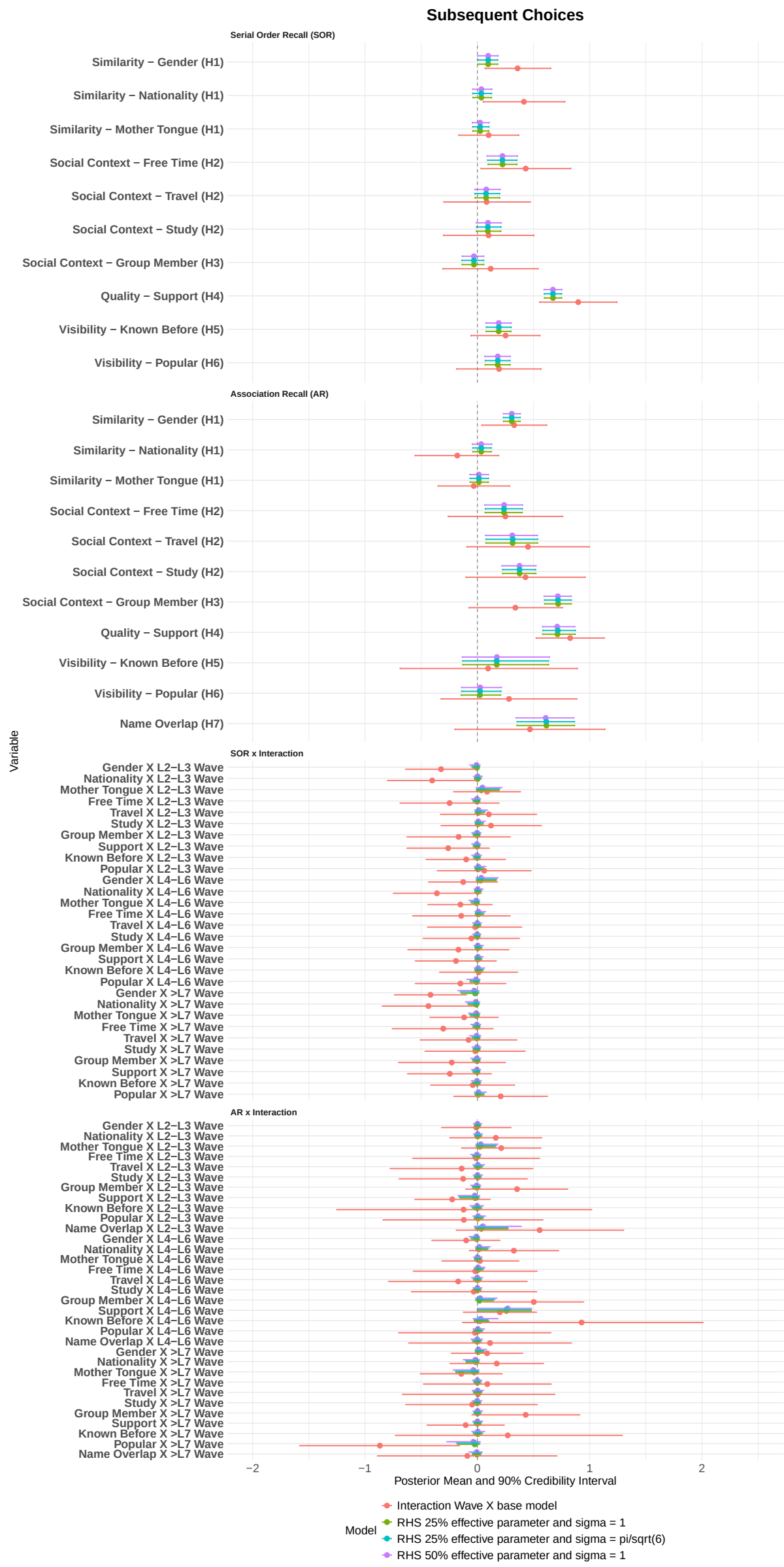

Figure 33. Comparing the base model with alternative models including time-related interactions.

15.4 Subsequent Choices - Friendship - Time Interaction with Focus on First Wave

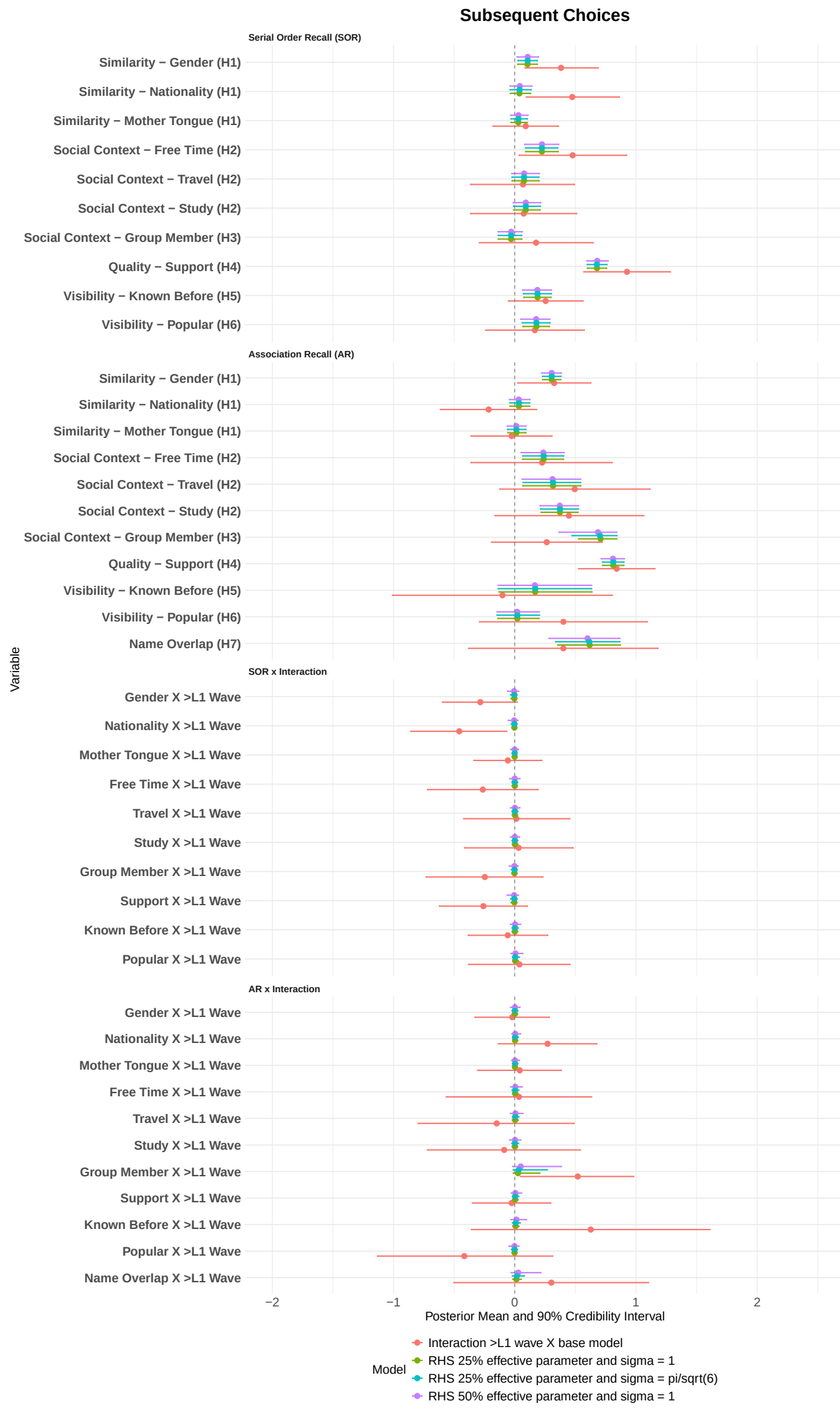

Figure 34. Comparing the base model with alternative models including time-related interactions - focusing only on Wave 1.

15.5 Subsequent Choices - Friendship - Sequence Length Interaction

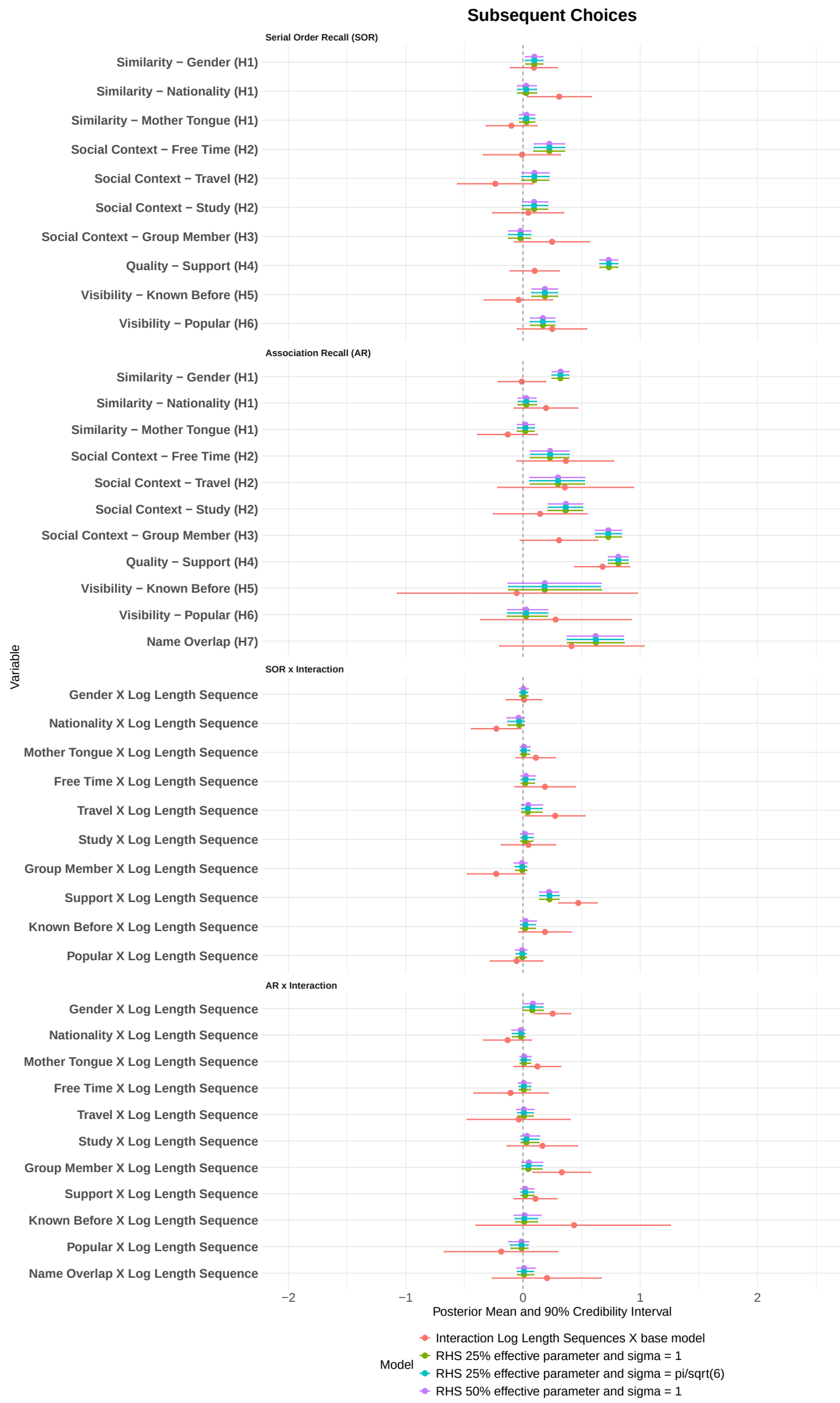

Figure 35. Comparing the base model with alternative models, including sequence length-related interactions.

15.6 Subsequent Choices - Friendship - Sequence Position Interactions

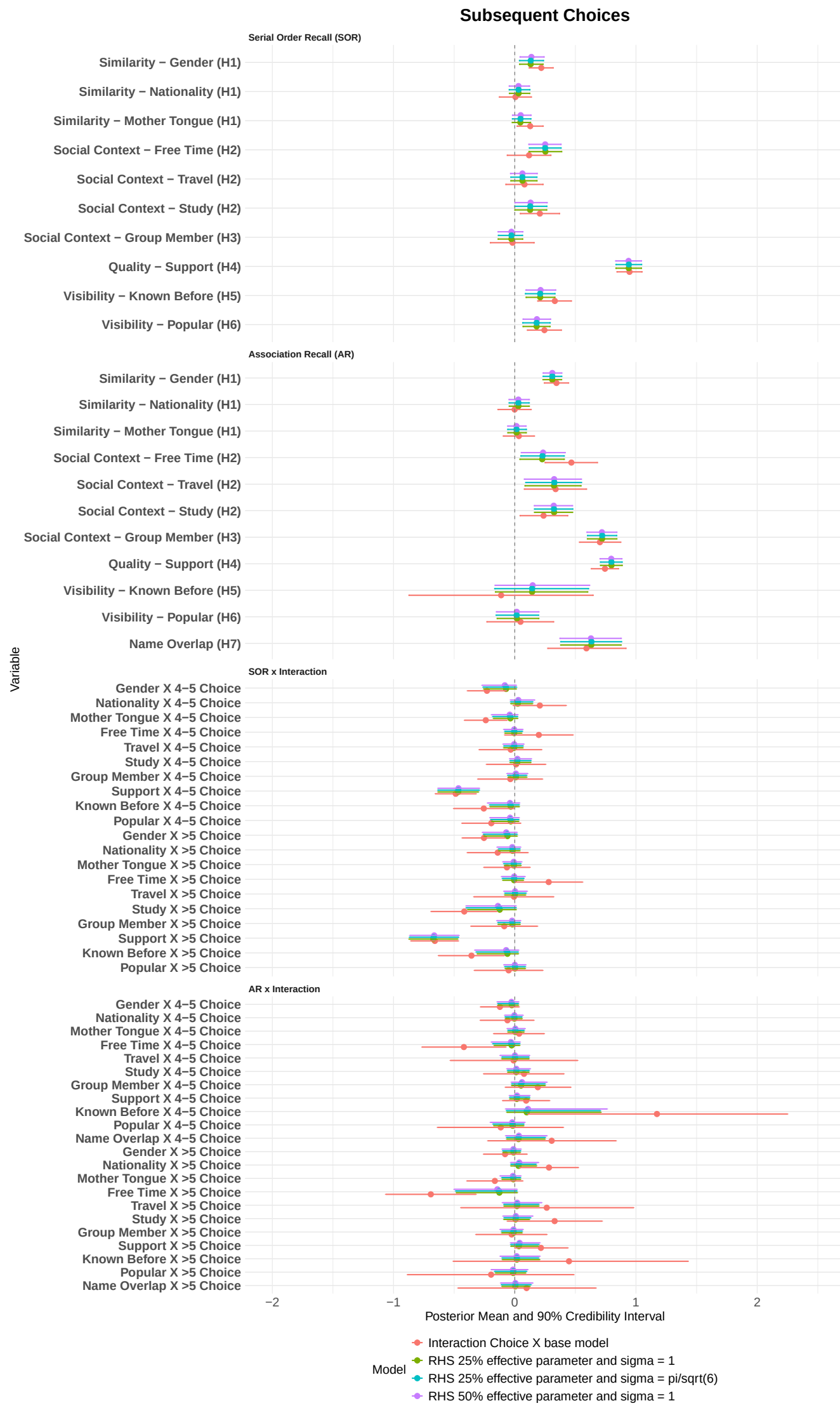

Figure 36. Comparing the base model with alternative models, including sequence position-related interactions.

15.7 Subsequent Choices - Friendship - Sequence Position Interactions with Focus on First 3 Nominations

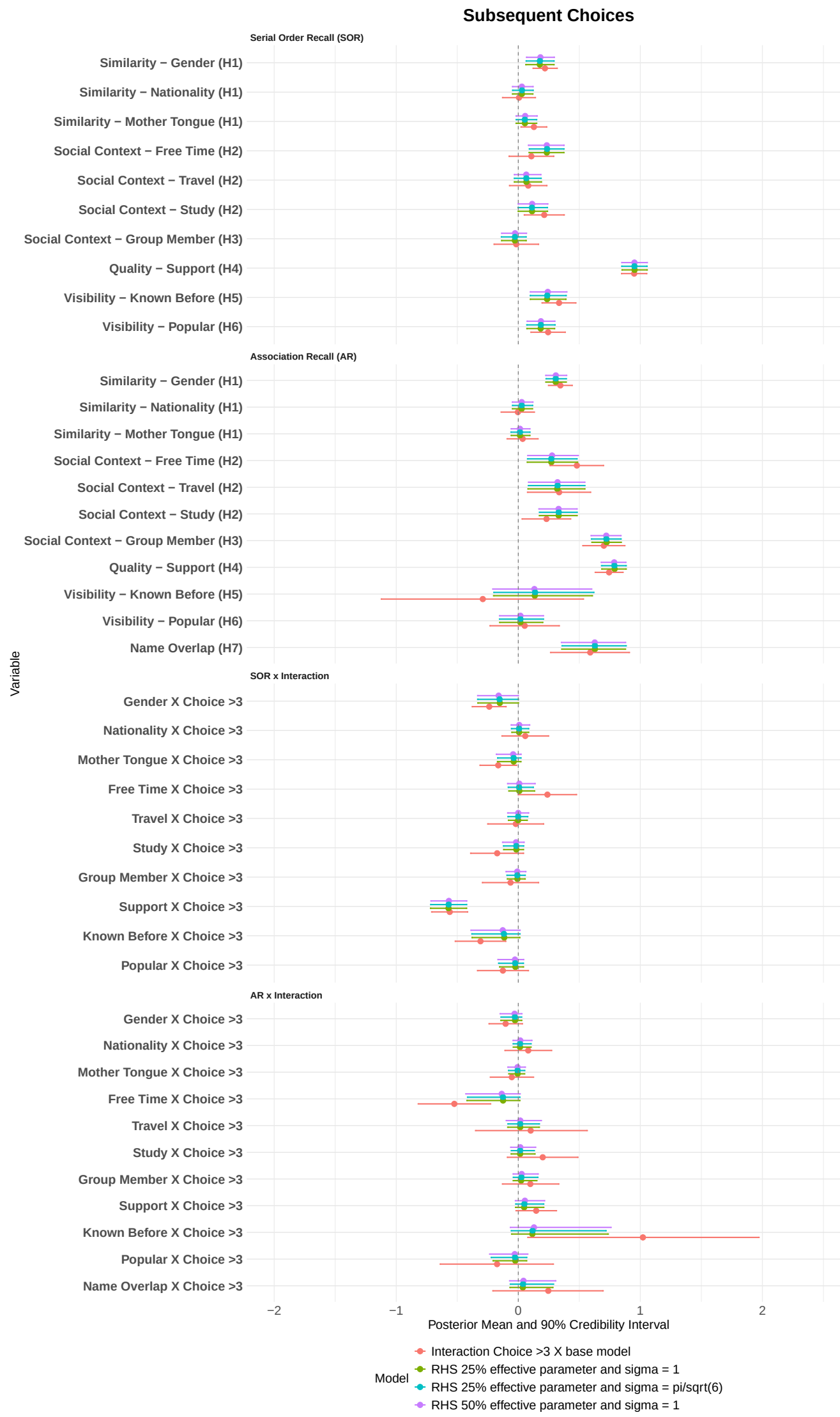

Figure 37. Comparing the base model with alternative models, including sequence position-related interactions, focusing on the first three nominations.

15.8 Subsequent Choices - Friendship - Sequence Position and Time Interactions

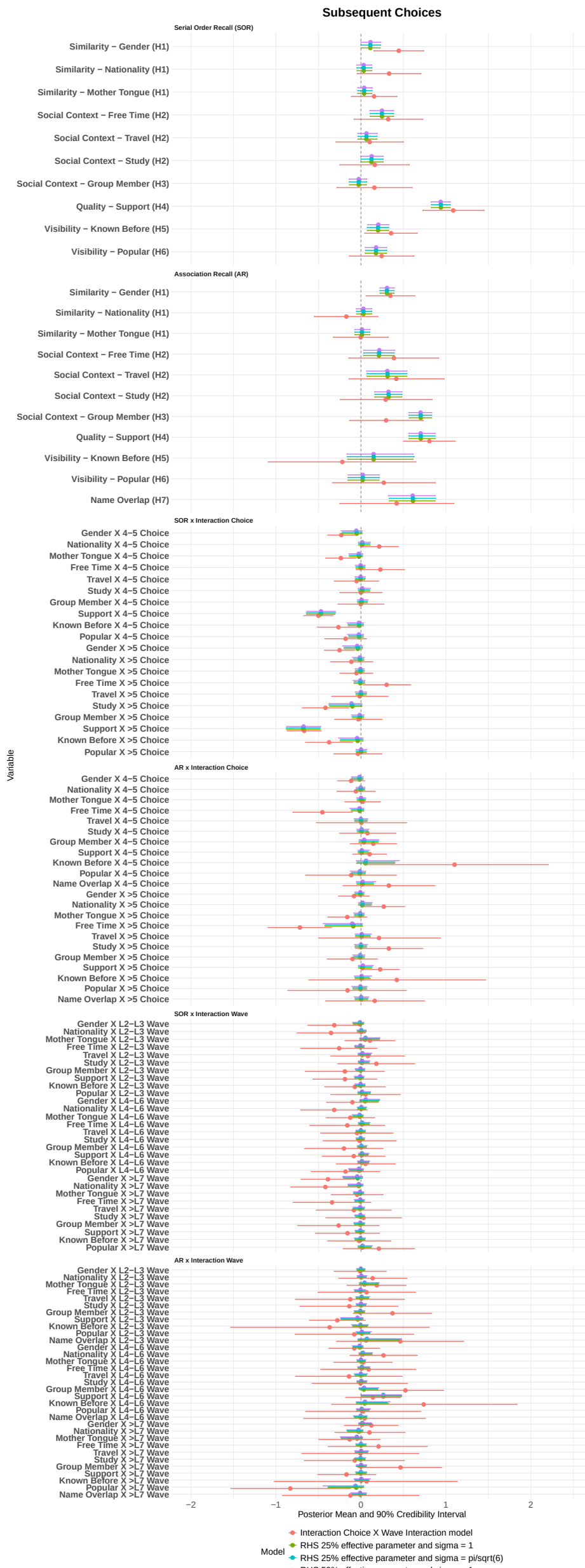

Figure 38. Comparing the base model with alternative models, including time-related and sequence position-related interactions.

15.9 Subsequent Choices - Friendship - Sequence Position Interactions with Focus on First 3 Nominations and Time Interactions with Focus on First Wave

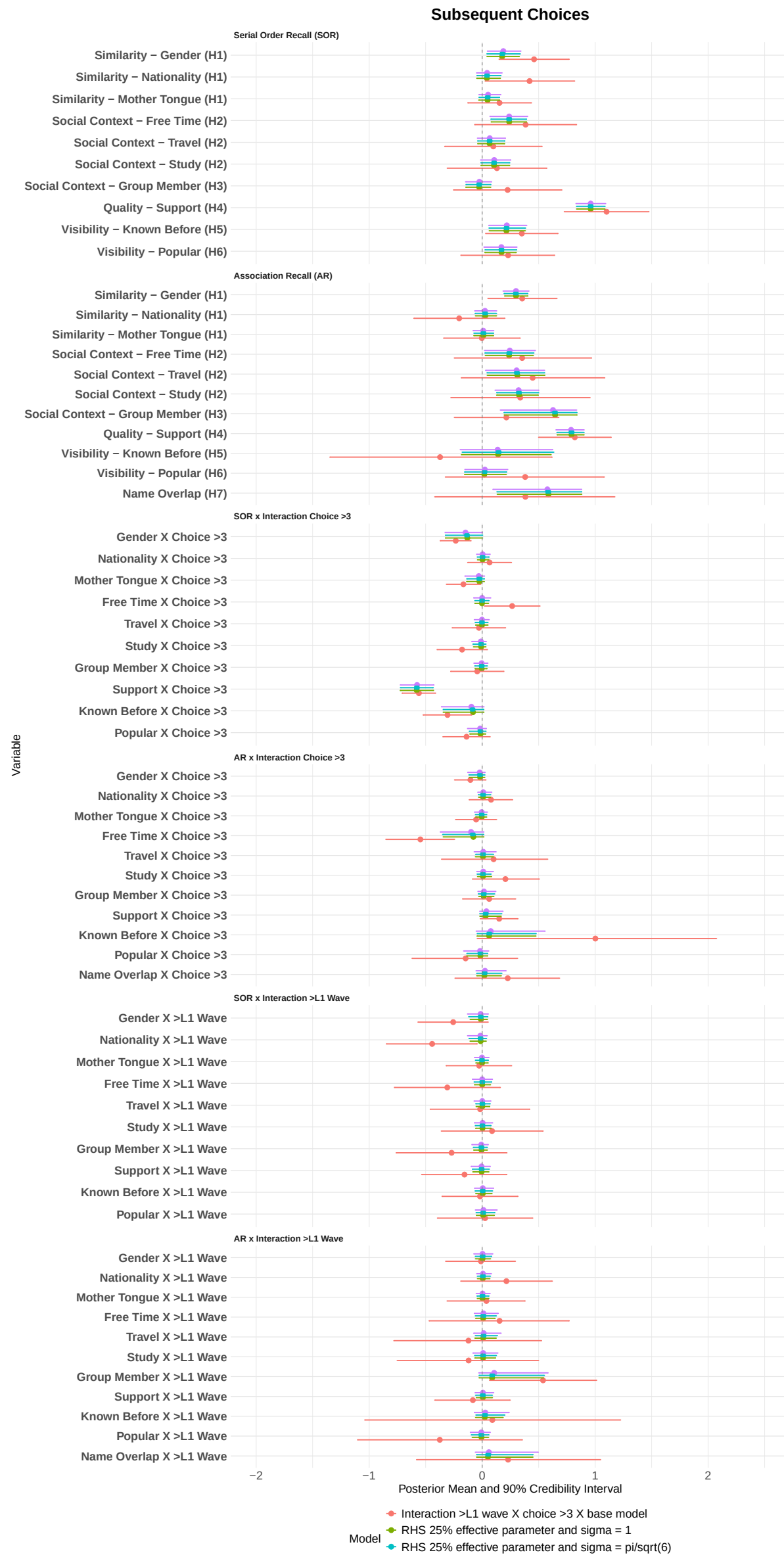

Figure 39. Comparing the base model with alternative models, including sequence position-related interactions, focusing on the first three nominations, and time-related interactions focusing on the first wave.

15.10 Subsequent Choices - Friendship - Random Slope for Individuals

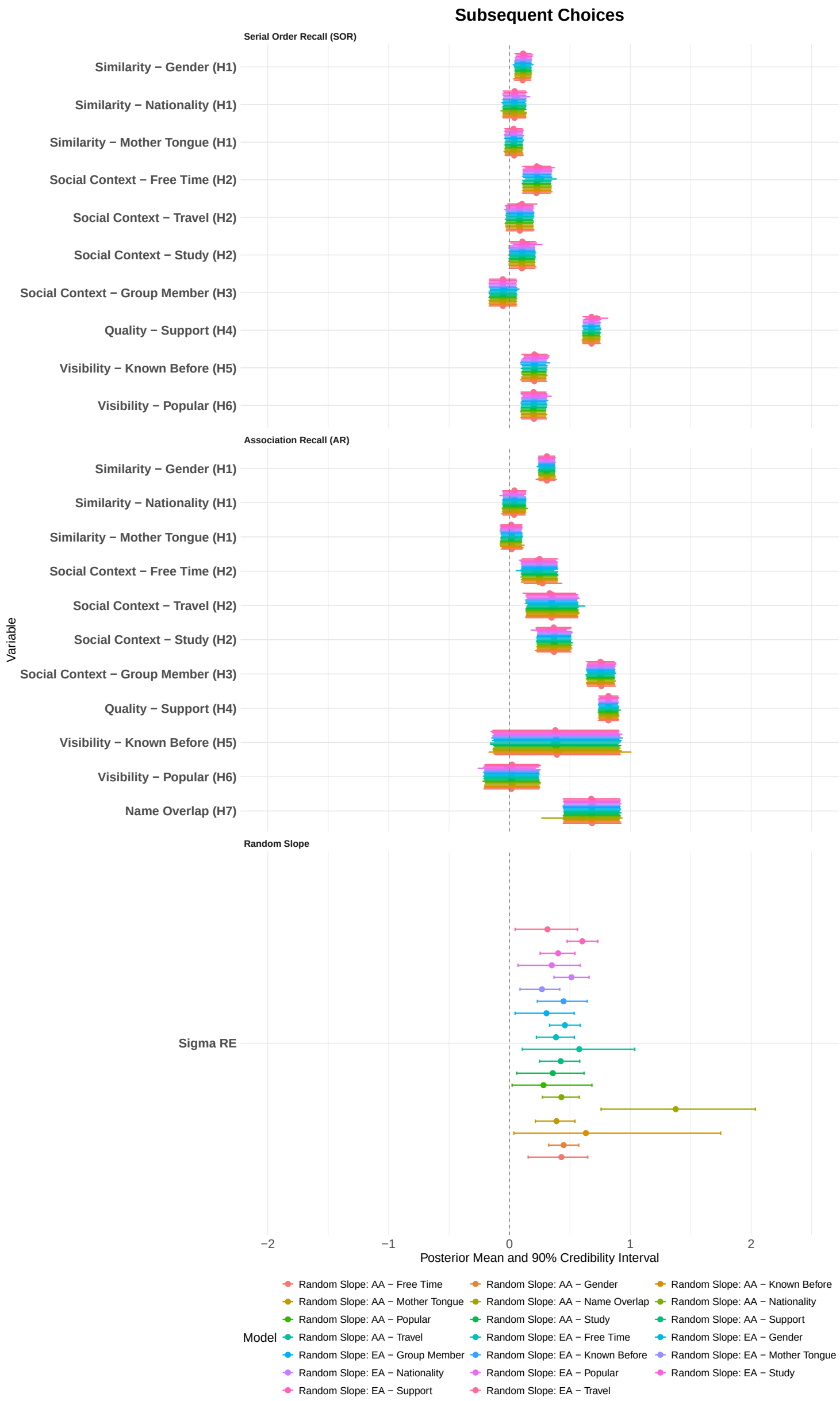

Figure 40. Comparing the base model with alternative models, including a random slope for each predictor separately based on individuals.

15.11 Subsequent Choices - Friendship - MICE imputation

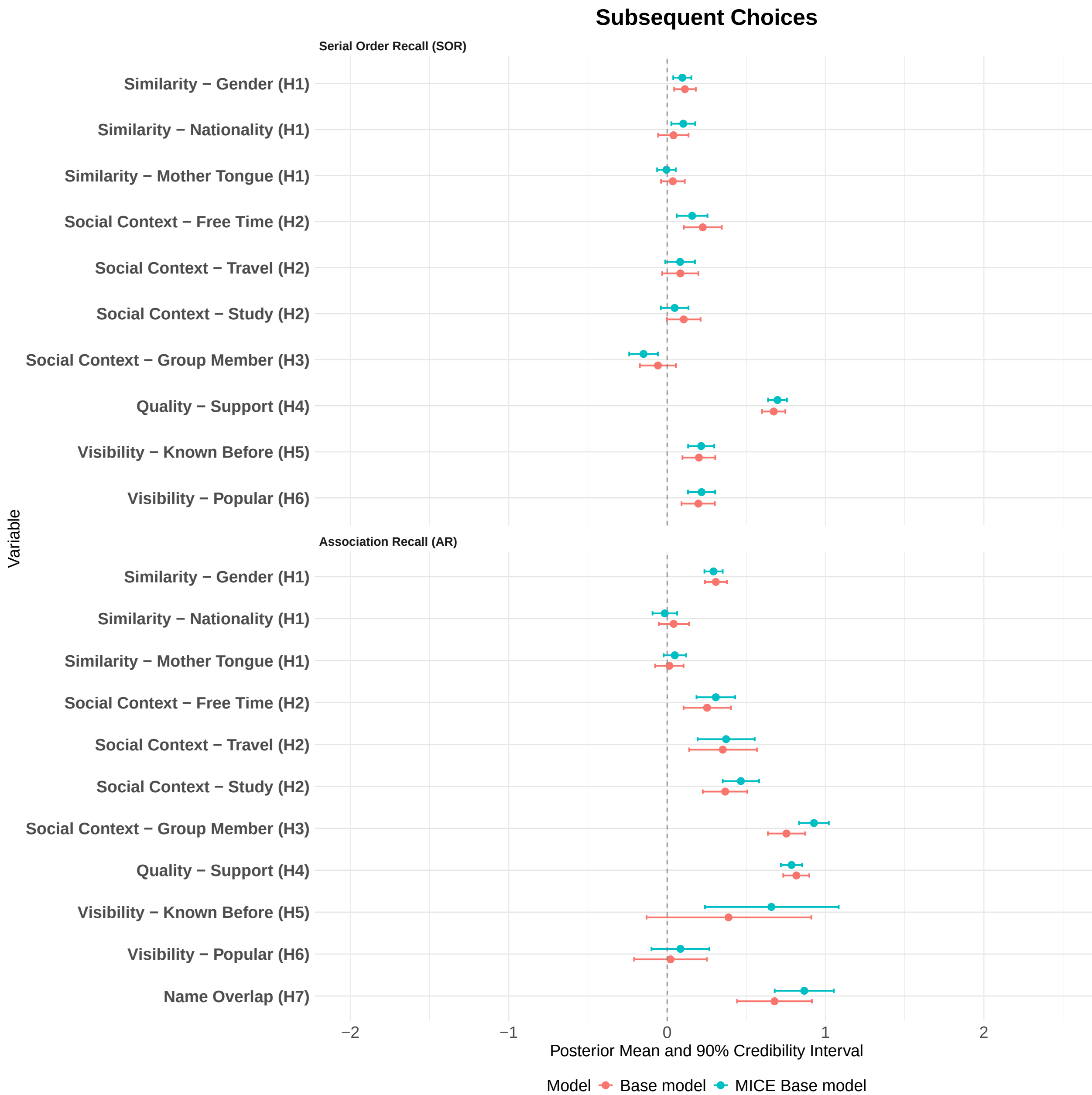

Figure 41. Comparing the base model with an alternative model using the mice imputed datasets.

15.12 Subsequent Choices - Friendship - Reduced Choice set

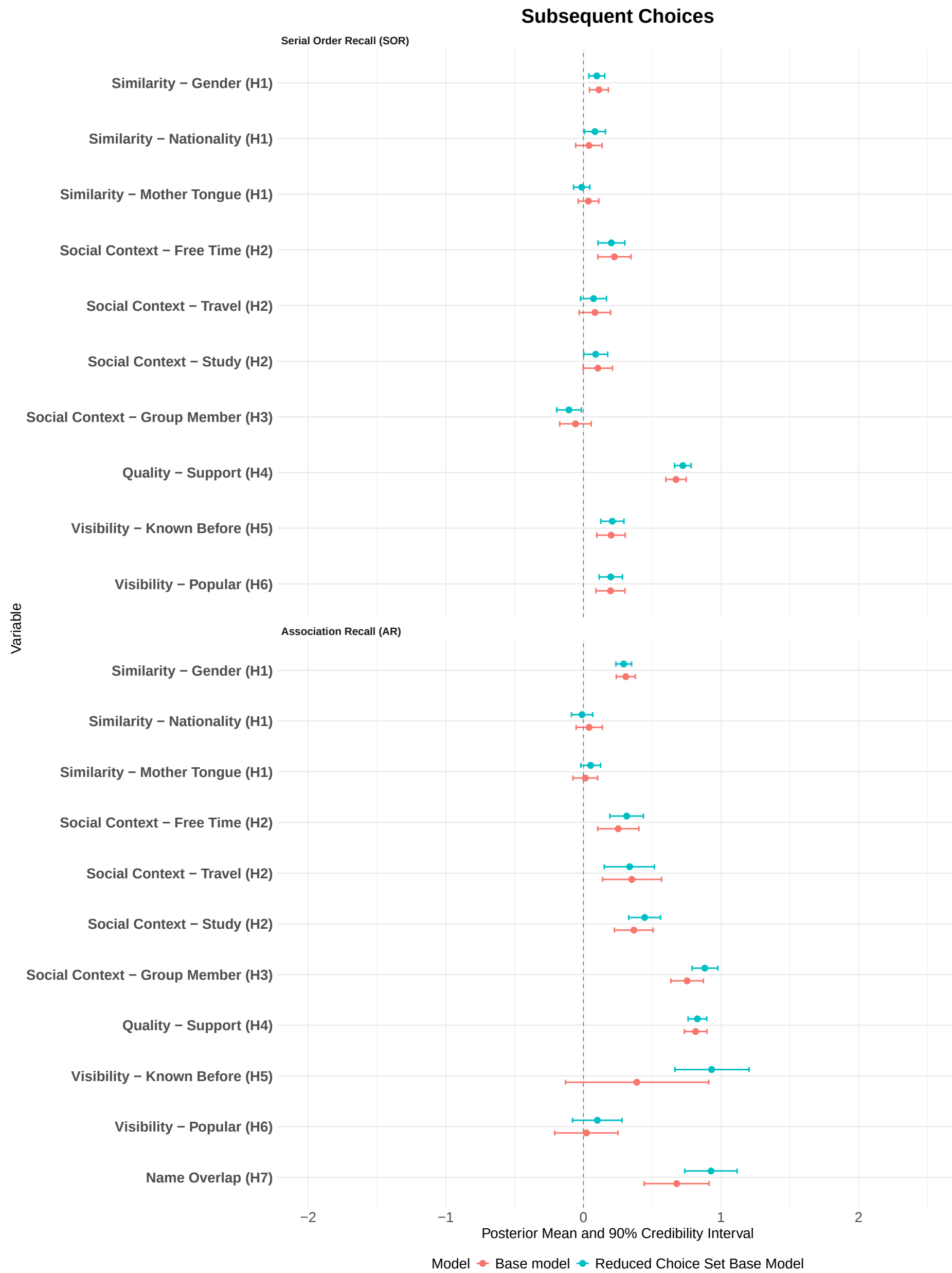

Figure 42. Comparing the base model with an alternative model using the reduced choice sets.

15.13 Subsequent Choices - Friendship - Explorative Robustness Operationalisations

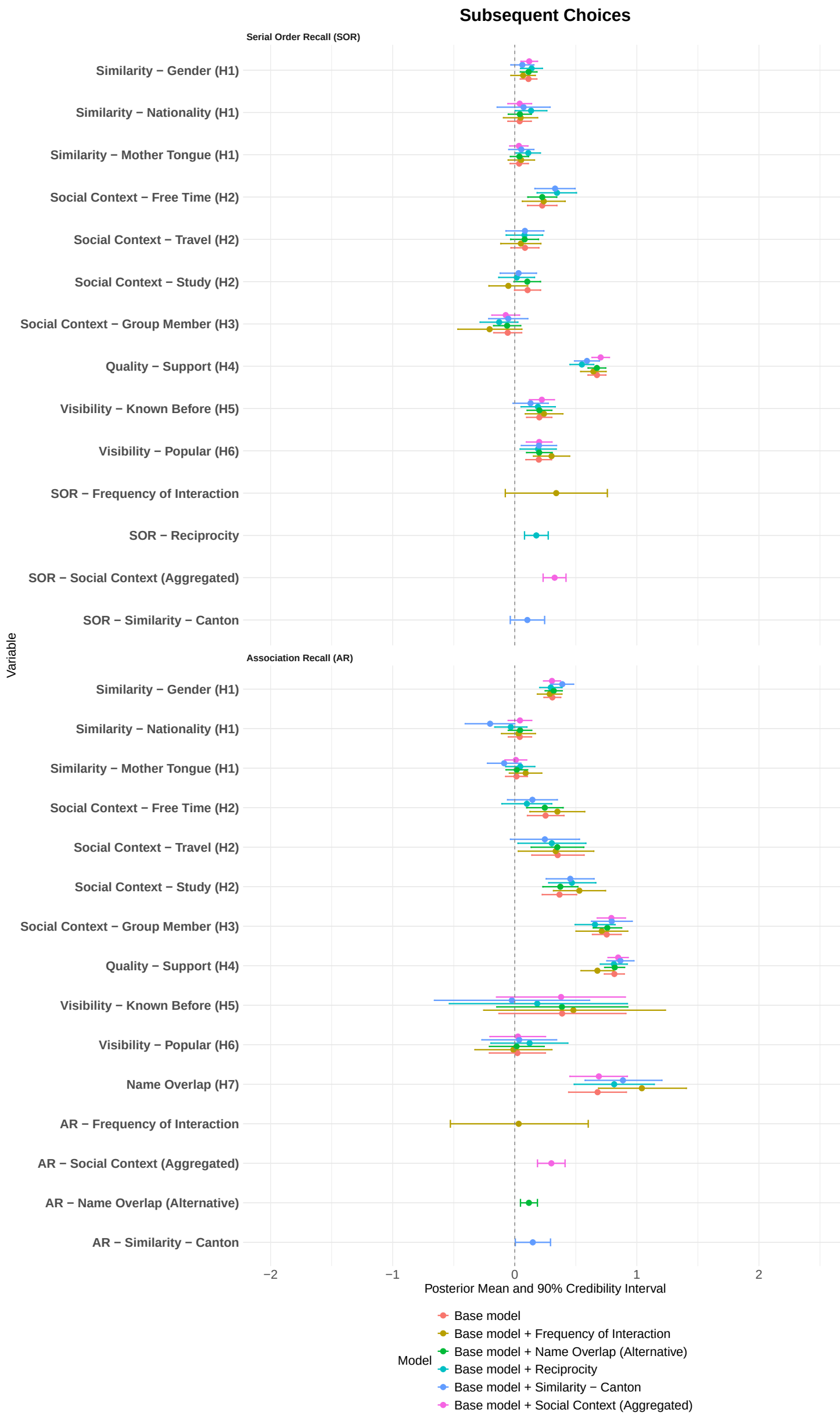

Figure 43. Comparing the base model with alternative models, using different Operationalisations and new predictors.

16 Subsequent Choices - Positive Interaction

The following plots the results of each model for subsequent nominations of positive interaction sequences. The models are grouped (especially for the interaction models) so that similar configurations are together, and the effects represent the same abstract meaning.

16.1 Subsequent Choices - Positive Interaction - Weak Prior

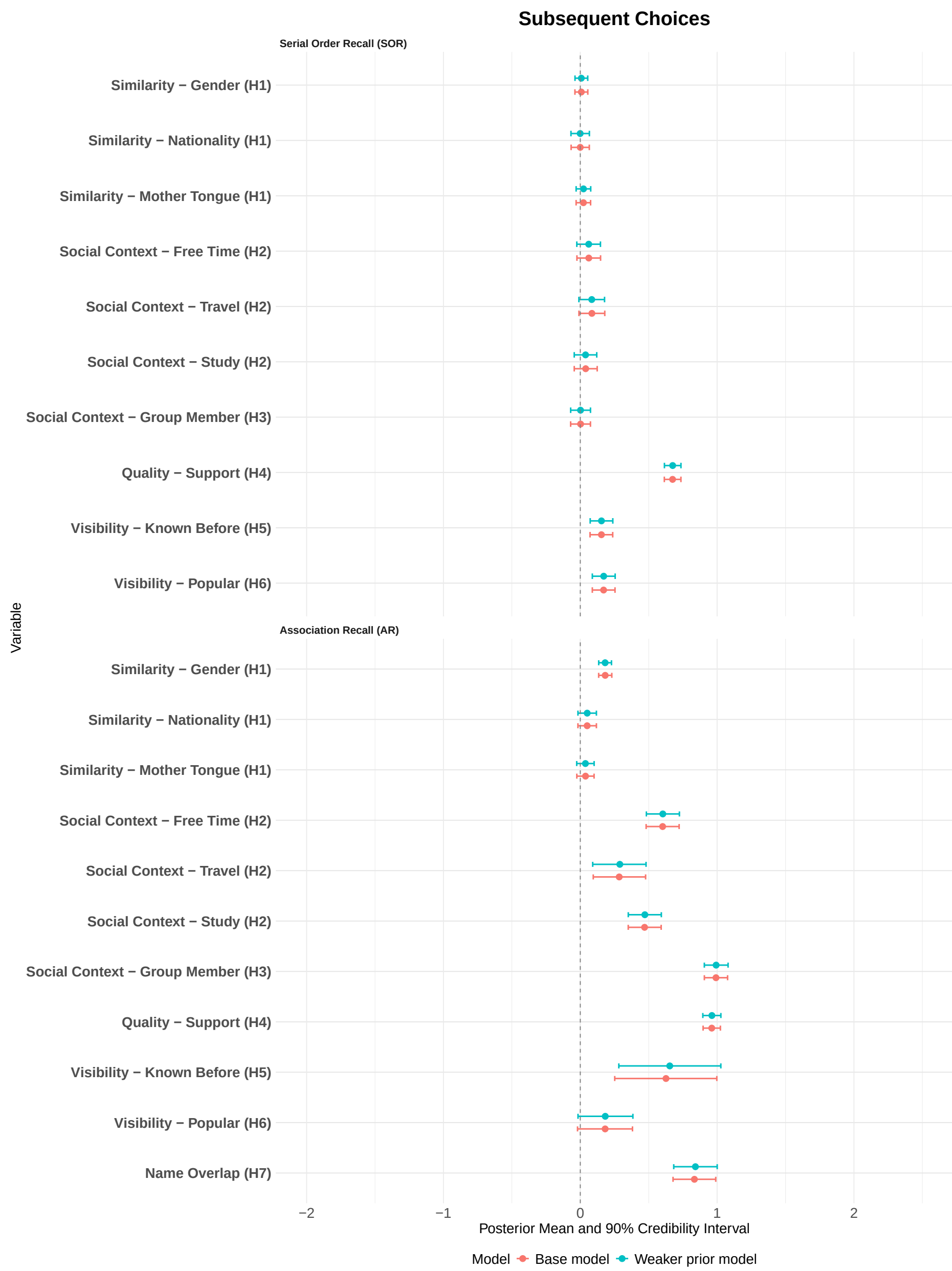

Figure 44. Comparing the base model with an alternative configuration with a weaker prior.

16.2 Subsequent Choices - Positive Interaction - Regularized Horseshoe Prior (RHS)

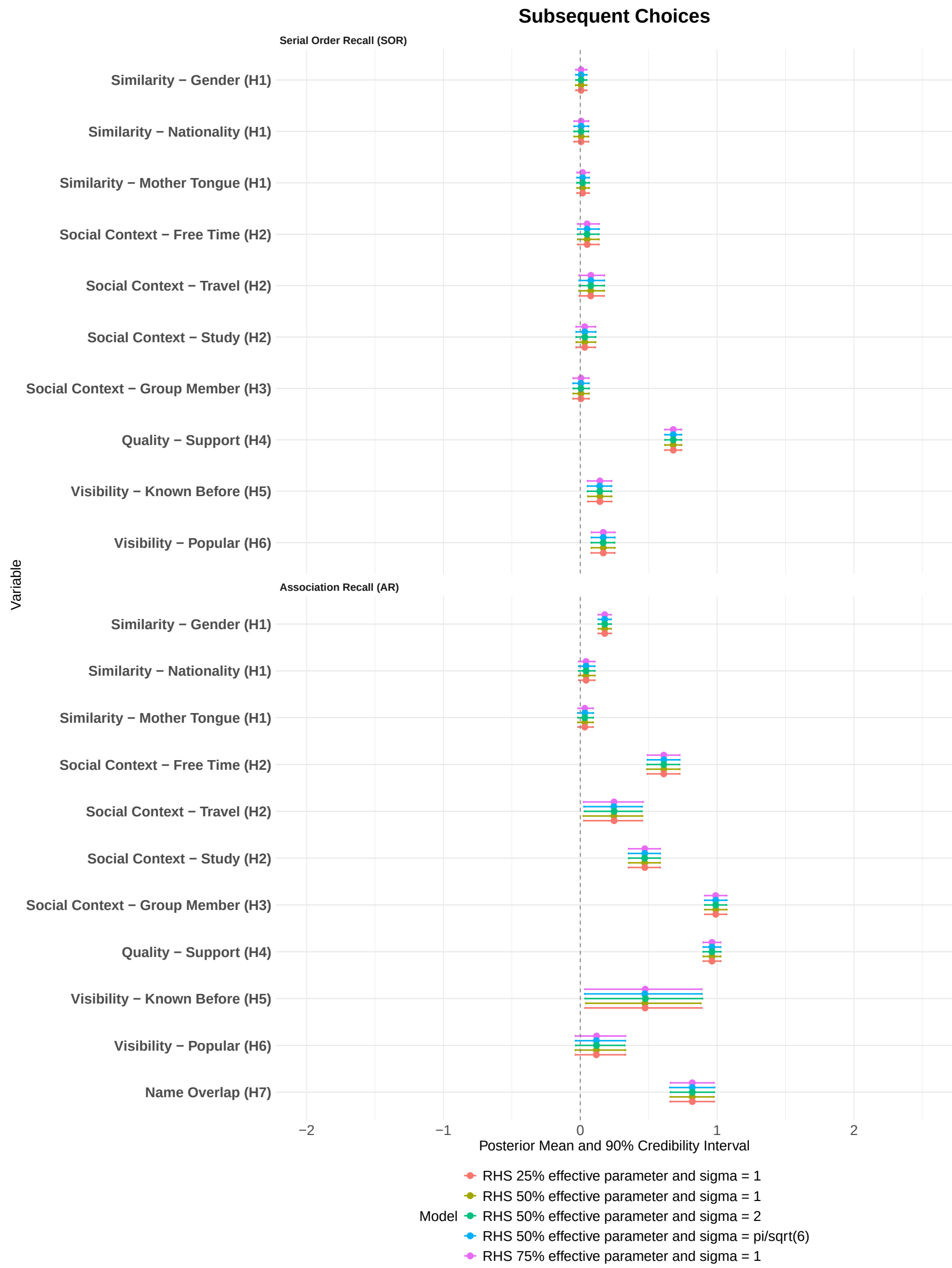

Figure 45. Comparing the base model with an alternative configuration with a regularized horseshoe prior under different configurations.

16.3 Subsequent Choices - Positive Interaction - Time Interaction

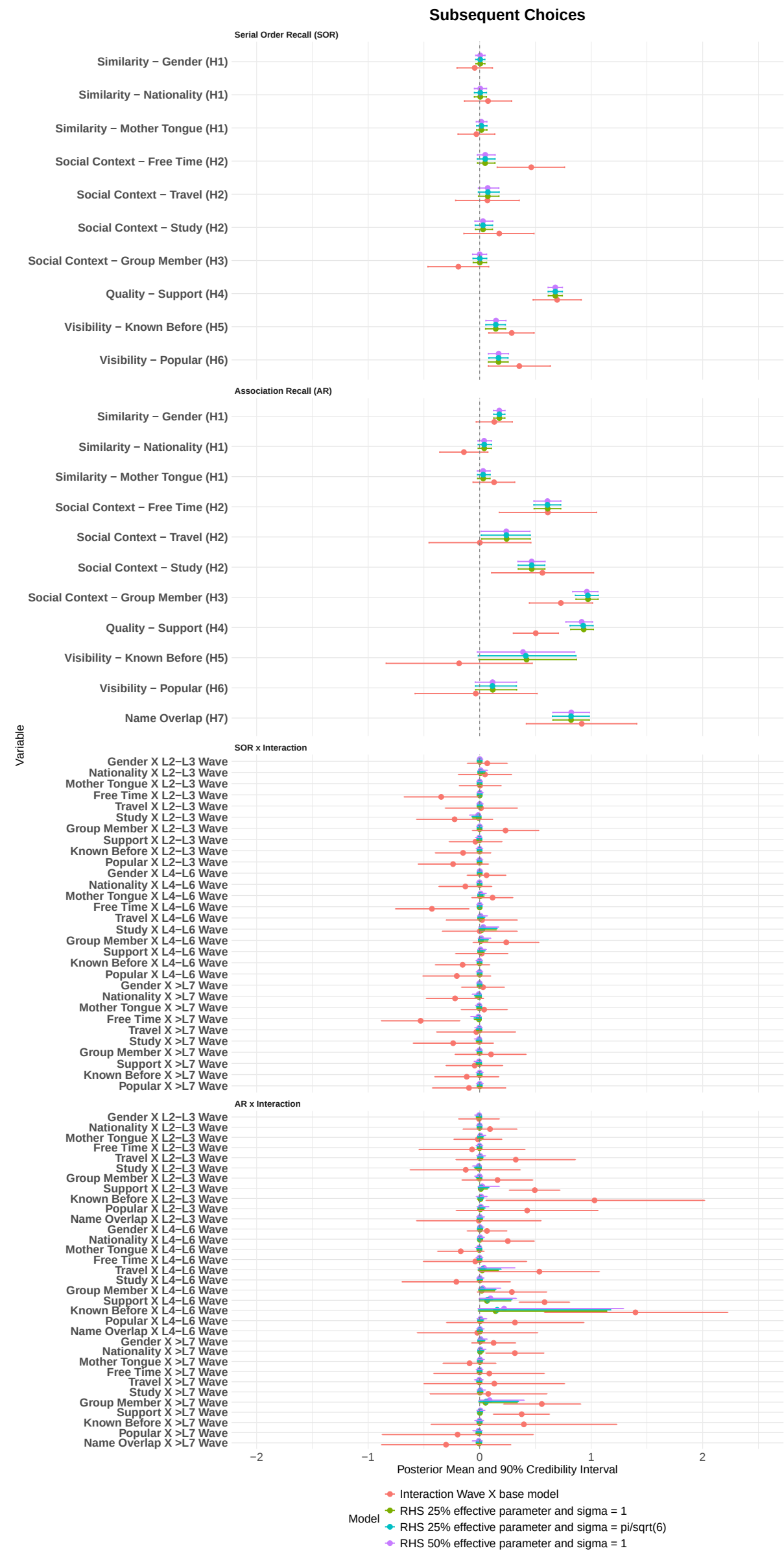

Figure 46. Comparing the base model with alternative models including time-related interactions.

16.4 Subsequent Choices - Positive Interaction - Time Interaction with Focus on First Wave

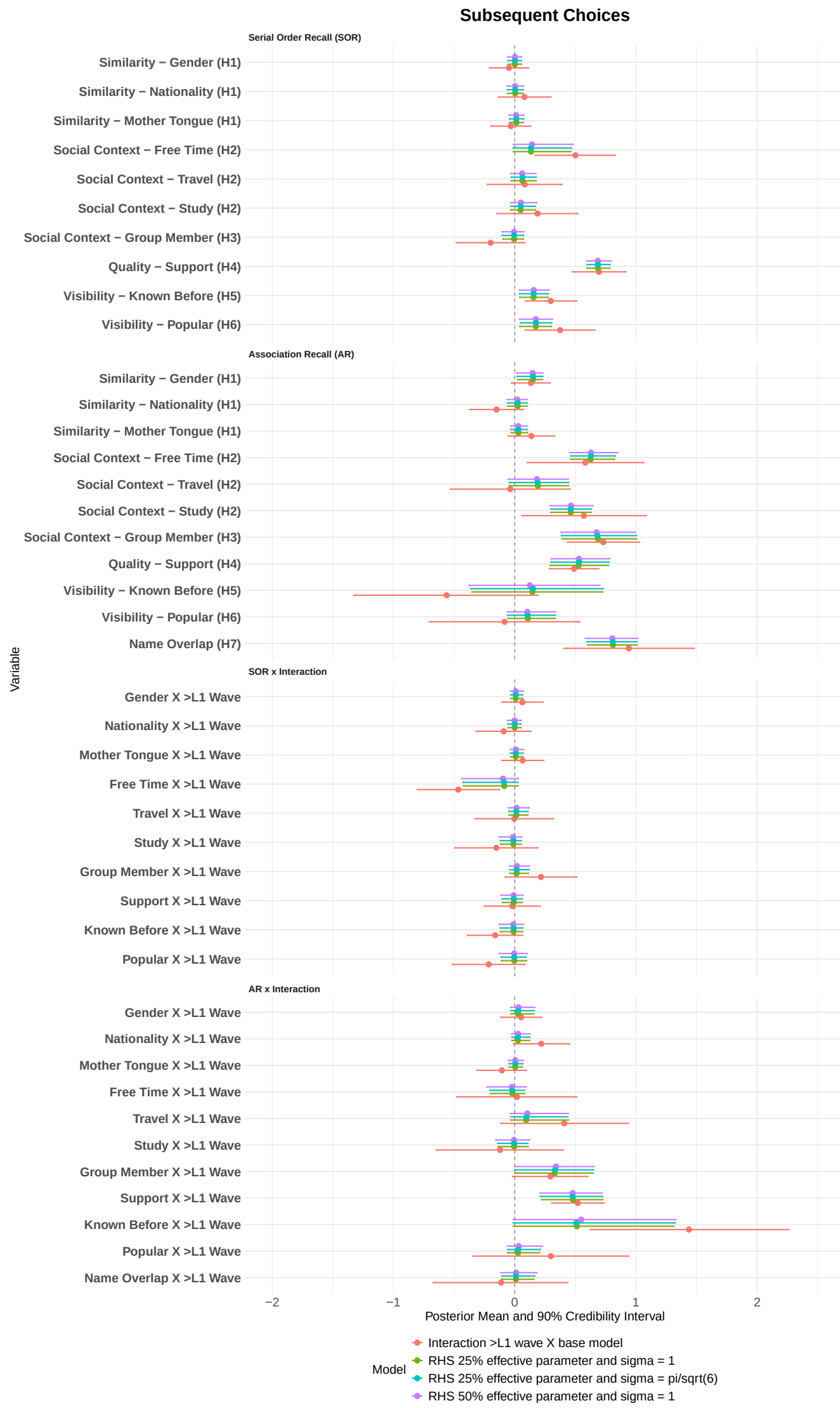

Figure 47. Comparing the base model with alternative models including time-related interactions - focusing only on Wave 1.

16.5 Subsequent Choices - Positive Interaction - Sequence Length Interaction

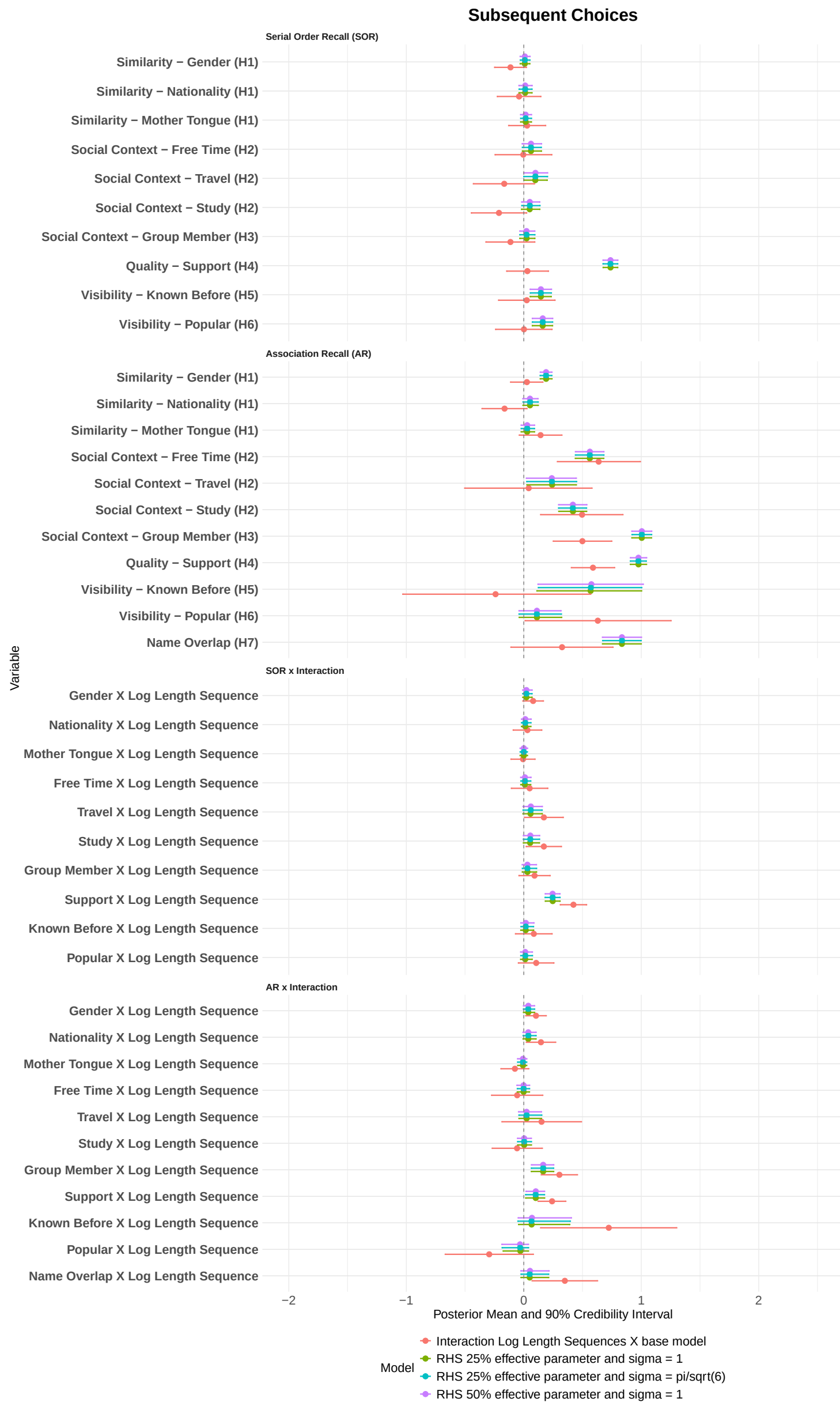

Figure 48. Comparing the base model with alternative models, including sequence length-related interactions.

16.6 Subsequent Choices - Positive Interaction - Sequence Position Interactions

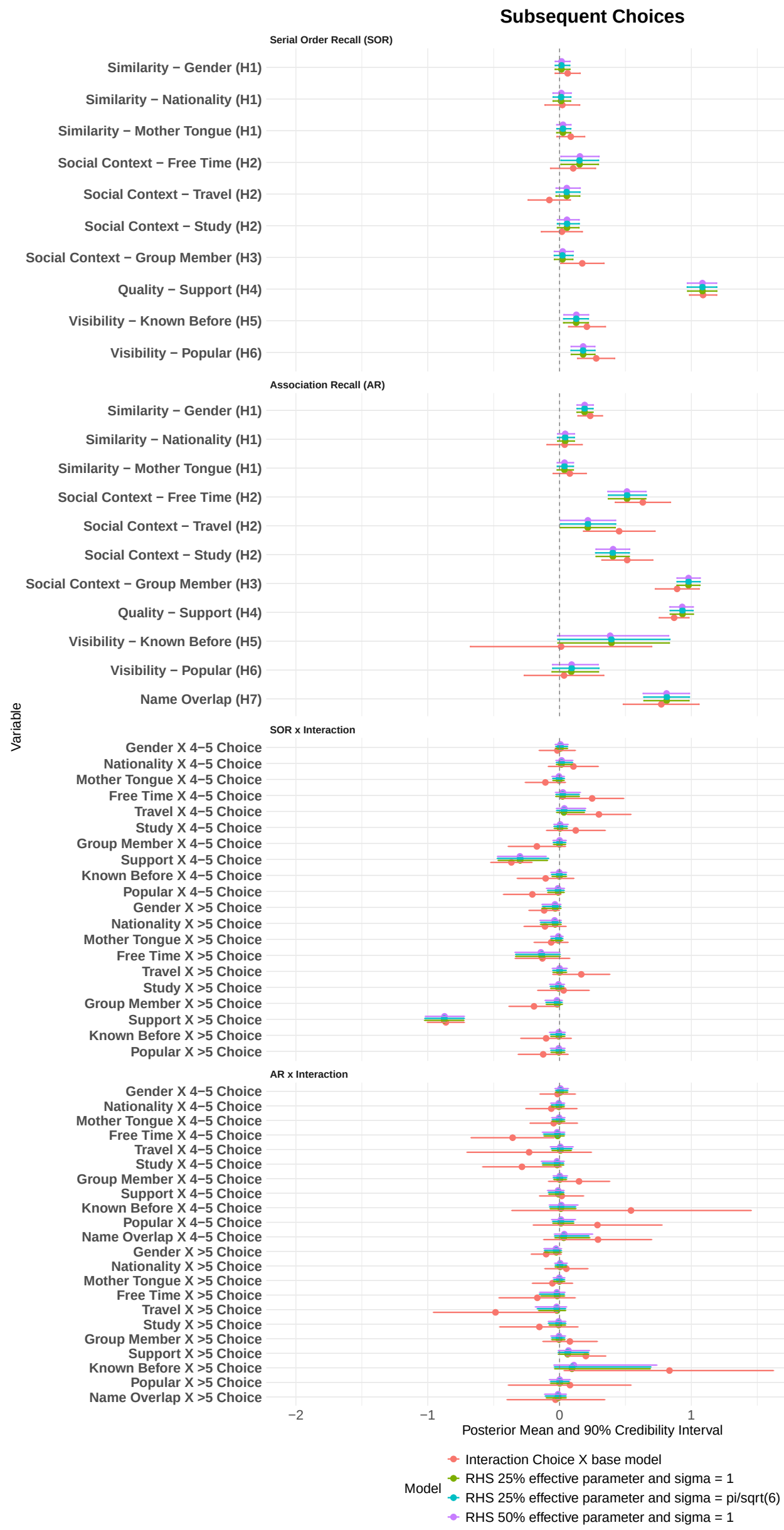

Figure 49. Comparing the base model with alternative models, including sequence position-related interactions.

16.7 Subsequent Choices - Positive Interaction - Sequence Position Interactions with Focus on First 3 Nominations

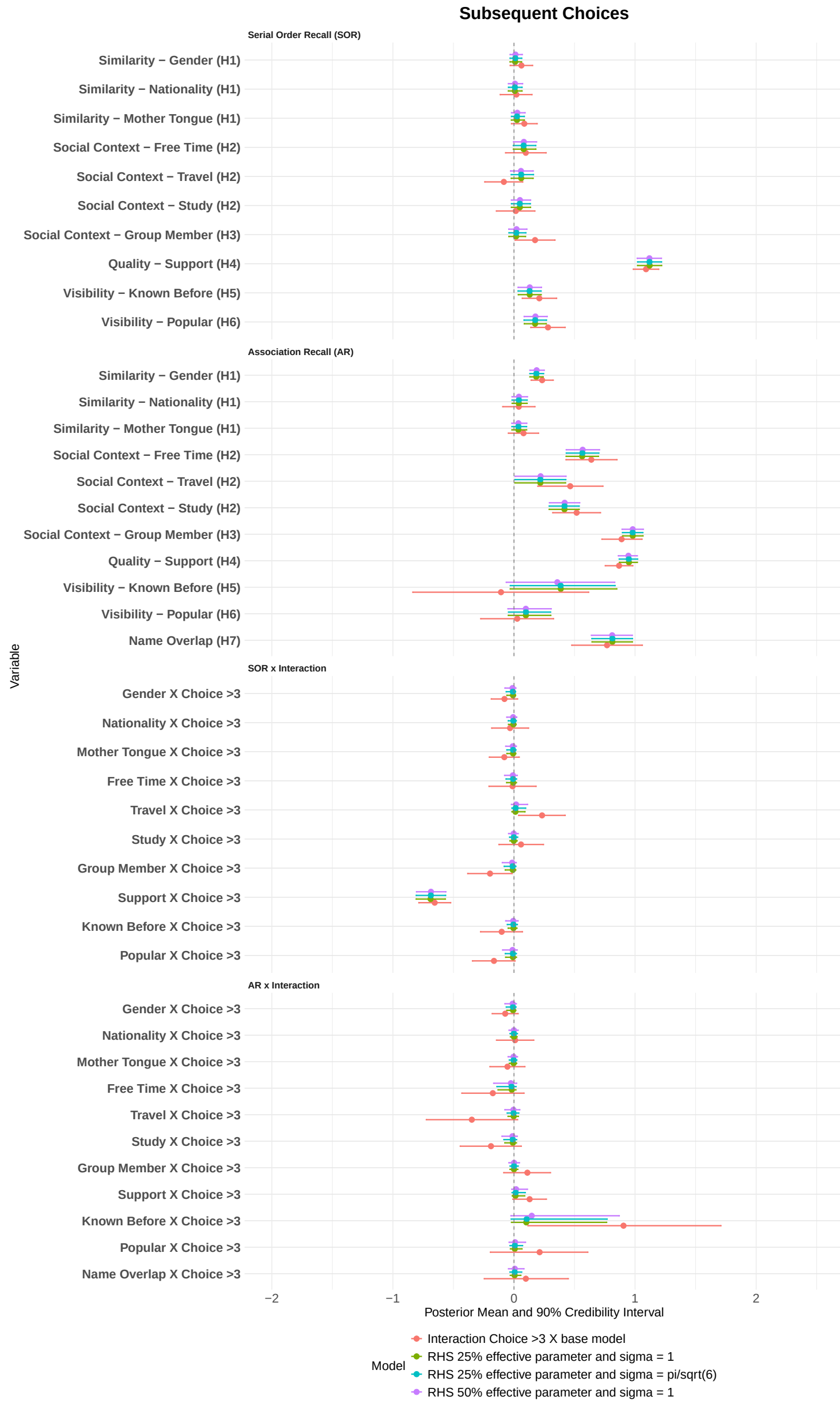

Figure 50. Comparing the base model with alternative models, including sequence position-related interactions, focusing on the first three nominations.

16.8 Subsequent Choices - Positive Interaction - Sequence Position and Time Interactions

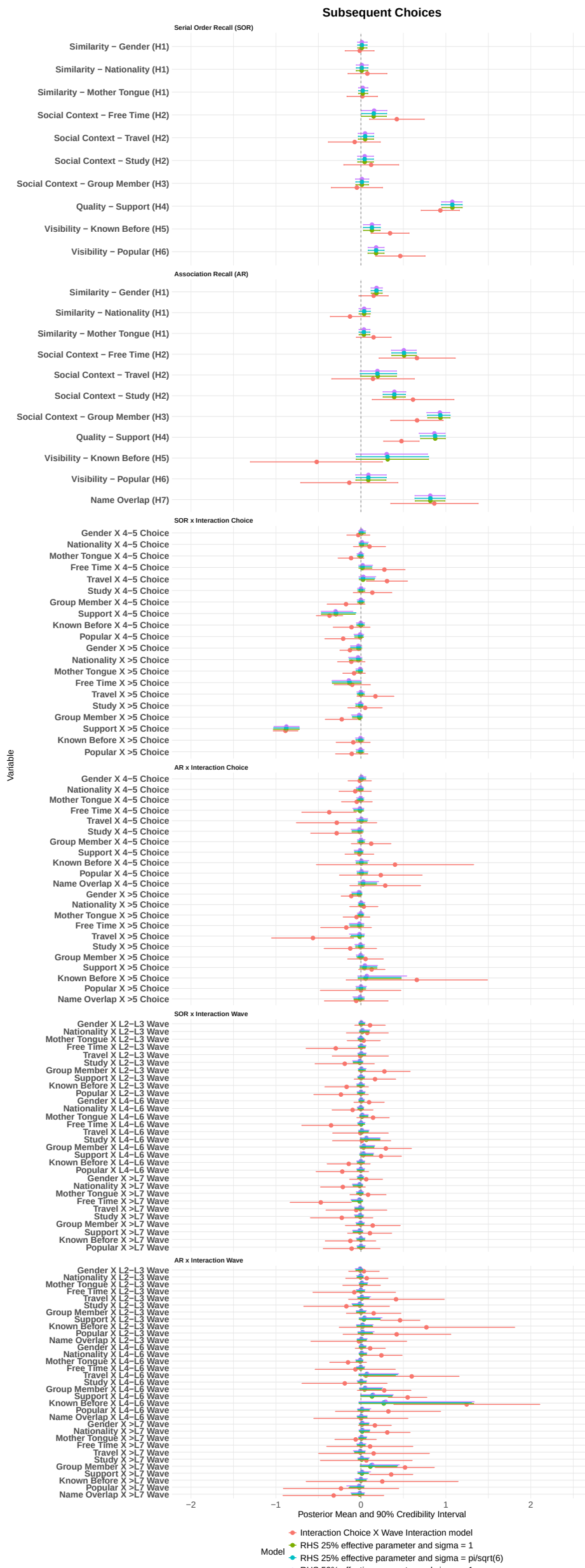

Figure 51. Comparing the base model with alternative models, including time-related and sequence position-related interactions.

16.9 Subsequent Choices - Positive Interaction - Sequence Position Interactions with Focus on First 3 Nominations and Time Interactions with Focus on First Wave

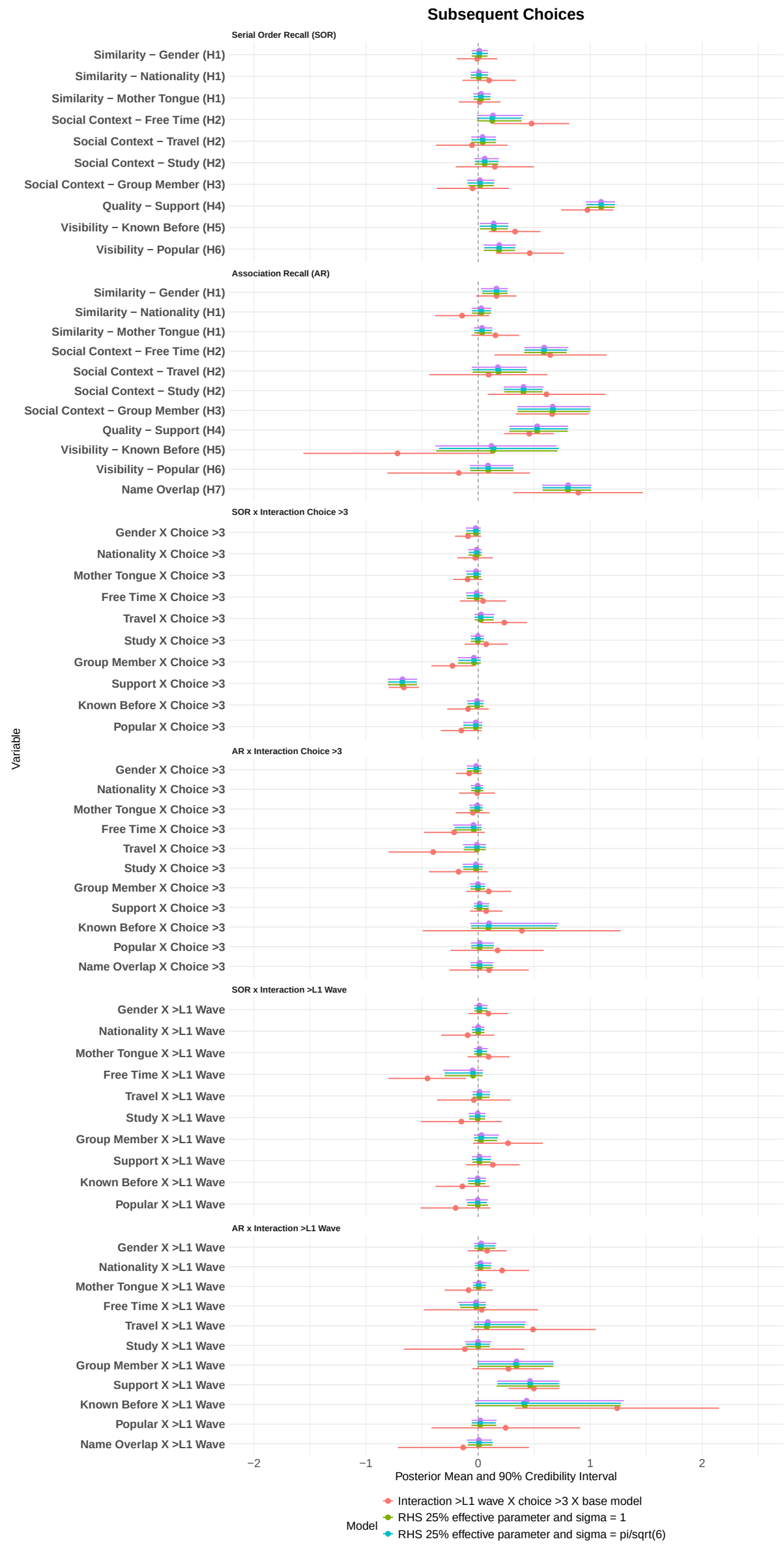

Figure 52. Comparing the base model with alternative models, including sequence position-related interactions, focusing on the first three nominations, and time-related interactions focusing on the first wave.

16.10 Subsequent Choices - Positive Interaction - Random Slope for Individuals

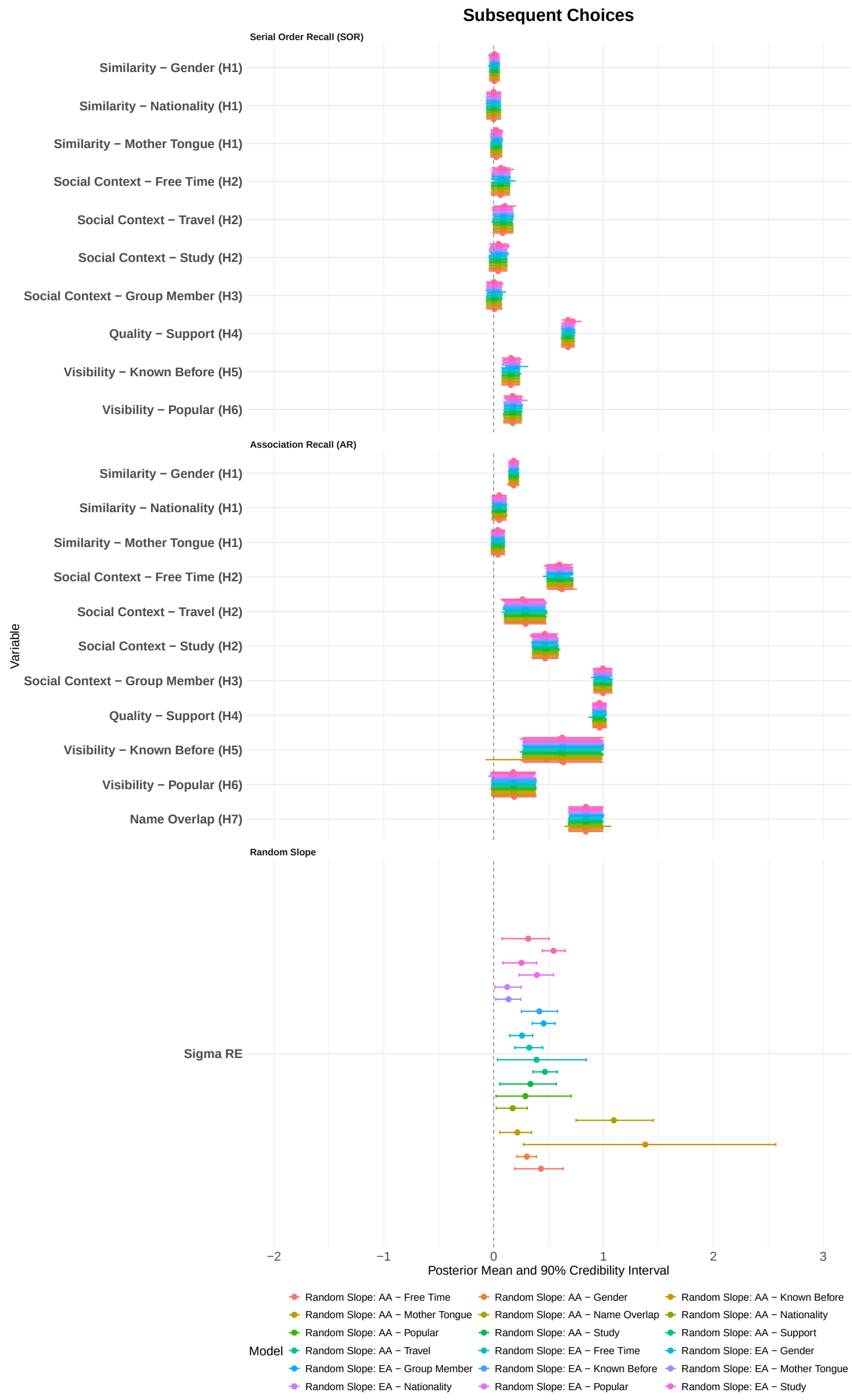

Figure 53. Comparing the base model with alternative models, including a random slope for each predictor separately based on individuals.

16.11 Subsequent Choices - Positive Interaction - MICE imputation

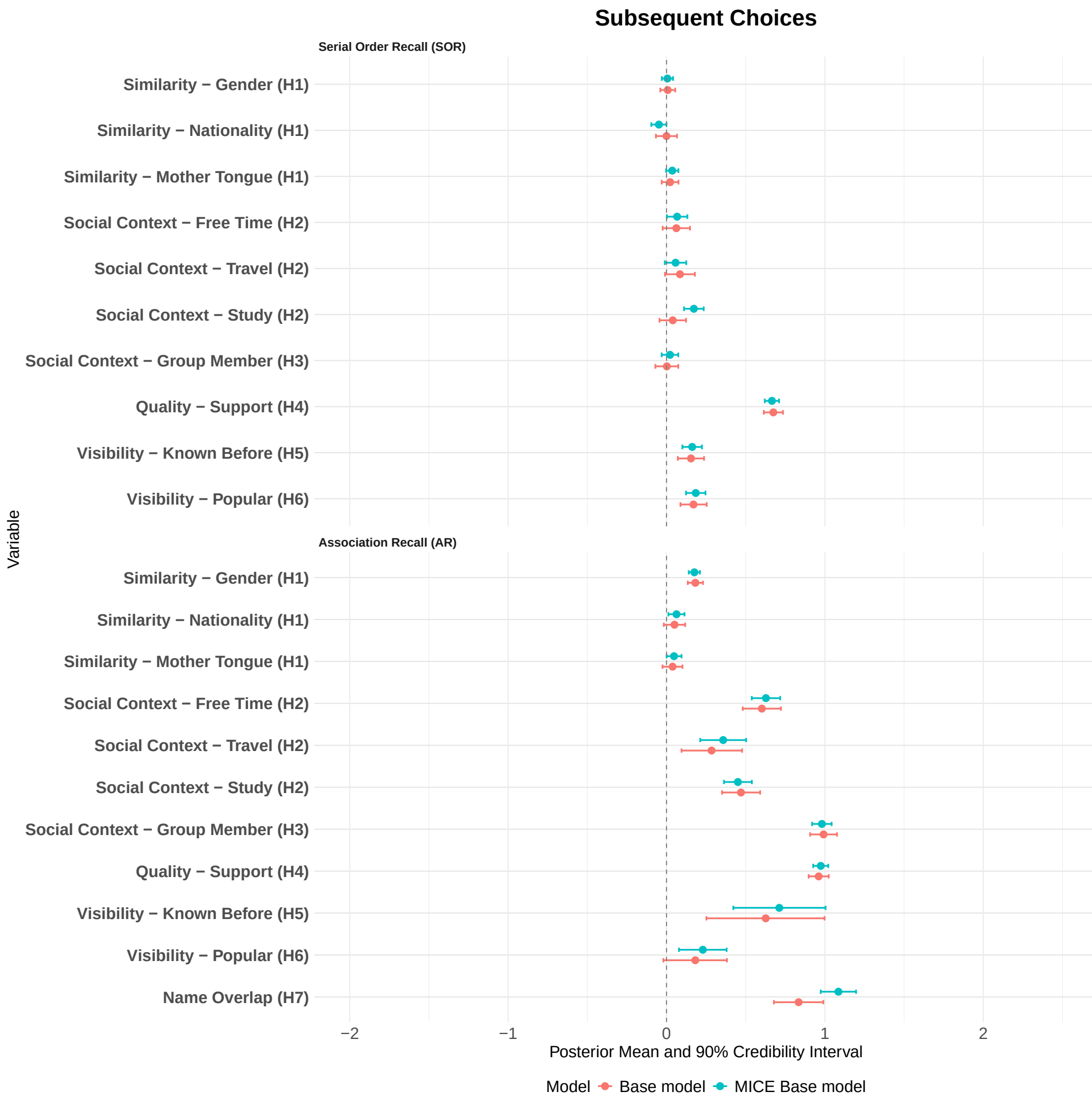

Figure 54. Comparing the base model with an alternative model using the mice imputed datasets.

16.12 Subsequent Choices - Positive Interaction - Reduced Choice set

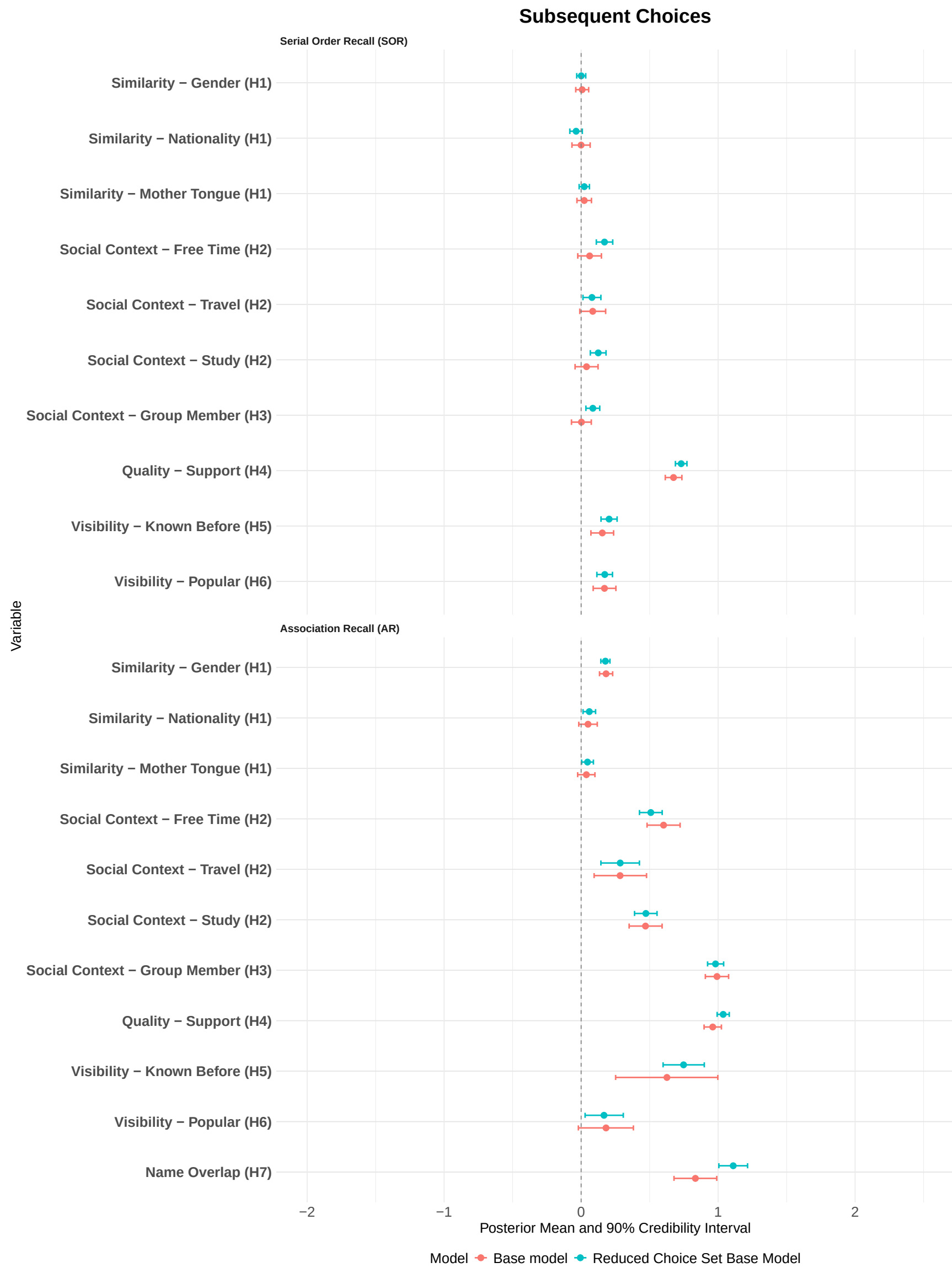

Figure 55. Comparing the base model with an alternative model using the reduced choice sets.

16.13 Subsequent Choices - Positive Interaction - Explorative Robustness Operationalisations

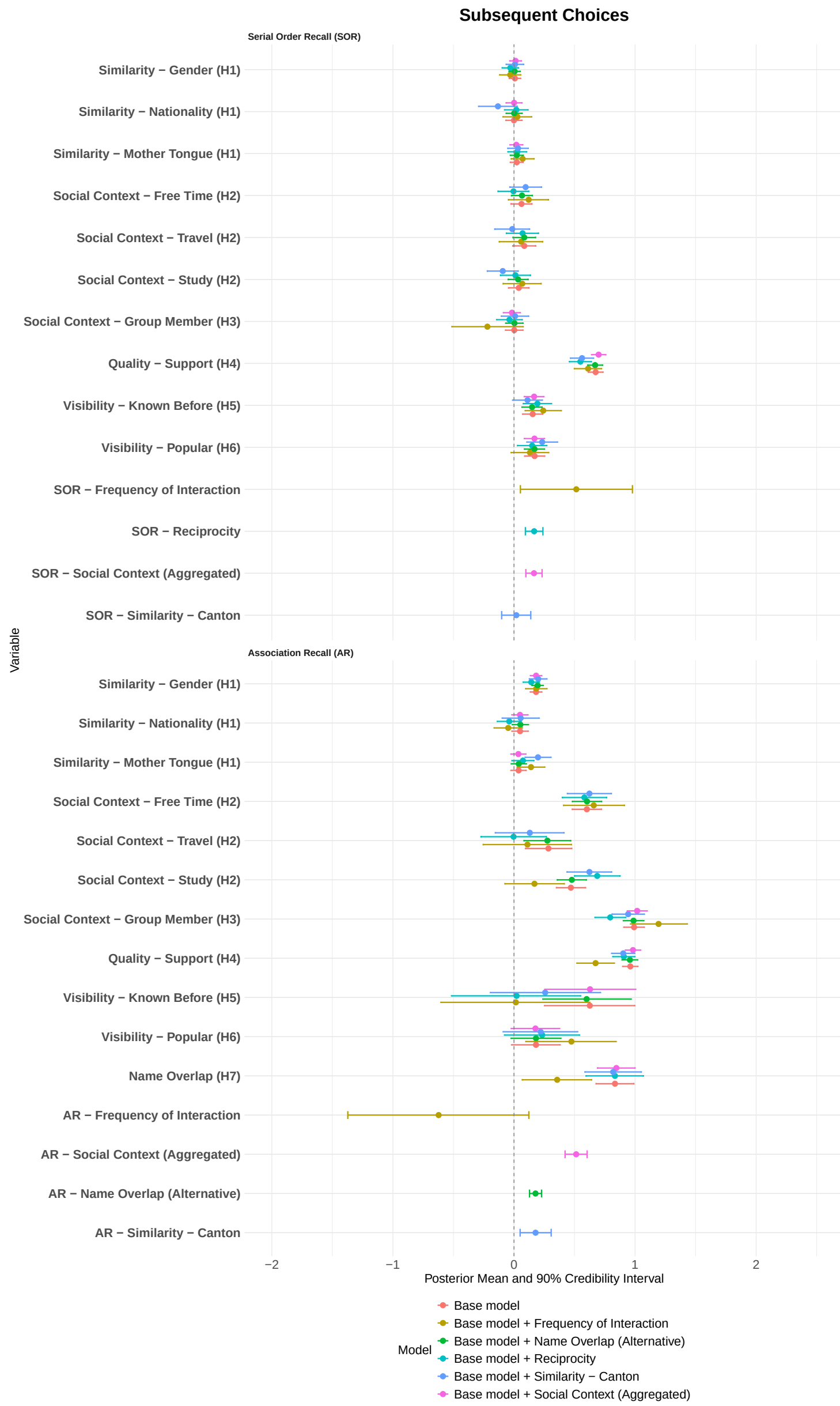

Figure 56. Comparing the base model with alternative models, using different Operationalisations and new predictors.

References

1. Kovacevic, Z. & Stadtfeld, C. Cognitive mechanisms underlying the recall of positive relationships in social networks. OSF Project (2026). https://osf.io/2v4qa/overview Accessed: 2026-06-15.
2. Kovacevic, Z. & Stadtfeld, C. Cognitive mechanisms underlying the recall of positive relationships in social networks. OSF Preregistration, DOI: 10.17605/OSF.IO/6GYZT (2022).
3. Glanzer, M. & Cunitz, A. R. Two storage mechanisms in free recall. *J. Verbal Learn. Verbal Behav.* **5**, 351–360, DOI: 10.1016/S0022-5371(66)80044-0 (1966).
4. Pachur, T. *et al.* Unpacking cognitive search: Mechanisms and processes. In Todd, P. M., Hills, T. T. & Robbins, T. W. (eds.) *Cognitive search: Evolution, algorithms, and the brain*, 237–253 (MIT Press, Cambridge, MA, 2012).
5. Roenker, D. L., Thompson, C. P. & Brown, S. C. Comparison of measures for the estimation of clustering in free recall. *Psychol. Bull.* **76**, 45–48, DOI: 10.1037/h0031355 (1971).
6. Brewer, D. D., Rinaldi, G., Mogoutov, A. & Valente, T. W. A quantitative review of associative patterns in the recall of persons. *J. Soc. Struct.* **6** (2005).
7. Hills, T. T. & Pachur, T. Dynamic search and working memory in social recall. *J. Exp. Psychol. Learn. Mem. Cogn.* **38**, 218–228, DOI: 10.1037/a0025161 (2012).
8. Brewer, D. D. & Yang, B. L. Patterns in the recall of persons in a religious community. *Soc. Networks* **16**, 347–379, DOI: 10.1016/0378-8733(94)90016-7 (1994).
9. Uzaheta, A., Amati, V. & Stadtfeld, C. Random effects in dynamic network actor models. *Netw. Sci.* **11**, 249–266, DOI: 10.1017/nws.2022.37 (2023).
10. Stan Development Team. *Stan User's Guide* (2024).
11. Gelman, A., Lee, D. & Guo, J. Stan: A probabilistic programming language for Bayesian inference and optimization. *J. Educ. Behav. Stat.* **40**, 530–543, DOI: 10.3102/1076998615606113 (2015).
12. Piironen, J. & Vehtari, A. Sparsity information and regularization in the horseshoe and other shrinkage priors. *Electron. J. Stat.* **11**, 5018–5051, DOI: 10.1214/17-EJS1337SI (2017).
13. McLatchie, Y. & Vehtari, A. Efficient estimation and correction of selection-induced bias with order statistics. *Stat. Comput.* **34**, 132, DOI: 10.1007/s11222-024-10442-4 (2024).
14. Wang, K., Ye, X., Pendyala, R. M. & Zou, Y. On the development of a semi-nonparametric generalized multinomial logit model for travel-related choices. *PLOS ONE* **12**, e0186689, DOI: 10.1371/journal.pone.0186689 (2017).
15. Krause, R. W., Huisman, M., Steglich, C. & Snijders, T. Missing data in cross-sectional networks – an extensive comparison of missing data treatment methods. *Soc. Networks* **62**, 99–112, DOI: 10.1016/j.socnet.2020.02.004 (2020).
16. Van Buuren, S. *Flexible Imputation of Missing Data, Second Edition* (Chapman and Hall/CRC, 2018), 2 edn.
17. Zhou, X. & Reiter, J. P. A note on Bayesian inference after multiple imputation. *The Am. Stat.* **64**, 159–163, DOI: 10.1198/tast.2010.09109 (2010).
18. Bürkner, P.-C. brms: An R package for Bayesian multilevel models using Stan. *J. Stat. Softw.* **80**, 1–28, DOI: 10.18637/jss.v080.i01 (2017).
19. McElreath, R. *Statistical Rethinking: A Bayesian Course with Examples in R and Stan* (Chapman and Hall/CRC, 2020), 2 edn., DOI: 10.1201/9780429029608
